# New Diterpenes with Potential Antitumoral Activity Isolated from Plants in the Years 2017–2022

**DOI:** 10.3390/plants11172240

**Published:** 2022-08-29

**Authors:** Cristina Forzato, Patrizia Nitti

**Affiliations:** Department of Chemical and Pharmaceutical Sciences, University of Trieste, Via L. Giorgieri 1, 34127 Trieste, Italy

**Keywords:** diterpenes, higher plants, biosynthesis, cytotoxic activity, cancer cell lines

## Abstract

Diterpenes represent a wider class of isoprenoids, with more than 18,000 isolated compounds, and are present in plants, fungi, bacteria, and animals in both terrestrial and marine environments. Here, we report on the fully characterised structures of 251 new diterpenes, isolated from higher plants and published from 2017, which are shown to have antitumoral activity. An overview on the most active compounds, showing IC_50_ < 20 μM, is provided for diterpenes of different classes. The most active compounds were extracted from 29 different plant families; particularly, Euphorbiaceae (69 compounds) and Lamiaceae (54 compounds) were the richest sources of active compounds. A better activity than the positive control was obtained with 33 compounds against the A549 cell line, 28 compounds against the MCF-7 cell line, 9 compounds against the HepG2 cell line, 8 compounds against the Hep3B cell line, 19 compounds against the SMMC-7721 cell line, 9 compounds against the HL-60 cell line, 24 compounds against the SW480 cell line, and 19 compounds against HeLa.

## 1. Introduction

**Scheme 1:**
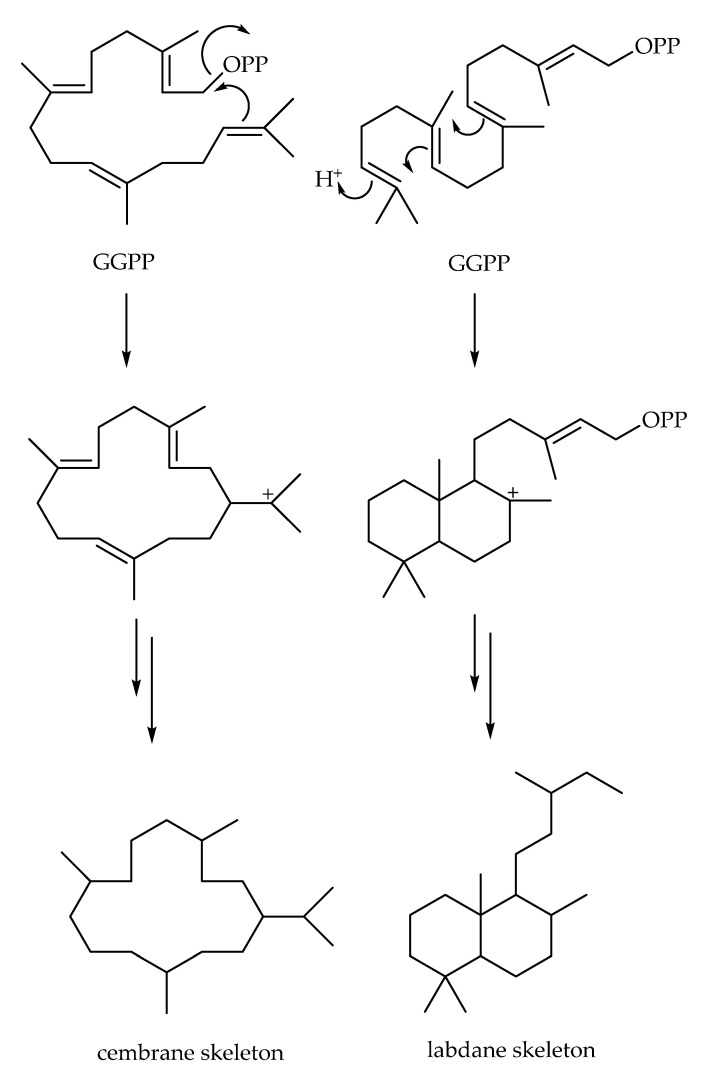
Formation of the skeletons of cembrane and labdane by different GGPP cyclization.

The plant kingdom is the largest reservoir of secondary metabolites, due to the very large number of species present in the world, which synthesise a large variety of molecules, from the simplest ones to the most complex. Most of the secondary metabolites are unique of a single species, but some skeletons are common to different families of plants. Every year, new secondary metabolites are isolated and identified from plants, and their biological activity is evaluated. Isoprenoids represent one of the largest groups of natural products in living organisms, and they derive from the condensation of basic five-carbon units (isoprene units), namely isopentenyl diphosphate (IPP) and dimethylallyl diphosphate (DMAPP). The condensation of one unit of IPP with one unit of DMAPP leads to the formation of geranyl diphosphate, which will be the precursor of monoterpenes. The condensation of further IPP units will then lead to the formation of the precursors of sesquiterpenes (C15) and diterpenes (C20). Diterpenes represent one of the wider families of natural products, with more than 18,000 isolated compounds, which are present in plants, fungi, bacteria, and animals in both terrestrial and marine environments [1]. The isoprene units can derive from both the mevalonate (MVN) and methylerythritol phosphate (MEP) pathways, although it has been observed that the MVN pathway is active in all higher eukaryotes and many bacteria, while the MEP pathway occurs in bacteria and the chloroplasts of green algae and higher plants [2]. Diterpenes are formed from the precursor geranylgeranyl diphosphate (GGPP), which can be cyclised by several cyclases, providing origin to thousands of different diterpens with different skeletons. Diterpenes can be classified as high- or low-grade diterpenoids, depending on the cyclization [3], but they can be also classified according to their parent skeleton, depending on the ring number they contain [4]. This latter classification is used more often and defines diterpenes as acyclic, monocyclic, bicyclic, tricyclic, tetracyclic, macrocyclic, and miscellaneous structures. Apart from acyclic diterpenes, most of the known diterpenes derive from two different parent cyclised structure of the GGPP, namely cembrane and labdane, which can be subsequently modified to form new structures (Scheme 1).

**Scheme 2:**
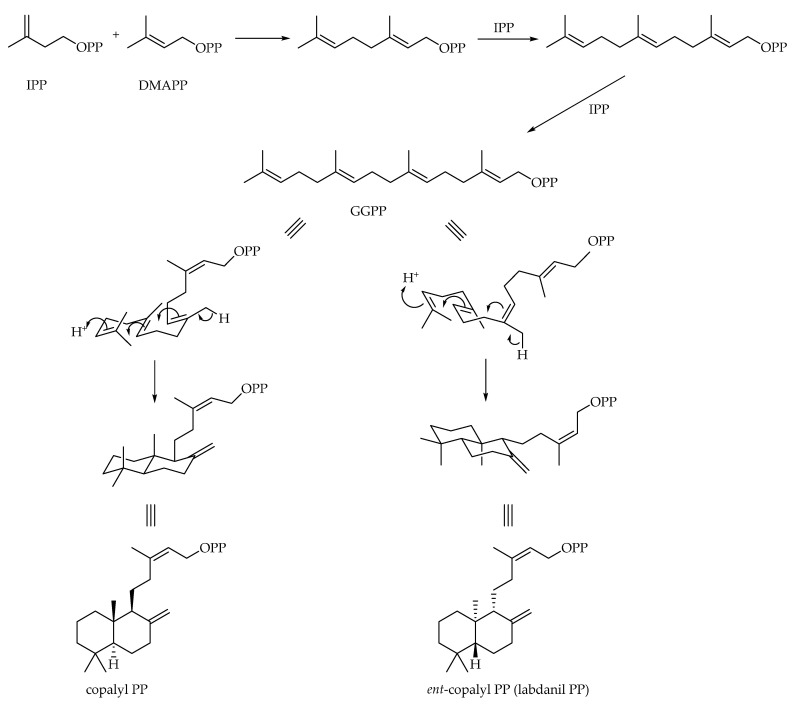
Stereospecific cyclization of GGPP.

Moreover, the cyclisation of GGPP to the labdane structure depends on the conformational folds of GGPP, leading to compounds that belong to the “normal” series (fusion between A and B rings occurs in the same way as in steroids), while compounds that are specular images of the normal series belong to the series “*ent*” (Scheme 2).

In the present review, we summarise the new diterpenes identified and characterised between 2017 and 31 May 2022, highlighting the cytotoxic activity against cancer cells observed for some of these compounds, in order to provide inspiration for novel drugs derived from natural products. To highlight a relation between the structure and cytotoxic activity, we decided to divide the diterpenes into two main groups: the first one containing the monocyclic diterpenes, cembrane, and diterpenes derived from cembrane, with the second one containing the diterpenes derived from the labdane-type, independently of the stereochemistry of cyclization. A second subdivision will be made according on the number of cycles present in the skeleton. Most of the review present in the literature focus on one species of plant or, sometimes, a family plant, while the present review covers all types of diterpenes isolated from different families of higher plants. For this reason, due to the large number of compounds found, we report only the chemical structures of diterpenes, which have shown significant cytotoxic activity against cancer cells, with IC_50_ values below 20 μM, thus highlighting the ones with higher cytotoxic activity, with respect to the positive control. Although diterpenes have a skeleton with 20 carbon atoms, we also reported compounds with a different number of carbon atoms in the skeleton, since they are defined as diterpenoids in the original article and derive from GGPP. Since the present review was designed to help the researchers involved in the development of new anticancer substances, three tables are provided at the end, in which, the compounds are divided by type of skeleton, cancer cells, and family plant.

In Table 1, compounds are divided by type of skeleton, and compounds with a higher activity, with respect to the positive control, are highlighted. In Table 2, compounds with a better activity than the positive control are organised by the type of human organ target and cell line used. Finally, in Table 3, the plant family and species for each compound are reported to highlight the most promising family plant.

## 2. Monocyclic Diterpenes and Diterpenes Derived from Cembrane

### 2.1. Monocyclic Diterpenes

**Figure 1:**
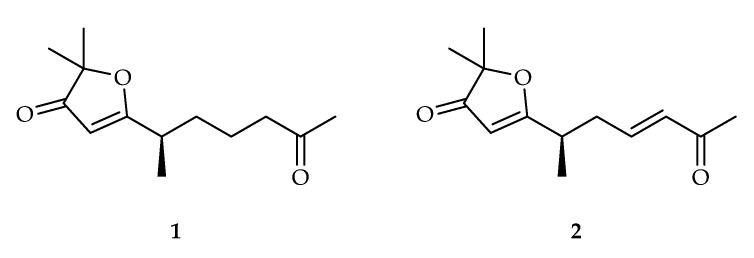
Structure of nornemoralisins A **1** [5] and B **2** [5].

Two novel heptanornemoralisin-type diterpenoids nornemoralisins A **1** and B **2** were isolated from the stem bark and leaves of *Aphanamixis polystachya* (Wall.) R. Parker (Meliaceae), a timber tree mainly growing in the low altitude tropical areas of Asia, such as China, India, Malaysia, and Indonesia [5]. Extraction was performed from the air-dried stem bark and leaves of *A. polystachya* with ethanol and subsequent partitioning with petroleum ether (PE) and ethyl acetate (EtOAc). Their structures were established through comprehensive analyses of NMR spectroscopic data and high-resolution mass spectrometric (HR-ESI-MS) data, while the absolute configurations of carbon stereocenters were elucidated by circular dichroism (CD) analyses. The two compounds were tested for their potential cytotoxic effects against ACHN (human kidney cancer), HeLa (human cervical carcinoma), SMMC-7721 (human hepatoma cancer), and MCF-7 (human breast cancer) cell lines. Nornemoralisin A **1** showed cytotoxicity against ACHN and HeLa cell lines, with IC_50_ values of 13.9 and 19.3 μM, respectively, while nornemoralisin B **2** showed the cytotoxicity against all cancer cell lines used, with IC_50_ values ranging from 1.6 to 11.3 μM. Particularly, nornemoralisins A **1** and B **2** exhibited significant cytotoxicity on ACHN cell line, with an IC_50_ value better than the positive control vinblastine (Figure 1).

**Figure 2:**
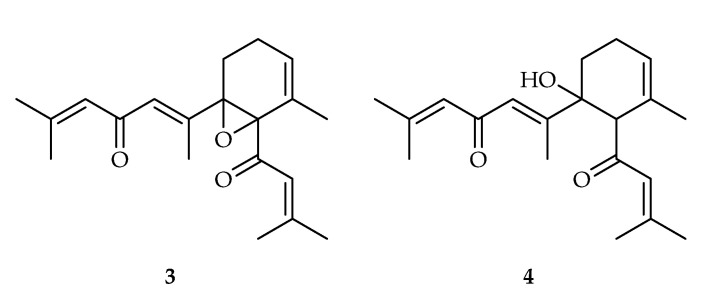
Structures of tagetones A **3** [6] and B **4** [6].

Tagetones A **3** and B **4** (Figure 2), two new monocyclic diterpenoids, were isolated from the *n*-hexane fraction of fresh flowers of *Tagetes minuta* L. (Asteraceae). Their structures were established by multiple spectroscopic methods (IR, HR-ESI-MS, and 1D and 2D NMR). The in vitro cytotoxic activity was determined against a panel of four human cancer cell lines: KB (human epithelial carcinoma), MCF-7, A549 (human non-small-cell lung carcinoma), and HCT116 (colon carcinoma). Tagetone A **3** showed cytotoxic activity with IC_50_ values of 9.39, 4.68, 4.24, and 12.17 μM, respectively, while tagetone B **2** showed IC_50_ values of 16.20, 15.41, 8.29, and 6.30 μM, respectively. Doxorubicin was used as the positive control [6].

### 2.2. Diterpenes Derived from Cembrane

#### 2.2.1. Casbane-Type Diterpenoids

**Scheme 3:**
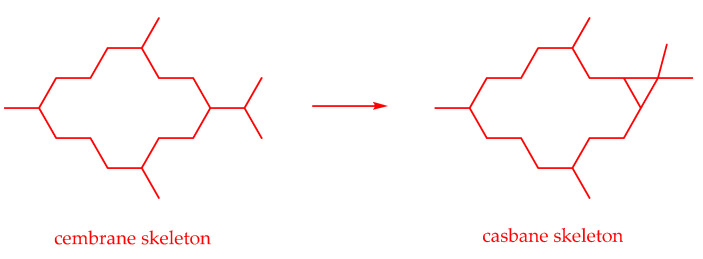
Casbane skeleton formation.

Casbane-type diterpenoids possess a macrocyclic structure fused with a cyclopropane ring, and the two rings are usually *cis*-fused, although a few examples have been found of *trans*-fused [7]. They derive from cembrane skeleton by a second cyclization (Scheme 3).

**Figure 3:**
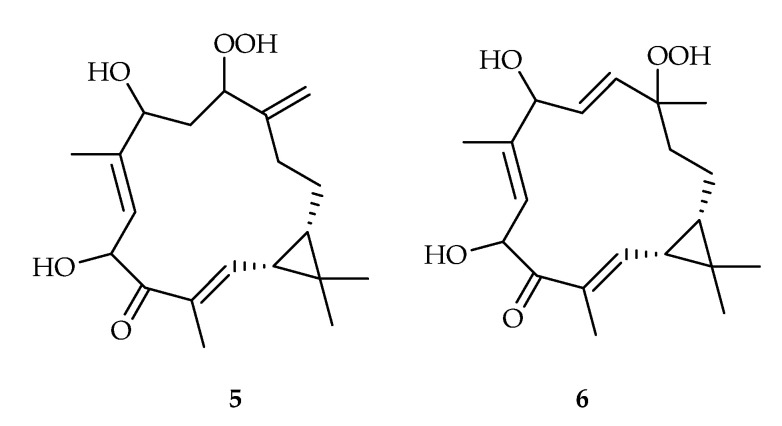
Structures of EBC-304 **5** [8] and EBC-320 **6** [8].

Two casbane hydroperoxide diterpenes, EBC-304 **5** and EBC-320 **6**, were isolated by ethanolic extraction and subsequent partitioning with different solvents from the stems of Australian rainforest *Croton insularis* (Euphorbiaceae) stems [8]. Their structures were determined by spectroscopic analysis, while extensive density functional theory (DFT) and electronic circular dichroism (ECD) calculations, in combination with 2D NMR spectroscopy, determined the absolute configurations. EBC-304 **5** and EBC-320 **6** displayed significant cytotoxicity, with values of IC_50_ in the range of 3–6 μM, on the cancer cell lines HeLa, HT29 (colon cancer), MCF-7, MM96L (melanoma), and K562 (human erythromyeloblastoid leukemia), using the MTS color assay. No positive control was used. Cytotoxicity was observed also for NFF (normal fibroblasts) (Figure 3).

#### 2.2.2. Daphnane-Type Diterpenoids

Daphnane diterpenoids are mainly present in plants of the Thymelaeaceae and Euphorbiaceae families, and their skeleton is formed of a 5/7/6-tricyclic ring system with several hydroxyl groups. About 200 daphnane-type diterpenoids have been isolated and elucidated from these families, which were mainly described in a recent review focusing on their classification and biological activity [9]. Here, we report eight new compounds, isolated from *Daphne genkwa* Sieb. et Zucc. (Thymelaeaceae) and *Euphorbia fischeriana* (Euphorbiaceae), with cytotoxic activity.

**Figure 4:**
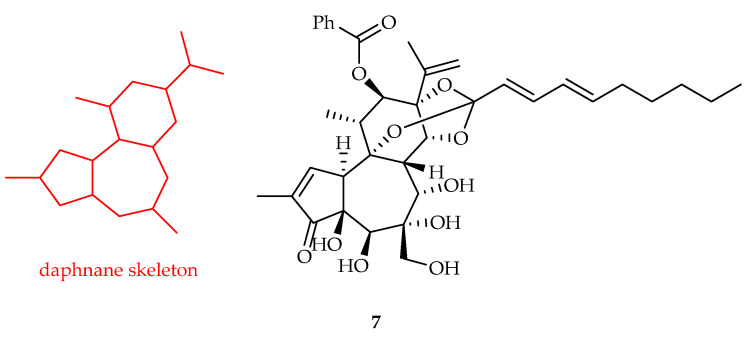
Structure of daphgenkin A **7** [10].

Daphgenkins A-G were isolated from the flower buds of *Daphne genkwa* [10]. Their structures and absolute configurations were elucidated by spectroscopic data and computational ECD analyses. The cytotoxicities of the new daphnane-type diterpenoids were evaluated against three human colon cancer cell lines (SW620, RKO, and LoVo), and daphgenkin A **7** was the most active one, with an IC_50_ value of 3.0 μM against SW620 cells and 6.5 μM against RKO, using 5-fluorouracil (5-FU) as the positive control and MTT assay. Moreover, further studies were performed, and it was demonstrated that compound **7** induced G0/G1 cell cycle arrest, thus leading to the induction of apoptosis in SW620 cells. It also induced cancer cell apoptosis by an increased ratio of Bax/Bcl-2, activated cleaved caspase-3 and caspase-9, and upregulated PARP. Finally, compound **7** significantly inhibited PI3K/Akt/mTOR signaling in SW620 cells. Together, the results suggested that compound **7** may be a suitable lead compound for further biological evaluation (Figure 4).

**Figure 5:**
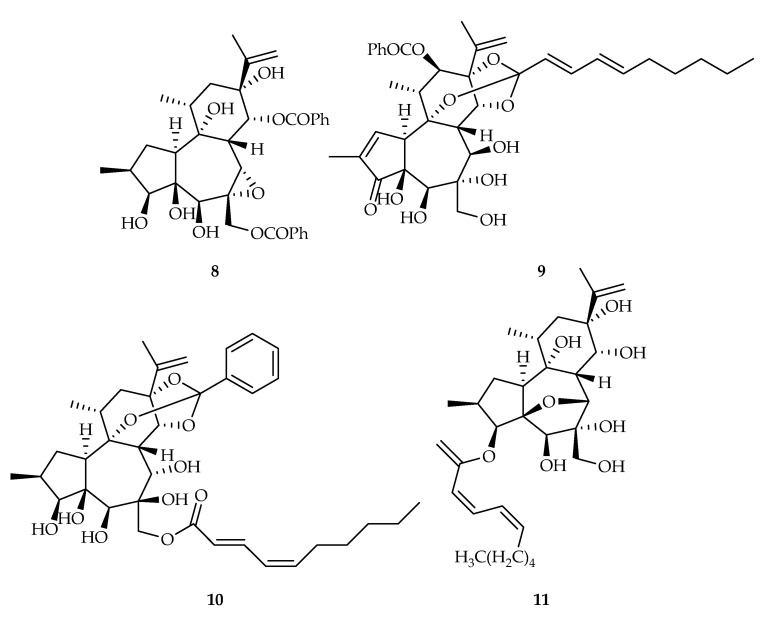
Structures of yuanhuakines A–D **8**–**11** [11].

Four undescribed compounds, yuanhuakines A–D **8**–**11**, were also isolated from the flower buds of *Daphne genkwa* Sieb. et Zucc. by ethanolic extraction and subsequent partitioning with EtOAc and CH_2_Cl_2_ [11]. Their structures were elucidated by spectroscopic analyses, ECD calculations, and single-crystal X-ray diffraction analysis. The new compounds showed inhibitory activity against the A549, Hep3B (human liver cancer), and MCF-7 cell lines, with IC_50_ values ranging from 7.83 to 23.87 μM. The MMT assay was used, and cisplatin, sorafenib, and doxorubicin were used as positive controls (Figure 5).

**Figure 6:**
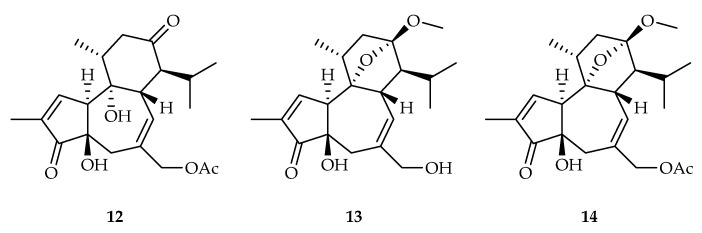
Structures of Langduin A6 **12** [12], fischerianin A **13** [13], and fischerianin B **14** [13].

A new daphnane with cytotoxic activity was isolated from the air-dried root of *Euphorbia fischeriana* Steud. By ethanolic extraction and subsequent partitioning with petroleum ether, CH_2_Cl_2_, EtOAc, and *n*-BuOH [12]. The new compound, named Langduin A_6_ **12**, showed IC_50_ value of 13.42 μM against HepG2 (human hepatocellular liver carcinoma), using the CCK-8 assay and oxaliplatin as a positive control (Figure 6).

From the dry roots of *Euphorbia fischeriana* Steud were also isolated two new daphnane-type diterpenoids fischerianin A **13** and fischerianin B **14** [13]. Their structures, including the absolute stereochemistry, were determined by HRESIMS, IR, 1D and 2D NMR, as well as by comparing their experimental and calculated CD spectra. Compounds **13** and **14** harbor a ketal group and a 9,13-oxide bridge in their C ring, which is rare in daphnane-type diterpenoids. They showed cytotoxic activity against human cancer cell lines A375 (human melanoma cancer), HepG2, HL-60 (human erythroleukemia), K562, and HeLa, with IC_50_ values ranging from 5.31 to 21.46 μM, using cisplatin as a positive control (Figure 6).

#### 2.2.3. Fusicoccane-Type Diterpenoids

**Figure 7:**
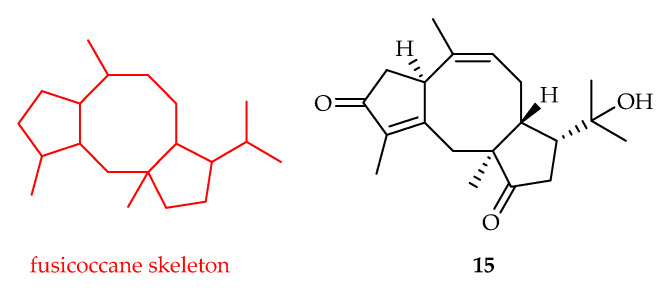
Structure of fusicoccane skeleton and compound **15** [14].

Ten new fusicoccane diterpenes were isolated from the roots of *Hypoestes forsskaolii* (Vahl) R. Br. (Acanthaceae), a perennial bushy and leafy herb widely distributed in several African countries, as well as in the high mountains of the Arabian Peninsula, by extraction with solvents of increasing polarity [14]. The structural characterization of the new compounds was performed by spectroscopic analysis, including 1D and 2D NMR, ECD, and HRESIMS experiments, while their cytotoxic activity was assayed using Jurkat (T-cell lymphoma) and HeLa cancer cells. While all compounds showed IC_50_ values >20 μM for both cancer cell lines, compound **15** demonstrated an IC_50_ value of 18 μM in the HeLa cell line, using etoposide as positive control. Compound **15** did not show cytotoxic activity on the non-tumor human peripheral blood mononuclear cell line (PBMC) up to 100 μM (Figure 7).

#### 2.2.4. Ingenane-Type Diterpenoids

**Scheme 4:**
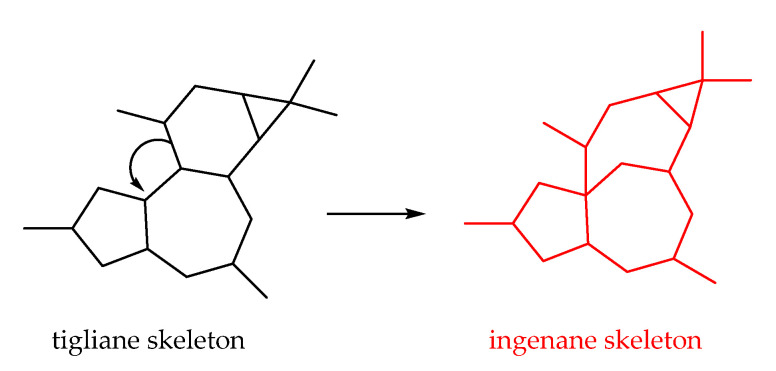
Ingenane skeleton formation.

Ingenane-type diterpenoids are present in the *Euphorbia* genus, and ingenol was the first ingenane diterpene, identified in 1968, in *Euphorbia ingens* E. Mey [1]. The ingenane skeleton is produced through the rearrangement of tygliane skeleton, as shown in Scheme 4 [15].

**Figure 8:**
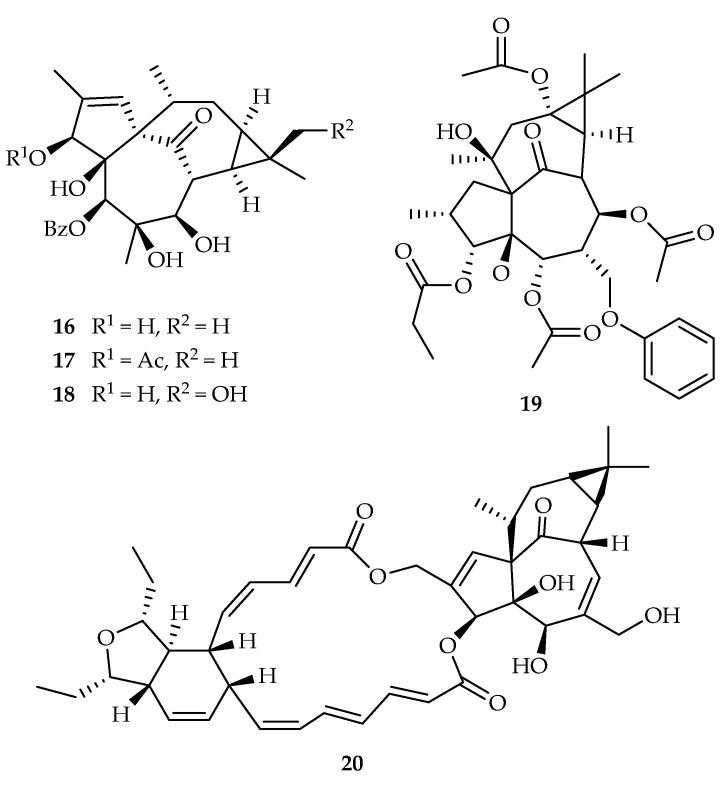
Structures of kansuingenol A-C **16**–**18** [16], compound **19** [17], and euphorkanlide A **20** [18].

Three ingenane-type diterpenes, named kansuingenol A–C **16**–**18** (Figure 8), have been isolated from a 70% ethanol extract of the dried roots of *Euphorbia kansui* S.L.Liou ex S.B.Ho (Euphorbiaceae), a Chinese endemic species mainly distributed in North China [16]. Their structure was elucidated by NMR spectroscopy, while the absolute configurations have been analysed and assigned by the modified Mosher’s, Mo_2_(OAc)_4_-induced circular dichroism (ICD), and CD exciton chirality methods. The new compounds were screened for their antiproliferative effects against HepG2, MCF-7, and DU145 (human prostate cancer) cell lines, and they all showed inhibitory effects on the cell proliferation of these three kinds of cancer cells, with IC_50_ in the range 6.19–29.16 μM, using the CCK-8 assay.

A new tetrahydroingenol diterpene (DANPT) **19** was isolated by dichloromethane/acetone extraction of the aerial parts of *Euphorbia erythradenia* Bioss. (Euphorbiaceae) [17], one of the Iranian endemic spurges, and it was tested for its anti-cancer activity against the A375 and HMCB (human melanoma cancer) cell lines. DANPT **19** was found to be cytotoxic against A375 and HMCB cells, with IC_50_ values of 15.37 μM and 15.62 μM, respectively, using the MTT assay. From further studies and the results obtained, the authors suggest that DANPT can inhibit proliferation of human melanoma cancer cells by promoting apoptosis and inducing cell cycle arrest; therefore, it can be a promising natural agent for the treatment of melanoma cancer (Figure 8).

Euphorkanlide A **20**, a highly modified ingenane diterpenoid incorporating a large (C24) appendage forming an additional 5/6/19 ring system (Figure 8), was isolated from the roots of *Euphorbia kansuensis* (Euphorbiaceae). Compound **20** was screened for cytotoxicity against the HepG2, HepG2/DOX (doxorubicin resistant human liver hepatocellular carcinoma), HCT-15 (human colon cancer), HCT-15/5-FU (fluorouracil resistant human colorectal adenocarcinoma), A549, A549/CDDP (cisplatin resistant human adenocarcinomic alveolar basal epithelial), A375, RKO, and MDA-MB-231 (human breast cancer) cell lines. The results showed that euphorkanlide A **20** inhibited most cancer cell lines at a micromolar level, with IC_50_ values less than 5 μM for the cell lines of HCT-15/5-FU, A375, and RKO. A mechanistic study of **20** on the 5-fluorouracil resistant cell line, HCT-15/5-FU, was further carried out [18].

**Figure 9:**
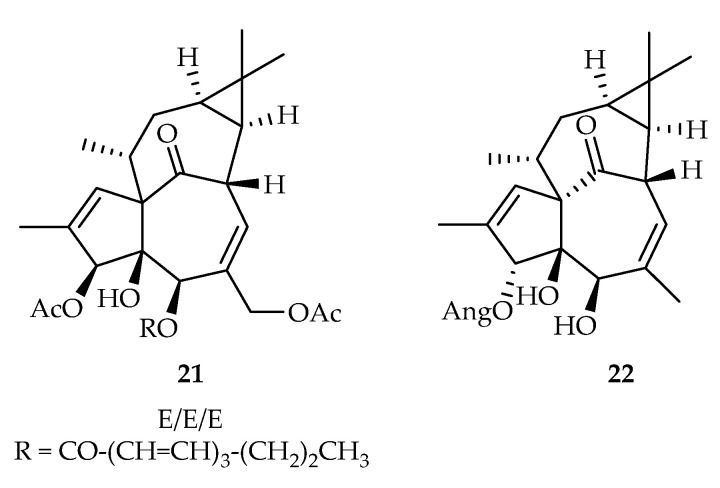
Structures of euphstrachenol C **21** [19] and euphodeflexin L **22** [20].

One new ingenane-type diterpenoid, named euphstrachenol C **21**, together with two new latyrane-type diterpenoids (see Section 2.2.6), was isolated by methanolic extraction and subsequent partitioning in EtOAc, from the roots of *Euphorbia stracheyi* Boiss (Euphorbiaceae), a perennial herb distributed in the Sichuan, Yunan, Tibet, Qinghai, and Gansu provinces of China [19]. Euphstrachenol C **21** was cytotoxic against the MV4-11 (lymphoblast from human biphenotypic B myelomonocytic leukemia) cancer cell line, with an IC_50_ value of 5.92 μM, using the MTT assay and taxol as a positive control (Figure 9).

From *Euphorbia deflexa* (Euphorbiaceae), an endemic spurge from Greece, it was possible to isolate several diterpenes, and three of them proved to be cytotoxic against HeLa cancer cell lines [20]; in particular, one new ingenane-type diterpene, named euphodeflexin L **22**, obtained by methanolic extraction, showed an IC_50_ value of 9.8 μM using the MTT assay and partenolide as a positive control (Figure 9).

#### 2.2.5. Jatrophane-Type Diterpenoids

**Figure 10:**
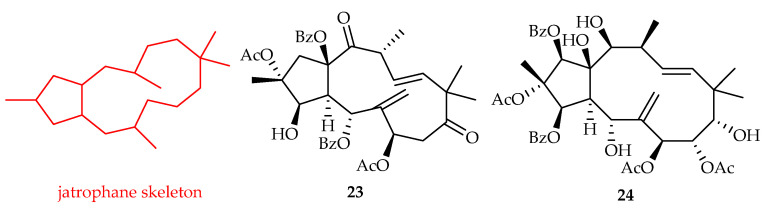
Structures of jatrophane skeleton, compound **23** [21], and euphodeflexin A **24** [20].

Two previously undescribed jatrophane diterpenes with cytotoxic activity were isolated from the roots of *Euphorbia nicaeensis* (Euphorbiaceae), collected in Serbia, by extraction with 96% ethanol and subsequent fractionation by column chromatography [21]. Only compound **23** inhibited cell growth of non-small cell lung carcinoma cell line NCI-H460, as well as glioblastoma cell lines U87 and U87-TxR (resistant glioblastoma), with IC_50_ values between 10 μM and 20 μM, using the MTT assay (Figure 10).

Euphodeflexin A **24** was isolated from the aerial parts of *Euphorbia deflexa* (Euphorbiaceae), together with euphodeflexin L **22** (see Section 2.2.4), with cytotoxic activity against the HeLa cancer cell lines [20]. It showed an IC_50_ value of 9.9 μM using the MTT assay and partenolide as a positive control.

**Figure 11:**
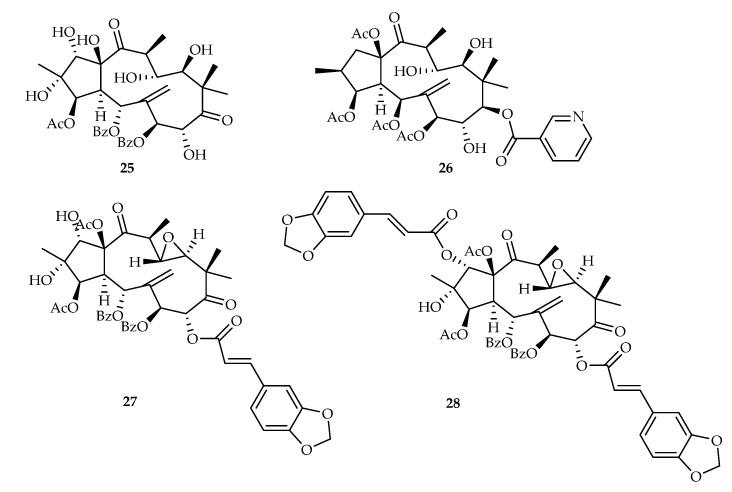
Structures of kansuijatrophanol A-D **25–28** [16].

Yang and coworkers, in 2020, together with the ingenane-type diterpenes, also isolated four jatrophane-type diterpenes, named kansuijatrophanol A-D **25**–**28** (Figure 11), from the ethanolic extract of the aerial parts of *Euphorbia kansui* S. L. Liou ex S. B. Ho (Euphorbiaceae). Their structures, as well as the stereochemistry, were assigned (as already reported in Section 2.2.4) for the ingenane diterpenes [16]. They also showed antiproliferative activities against the MCF-7, HepG2, and DU145 cell lines, with IC_50_ values in the range of 4.19–21.64 μM.

#### 2.2.6. Lathyrane-Type Diterpenoids

**Figure 12:**
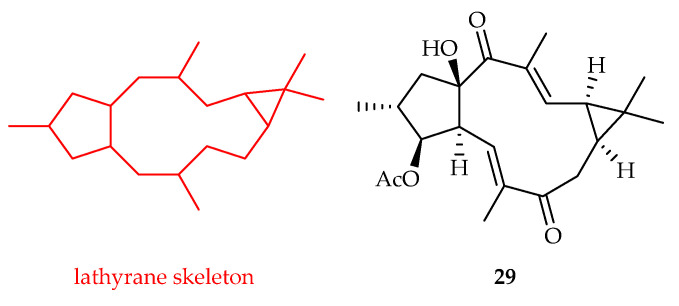
Structures of lathyrane skeleton and jatropodagin A **29** [22].

Two new lathyrane-type diterpenoids, jatropodagins A and B, were isolated from the stems of *Jatropha podagrica* (Euphorbiaceae), a multipurpose shrub commonly found in Africa, Asia, and Latin America, which is used in traditional medicine for various diseases, such as antimicrobial and anti-insect [22]. Their structures and absolute configurations were elucidated by spectroscopic data and computational ECD analyses. The cytotoxicities of the two lathyrane-type diterpenoids were evaluated against two human osteosarcoma cell lines (Saos-2 and MG-63). Jatropodagin A **29** exhibited significant cytotoxic effects against Saos-2 and MG-63, with IC_50_ values of 8.08 and 14.64 μM, respectively, lower than the IC_50_ values for the positive control 5-FU. Morphological features of apoptosis activities were evaluated in compound **29**-treated Saos-2 cells, and the results confirmed apoptosis in a dose-dependent manner (Figure 12).

**Figure 13:**
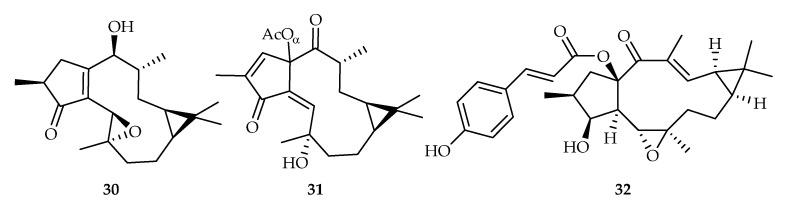
Structures of jatromultone G **30** [23], jatromultone I **31** [23], and euphofischer A **32** [24].

From the air-dried powder of the trunks of *Jatropha multifida* (Euphorbiaceae), an ornamental shrub mainly distributed in South America and South China, it was possible to isolate the new jatromultones A-I. Extraction was performed with 95% ethanol and subsequent partitioning with EtOAc and their structures, including their absolute configurations, were elucidated by combination of spectroscopic analysis, single crystal X-ray diffraction, and chemical evidence. They were screened for antiproliferative activity against a panel of five different cancer cell lines (A549, MDA-MB-231, HepG2, HeLa, and HepG2/DOX), and four of them showed cytotoxic activity. Among them, jatromultone G **30** had an IC_50_ < 20 μM against A549 and HeLa, while jatromultone I **31** had an IC_50_ < 20 μM against only A549. The MTT assay was used, with doxorubicin as a positive control [23] (Figure 13). The other two active diterpenoids will be discussed in Section 2.2.8, since they are rhamnofolane diterpens.

Two new lathyrane diterpenoids, euphofischers A and B, were isolated by EtOAc extraction from the roots of *Euphorbia fischeriana*, whose structures were elucidated by spectroscopic analysis [24]. Only euphofischer A **32** exhibited significant cytotoxicity against the C4-2B (human prostate cancer) cell line, with an IC_50_ value of 11.3 μM, using doxorubicin as positive control. Compound **32** represents a rare example of a lathyrane diterpenoid featuring a 15-*p*-coumaroyl moiety.

**Figure 14:**
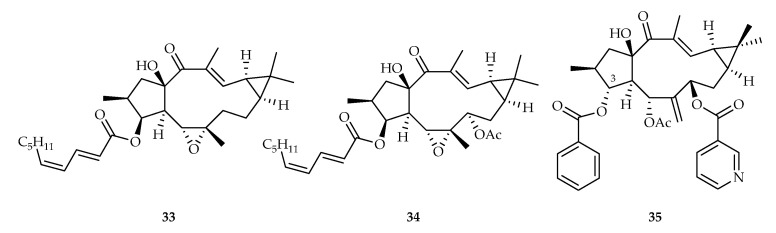
Structures of euphstrachenols A **33** [19] and B **34** [19] and euphorbia factor L_28_ **35** [25].

Euphstrachenols A **33** and B **34** were isolated by methanolic extraction and subsequent partitioning in EtOAc from the roots of *Euphorbia stracheyi* Boiss [19]. Euphstrachenols A **33** and B **34** showed moderate cytotoxicity against the MV4-11 cell line, with IC_50_ values of 12.29 and 14.80 μM, respectively, using the MTT assay and taxol as a positive control (Figure 14).

Euphorbia factor L_28_ **35**, a new lathyrane-type diterpene with a α-oriented substituent at C-3 (Figure 14), was isolated from the seeds of *Euphorbia lathyrism* (Euphorbiaceae). Cytotoxicity assays showed that compound **35** exhibited stronger cytotoxicity against the MCF-7 cell line (IC_50_ 9.43 μM) than classic anticancer drug cisplatin, as well as significant cytotoxicity against HepG2 cell line (IC_50_ 13.22 μM), indicating that esterification at C-3 with α-configuration and substituting with heterocyclic ester group would improve the cytotoxicity of the lathyrane-type diterpene [25].

#### 2.2.7. Pepluane-Type Diterpenoids

**Scheme 5:**
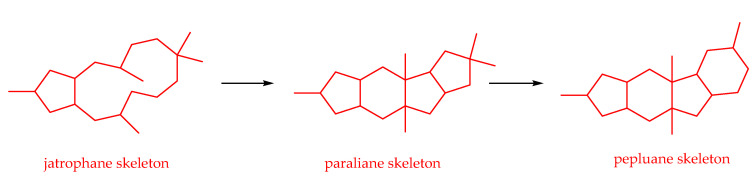
Formation of pepluane skeleton.

Both pepluane and paraliane diterpenes are based on a fused tetracyclic core originated from further rearrangements of a proper jatrophane skeleton. The paraliane skeletons, isolated for the first time from *Euphorbia paralias* in 1998, are rare 5/6/5/5 tetracyclic systems that are probably formed through a transannular ring-closing reaction of the jatrophane diterpene (a jatropha-6(l7),12-diene), thus resulting in a 5/6/5/5-ring system. The introduction of primary alcohol on gem-dimethyl, followed by a complete cycle expansion, results in the formation of the pepluane skeleton (5/6/5/6) [26] (Scheme 5).

**Figure 15:**
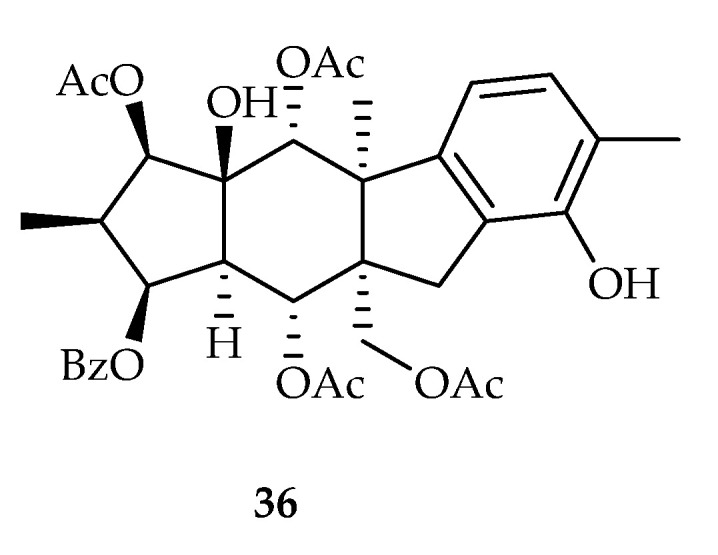
Structure of euphodeflexin O **36** [20].

From the aerial parts of *Euphorbia deflexa*, together with an ingenane (see Section 2.2.4) and jatrophane (see Section 2.2.5), it was possible to isolate also a pepluane diterpene, named euphodeflexin O **36**, which proved to be cytotoxic against HeLa cancer cell lines [20]. Euphodeflexin O **36** had an IC_50_ value of 5.8 μM, using the MTT assay and partenolide as a positive control (Figure 15).

#### 2.2.8. Rhamnofolane-Type Diterpenoids

**Figure 16:**
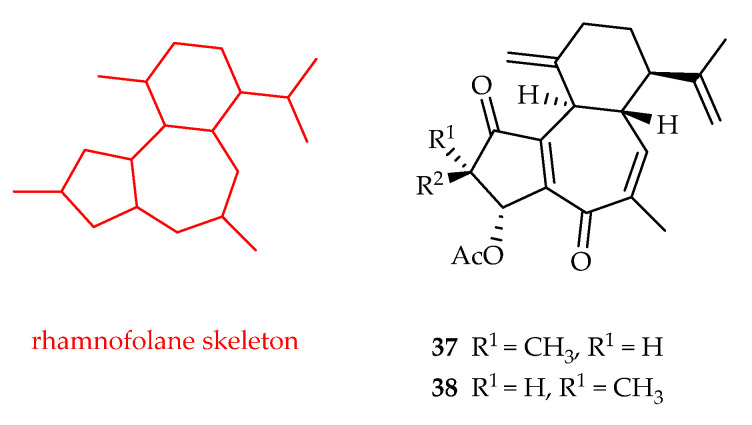
Structures of rhamnofolane skeleton, jatromultone C **37** [23], and jatromultone D **38** [23].

Two rhamnofolane diterpenoids, named jatromultone C **37** and jatromultone D **38**, together with the two latyrane diterpenoids **30** and **31** (see Section 2.2.6), were isolated from the air-dried powder of the trunks of *Jatropha multifida* (Euphorbiaceae) (China) [23]. Compound **38** showed remarkable activity, with IC_50_ values less than 10 μM, against five different cancer cell lines (A549, MDA-MB-231, HepG2, HeLa, and HepG2/DOX), while compound **37** had cytotoxic activity against A549, MDA-MB-231, HepG2, and HepG2-DOX, with IC_50_ values in the range of 2.69–6.44 μM. Doxorubicin as a positive control and the MTT assay were used (Figure 16).

#### 2.2.9. Tigliane-Type Diterpenoids

**Scheme 6:**
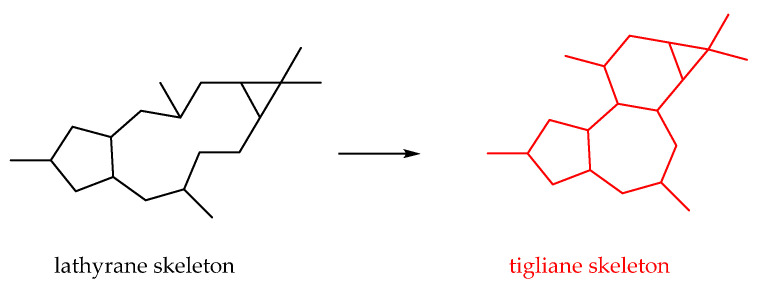
Formation of tigliane skeleton.

Tigliane diterpenoids are based on a 5/7/6/3 tetracyclic ring system, and the skeleton derives from the cyclization of lathyrane diterpenoids [27] (Scheme 6).

**Figure 17:**
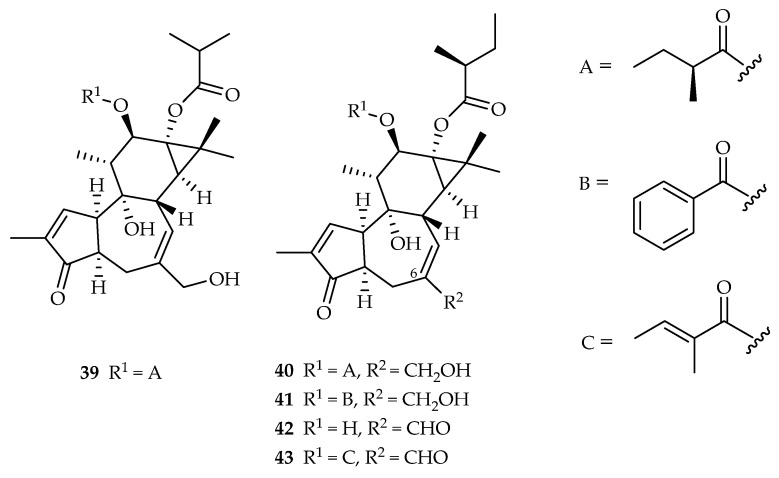
Structures of crodamoid A **39**, crodamoid C **40**, crodamoid D **41**, crodamoid H **42**, and crodamoid I **43** [28].

New tigliane diterpenoids, crodamoid A **39**, crodamoid C **40**, crodamoid D **41**, crodamoid H **42**, and crodamoid I **43** (Figure 17), were isolated from the aerial parts of *Croton damayeshu* (Euphorbiaceae), which is mainly distributed in Yunnan Province, China. The structural elucidation of these compounds was conducted by comprehensive analyses of the NMR, single-crystal X-ray crystallography, and ECD data. Cytotoxic tests revealed that compounds **42** and **43** exhibited significant activities against the A549 and HL-60 cell lines (IC_50_ 0.9–2.4 μM). Particularly, compound **42** displays a selective activity comparable to adriamycin against the A549 cell line. Compound **39** showed activity against A549, with IC_50_ value of 3.7 μM, while compounds **40** and **41** showed activities against both the tested cancer cell lines, with IC_50_ values ranging from 4.1 to 22.7 μM [28]. The presence of a formyl group at C-6 seemingly favors the cytotoxic activities of this compound class.

**Figure 18:**
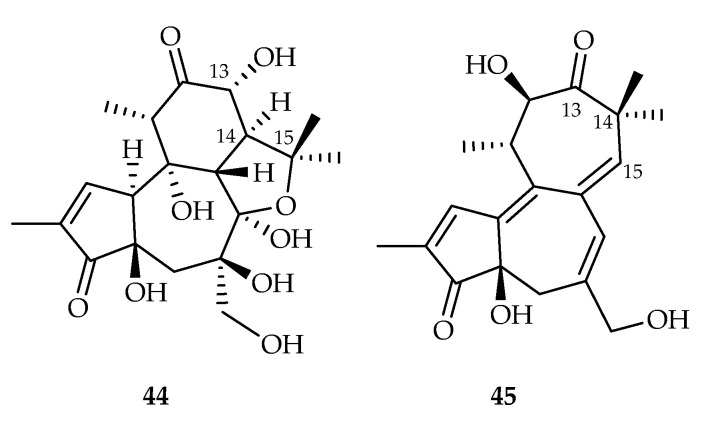
Structures of crotonol A **44** [29] and crotonol B **45** [29].

Crotonol A **44** and crotonol B **45** were isolated from the leaves of *Croton tiglium* (Euphorbiaceae), and crotonol B **45** represents the first example of a 13,14-*seco*-tigliane diterpenoid (Figure 18). Crotonols A **44** and B **45** were evaluated in vitro for their cytotoxicity against the K562, MCF-7, and SGC-7901 (human gastric cancer) cell lines, with paclitaxel as a positive control. Compounds **44** and **45** showed strong cytotoxic activities (IC_50_ 0.20 and 0.21 μM, respectively), better than paclitaxel, towards the K562 cell line, while exhibiting low cytotoxicity in the MCF-7 and SGC-7901 cancer cell lines [29].

#### 2.2.10. Taxane-Type Diterpenoids

**Figure 19:**
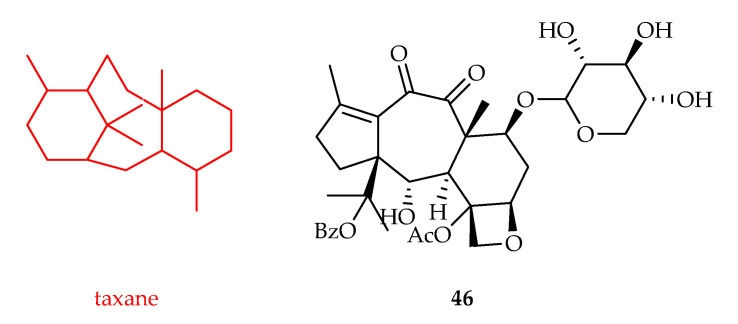
Structure of wallitaxane E **46** [30].

Nguyen et al. isolated six new taxoids, i.e., wallitaxanes A-F, from the bark of *Taxus wallichiana* (Taxaceae) by extraction with dichloromethane [30]. A detailed spectroscopic analysis was performed to assign the correct structure to the new compounds. All the isolated compounds were evaluated for cytotoxicity against the HeLa cell line and among the new compounds; only wallitaxane E **46**, which is a representative of the rare *abeo*-taxoid class, showed cytotoxic activity with IC_50_ value of 4.9 μM, although paclitaxel, which was used as the positive control, has an IC_50_ value of 0.0014 μM. Note that the two known compounds 7-*epi*-taxol and 7-*epi*-10-deacetyltaxol, which were also isolated, showed IC_50_ values of 0.05 and 0.085 nM, respectively, against the HeLa cells (Figure 19).

## 3. Labdane-Type Diterpenes

### 3.1. Bicyclic Diterpenes

#### 3.1.1. Clerodane-Type Diterpenoids

**Figure 20:**
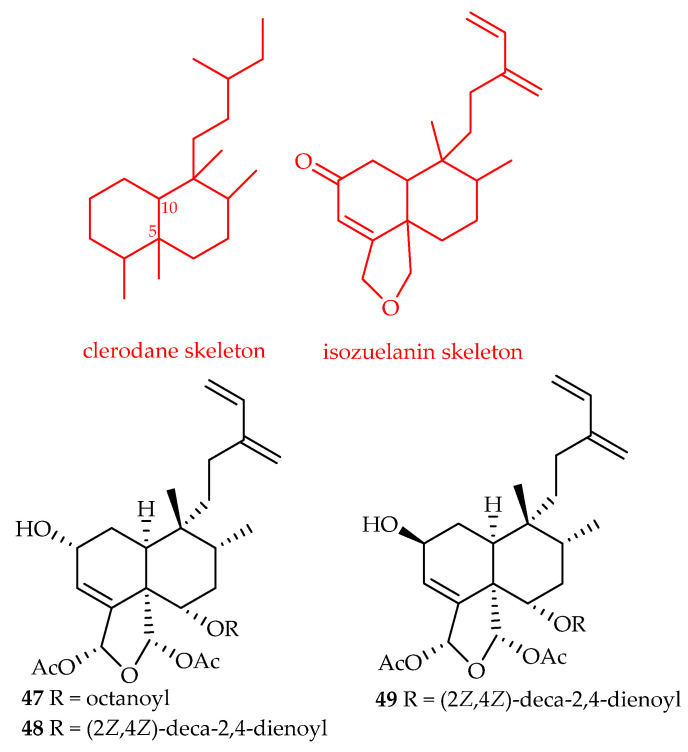
Structure of corymbulosins D–F **47–49** [32].

The clerodane skeleton contains a decalin ring mojety that can have a *cis* or *trans* ring fusion. Approximately 25% of clerodanes have a 5:10 *cis* ring, while the remaining 75% of clerodanes have a 5:10 *trans* ring fusion (Figure 20). Clerodanes with a 5:10 *trans* ring junction are characteristic of the Lamiaceae and, to a lesser extent, Compositae (Asteraceae) families, while clerodanes with a 5:10 *cis* ring junction are more commonly found in the Euphorbiaceae, Flacourtiaceae (Salicaceae), and Menispermaceae families. A description of the clerodane structure and nomenclature can be found in the review of Lee et al. published in 2016 [31].

Clerodanes with an isozuelanin skeleton were found by extraction of the bark of *Laetia corymbulosa* (Salicaceae) with CH_3_OH–CH_2_Cl_2_ (1:1), which provided five new clerodane diterpenes, designated as corymbulosins D–H [32] (Figure 20). The structures of the new compounds were characterised on the basis of 1D and 2D NMR spectroscopic and HRMS analyses. The absolute configurations were verified through chemical methods, ECD experiments, and spectroscopic data comparison. The antiproliferative activity against a small panel of human cancer cell lines (A549, MDA-MB-231, MCF-7, KB, and KB-VIN (P-gp-overexpressing MDR subline of KB)) was evaluated, and corymbulosins D–F **47**–**49** exhibited antiproliferative activity, with IC_50_ values of around 5 μM, using paclitaxel as a positive control (Figure 20).

**Figure 21:**
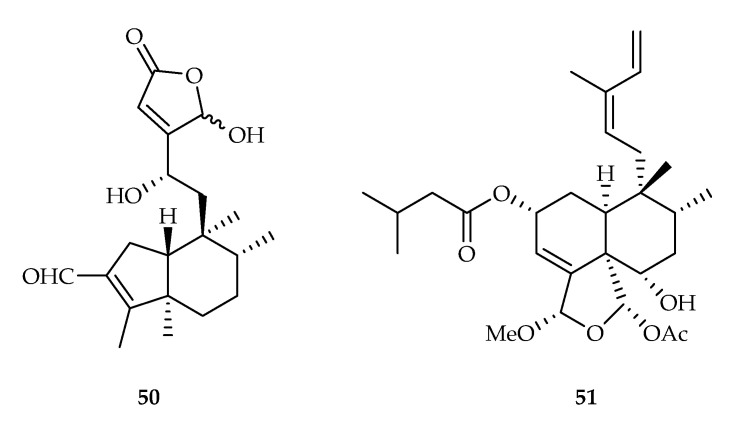
Structures of 16-hydroxy-pentandralactone **50** [33] and caseargrewiin M **51** [34].

16-Hydroxy-pentandralactone **50**, defined by the authors as a clerodane diterpene, was isolated by extraction with methanol of *Vitex cofassus* leaves (Lamiaceae) [33] (Figure 21). The chemical structure and absolute configuration of the new compound were determined by MS, NMR, and ECD experiments. Its antiproliferative activity against a panel of human cancer cell lines (A549, MDA-MB-231, MCF-7, KB, and KB-VIN), including a multidrug-resistant (MDR) cell line, was tested, showing potent antiproliferative activity against all the tested cell lines, with IC_50_ values ranging between 6.4 and 11.4 μM, using paclitaxel as a positive control.

Methanolic extraction and subsequent partitioning with EtOAc of the fresh ripe fruits of *Cesearia grewiifolia* (Flacourtiaceae) led to the isolation of the new clerodane diterpene, which was named caseargrewiin M **51** [34] (Figure 21). Its structure was elucidated using basic 1D (^1^H and ^13^C) and 2D NMR (COSY, HSQC, HMBC, and NOESY) techniques. Cytotoxic activity of caseargrewiin M **51** was determined towards Hep-G2, SW-620, Chago-K1 (undifferentiated lung carcinoma), KATO-III (gastric carcinoma), and BT474 (breast ductal carcinoma) cell lines, using the MTT colorimetric method and doxorubicin as a positive control. It exhibited significant cytotoxicity, with IC_50_ values of 12.84 μM for BT474, 12.43 μM for Chago-K1, 9.46 μM for Hep-G2, 11.21 μM for KATO-III, and 11.21 μM for SW-620.

**Figure 22:**
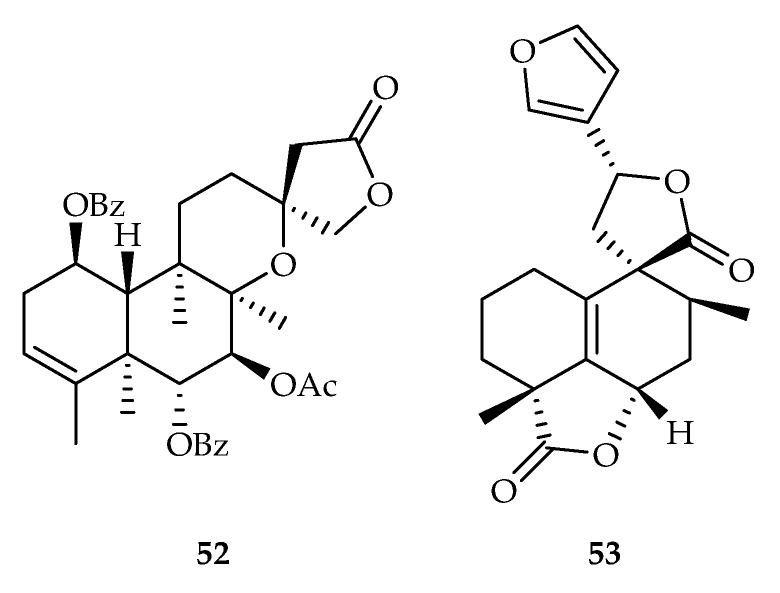
Structures of scubatine F **52** [35] and crassifolius A **53** [36].

Six new *neo*-clerodane diterpenoids, scubatines A–F, were isolated from 70% acetone extract of the whole plant of *Scutellaria barbata* D. Don (Lamiaceae), an herb widely distributed in mainland China, Korea, India, and other Asian countries [35]. Their structures were elucidated on the basis of extensive spectroscopic analyses. Cytotoxic activity against the HL-60 and A549 cell lines was assessed for all new compounds, but only scubatine F **52** showed cytotoxic activity against A549 and HL-60, with IC_50_ values of 10.4 and 15.3 μM, respectively, using the MTT and SRB methods and cisplatin as a positive control (Figure 22).

Three new clerodane diterpenoids, named crassifolius A–C, were isolated by ethanolic extraction of powdered roots of *Croton crassifolius* (Euphorbiaceae), a monecious undershrub widely distributed throughout south and southeast China, Thailand, Vietnam, and Laos [36]. Their structures were established by 1D NMR, 2D NMR, HR-ESIMS, detailed calculated ECD spectra, and the assistance of quantum chemical predictions (QCP) of ^13^C NMR chemical shifts. The cytotoxic activity of the new compounds was tested against human liver cancer cell lines (HepG2 and Hep3B). Among them, crassifolius A **53** exhibited good cytotoxicity, with IC_50_ value of 17.91 μM against human liver cancer cells Hep3B, using MTT assay and 5-FU as a positive control (Figure 22). Further studies of the anti-tumor mechanism of this compound revealed that it caused apoptotic cell death in Hep3B cells by detecting morphologic changes and Western blotting analysis.

**Figure 23:**
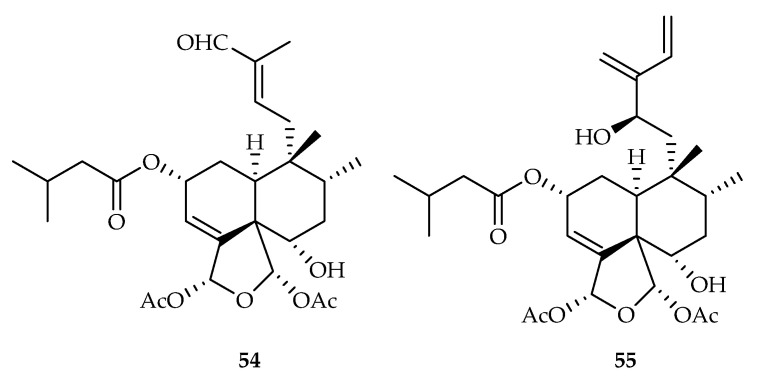
Structures of caseakurzin B **54 [37]** and caseakurzin J **55 [37]**.

Ten clerodane diterpenoids, caseakurzins A–J, were isolated by ethanolic extraction and subsequent partitioning from the twigs and leaves of *Casearia kurzii* (Flacourtiaceae) [37]. The structures of the new compounds were established on the basis of extensive spectroscopic data. They were evaluated for their inhibitory effect against A549 cell line, using cisplatin as positive control. In particular, caseakurzin B **54** and caseakurzin J **55** exhibited significant inhibitory activity, with IC_50_ values of 4.4 and 4.6 μΜ, respectively (Figure 23). Moreover, the effects of compounds **54** and **55** on apoptosis and staining with annexin V/PI, followed by a flow cytometry assay, showed that the apoptotic cells accumulated from 13.93% to 38.97% when treated with 20 μM of compound **54**. For those treated with compound **55** (20 μM), the apoptosis rate increased from 13.57% to 46.14%. The results showed that compounds **54** and **55** arrested the cell cycle at the G2/M and S phases, respectively, and induced apoptosis of the A549 cells.

**Figure 24:**
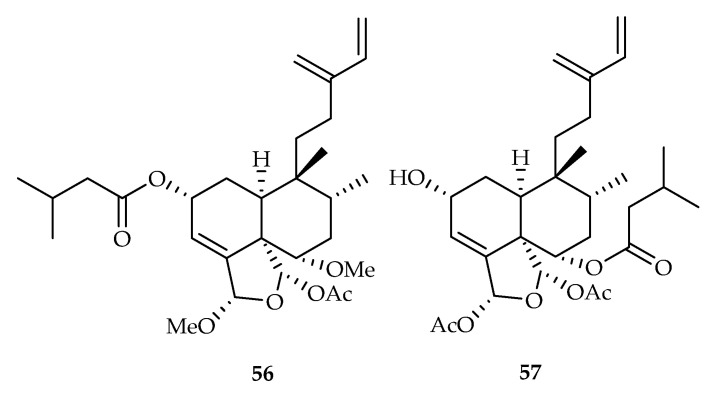
Structures of kurzipene A **56** [38] and kurzipene B **57** [38].

Further, six new clerodane diterpenoids, designated as kurzipenes A–F, were isolated by methanolic extraction and subsequent partitioning with EtOAc from the leaves of *Casearia kurzii* [38]. Their structures were elucidated on the basis of NMR spectroscopic data analysis, while the absolute configurations were assigned by the time-dependent density functional theory (TDDFT) electronic circular dichroism (ECD) calculations. The cytotoxic activities of the new compounds were evaluated against A549, HeLa, and HepG2 cell lines. Kurzipenes D–F were weakly effective (IC_50_ values > 60 μM), while kurzipene A **56** and kurzipene B **57** showed promising activities, with IC_50_ values ranging from 5.3 to 19.0 μM, compared to the positive control etoposide. Compound **57**, with an IC_50_ value of 5.3 μM, was particularly cytotoxic against HeLa cells (Figure 24).

**Figure 25:**
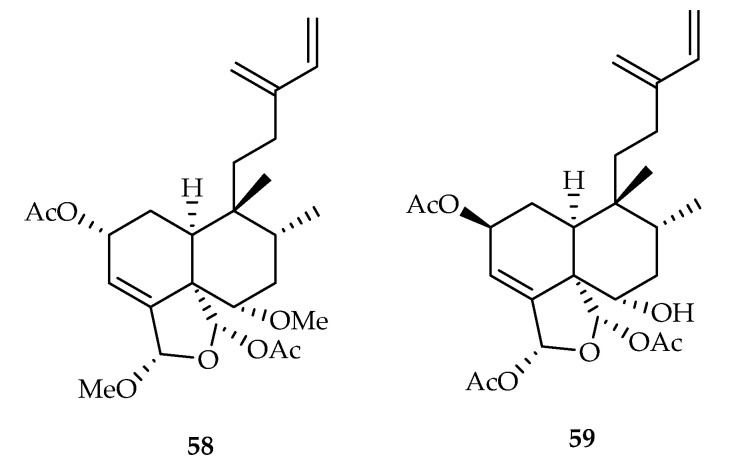
Structures of compounds **58** [39] and **59** [40].

The clerodane diterpene **58** was also isolated from the leaves of *Casearia kurzii* by Guo et al., with a very similar structure to kurzipene C, which showed cytotoxic activity towards HeLa cancer cells with IC_50_ of 17.9 μM, using etoposide as a positive control [39] (Figure 25).

Guo et al., one year later, isolated further six new clerodane diterpenes, which were also named kurzipenes A–F, although they have a different structure [40]. Their cytotoxic activity was evaluated against four cell lines (A549, K562, HeLa, and HepG2) using the MTT assay and etoposide as a positive control. Only compound **59** showed promising activities with IC_50_ values < 20 μM against all cancer cell lines used (Figure 25).

**Figure 26:**
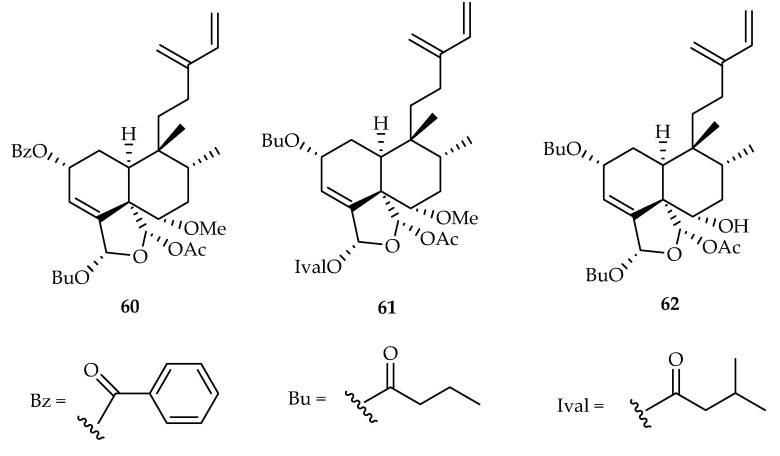
Structures of kurziterpene B **60**, kurziterpene D **61**, and kurziterpene E **62** [41].

The same authors also isolated five other new clerodane diterpenoids from the twigs of *Casearia kurzii*, named kurziterpenes A-E, which exhibited cytotoxic activities against the A549, HeLa, and HepG2 cell lines [41]. Only kurziterpene B **60**, kurziterpene D **61**, and kurziterpene E **62** showed IC_50_ values lower than 20 μM, using etoposide as a positive control. All three compounds have an IC_50_ value lower than the positive control against the HeLa cells (Figure 26).

**Figure 27:**
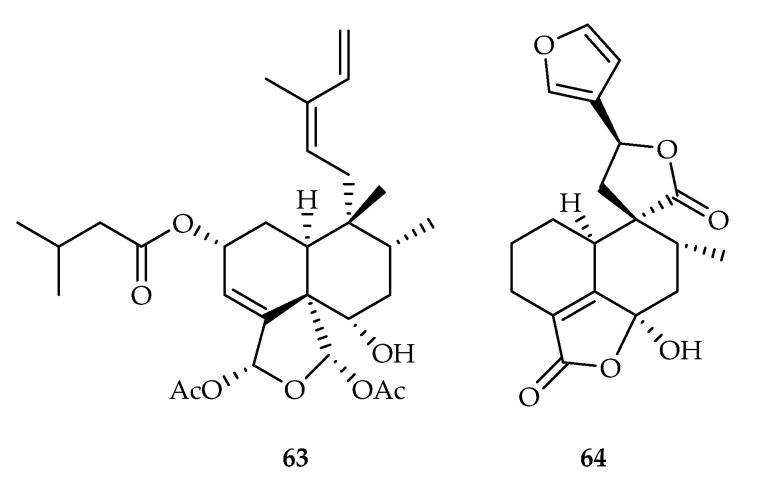
Structures of graveospene A **63** [42] and cracroson F **64** [43].

Nine new clerodane diterpenoids, named graveospenes A–I, were isolated from the leaves of *Casearia graveolens* Dalzell (Flacourtiaceae), a deciduous tree distributed principally in Yunnan Province of mainland China, northern Myanmar, and India [42]. Extraction was performed with methanol and subsequent partitioning with petroleum ether and EtOAc. Spectroscopic methods were employed to establish the structures, with their absolute configurations being confirmed by ECD data analysis. The cytotoxic activity was tested against the A549 and HepG2 cell lines. Graveospene A **63** was shown to be the most potent, with an IC_50_ value of 1.9 μM against A549 cells and IC_50_ value of 4.6 μM against HepG2 cells, using homoharringtonine as a positive control (Figure 27). The IC_50_ values of the other compounds against these two cell lines were >10 μM. A mechanism-of-action study on **63** revealed this compound to induce apoptosis of A549 cells and impede them at the G0/G1 stage.

Three previously undescribed clerodane terpenoids, named cracrosons E–G, were isolated by methanolic extraction of *Croton crassifolius* Geisel (Euphorbiaceae) roots, which were mixed with diatomite and then eluted with different solvents, such as petroleum ether, CHCl_3_, and EtOAc [43]. Their structures were elucidated on the basis of HRESIMS, NMR, and single-crystal X-ray diffraction analyses, along with CD calculations. Moreover, one new diterpenoid, named cracroson D, with an undescribed carbon skeleton was also isolated and is reported in Section 5.3. Only cracroson F **64** showed an IC_50_ value lower than 20 μM against the T24 cell line (human urinary bladder cancer), using the CCK-8 method and gemcitabine as the positive control (Figure 27).

**Figure 28:**
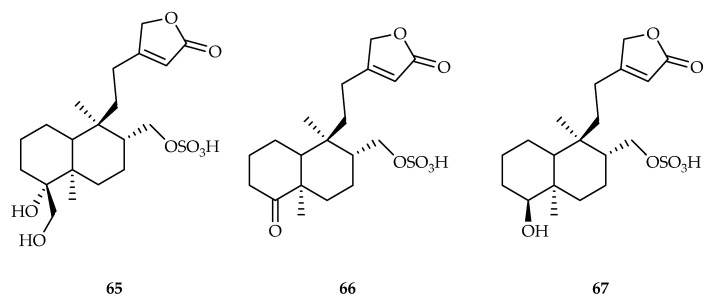
Structures of sheareria A–C **65**–**67** [44].

Sheareria A–C **65**–**67**, three novel *neo*-clerodane diterpenoids, were isolated from the whole herb of *Sheareria nana* (Asteraceae) distributed in northeast, southwest, and central-south of China (Figure 28). Compound **65** was the first natural sulfated *neo*-clerodane diterpenoid, while compounds **66** and **67** were the first natural sulfated 18-nor-*neo*-clerodane diterpenoids. All isolated compounds were evaluated for their cytotoxicity against the human HeLa, PANC-1 (human pancreatic), and A549 cell lines. Compounds **65** and **66** exhibited broad-spectrum and moderate to potent cytotoxicity, with IC_50_ values below 10.0 μM; compound **67** exhibited more selective activity against the PANC-1 cell line (IC_50_ 9.8 μM) than against the A549 and HeLa cell lines (IC_50_ values of 12.5 and 17.2 μM, respectively), using etoposide as a positive control [44].

**Figure 29:**
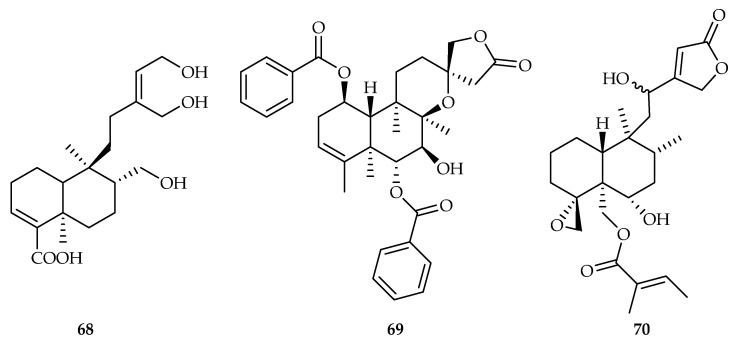
Structures of sheareria D **68** [45], scutebata C_1_ **69** [46], and ajugacumbin N **70** [47].

Sheareria D was further isolated from the whole plant of *Sheareria nana* S. Moore, and its structure was elucidated by spectroscopic data [45]. The extraction was performed with ethanol 95% and subsequent partitioning with petroleum ether and ethyl acetate. Sheareria D **68** exhibited excellent cytotoxicity against human endometrial carcinoma cells (ECC-1), with IC_50_ values of 5.6 and 2.2 μM for 24 and 48 h, respectively, and it showed no cytotoxicity against normal cells. The MTT assay was used (Figure 29).

Scutebata C_1_ **69** was isolated from the aerial parts of *Scutellaria barbata* (Lamiaceae) (Figure 29) and showed cytotoxic activity against SGC-7901 cell line, with an IC_50_ value of 17.9 μM, using cisplatin as a positive control [46].

Ajugacumbin N **70**, a *neo*-clerodane diterpenoid, was isolated from the whole herbs of *Ajuga decumbens* (Labiatae). Compound **70** showed cytotoxic activity against HepG2 cell line (IC_50_ 19.7 μM), using doxorubicin as a positive control [47] (Figure 29).

**Figure 30:**
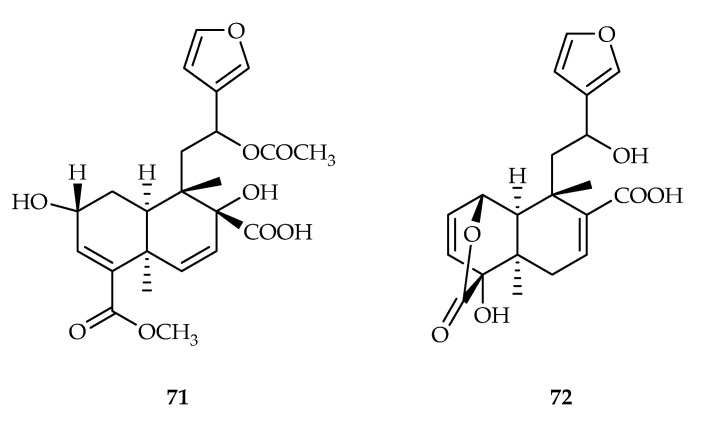
Structures of crispene F **71** [48] and crispene G **72** [48].

Two new *cis*-clerodane-type furanoditerpenes, crispenes F **71** and G **72**, with STAT3 (signal transducer and activator of transcription protein 3) inhibitory activity, were isolated from the stems of *Tinospora crispa* (Menispermaceae) (Figure 30). Compounds **71** and **72** showed IC_50_ values of 10 and 7.8 μM against the MDA-MB-231 (STAT3-dependent) cell line, respectively. The results suggested a STAT3-specific inhibition. The compounds were also tested against a non-tumor lung fibroblast cell line, WI-38, but did not show any toxicity at the highest concentration (100 μM) tested. A molecular modeling study was carried out to better understand the molecular mechanism of actions of **71** and **72** [48].

**Figure 31:**
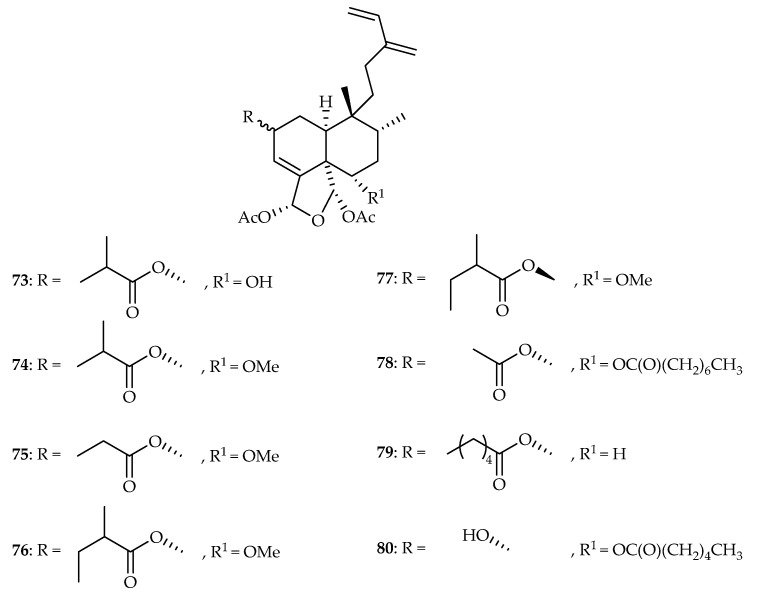
Structures of corymbulosins I, K, L, N, O, P, T, and V **73–80** [49].

The isozuelanin-type clerodane diterpenes corymbulosins I, K, L, N, O, P, T, and V **73**–**80** were isolated from the bark of *Laetia corymbulosa* (Salicaceae) (Figure 31). The absolute configurations of the newly isolated compounds were determined through electronic circular dichroism (ECD) experiments, X-ray analysis, and modified Mosher ester method. The antiproliferative activities of the diterpenes **73–80** were evaluated using A549, MDA-MB-231, MCF-7, KB, and KB-VIN cell lines. They all showed inhibitory effects on the cell proliferation of these five cancer cells, with IC_50_ values in the range of 0.45–6.39 μM, using paclitaxel as a positive control. The effects of the potent compounds corymbulosin I **73** and corymbulosin K **74** on cell cycle progression in the MDA-MB-231 cell line were further investigated by flow cytometry [49].

**Figure 32:**
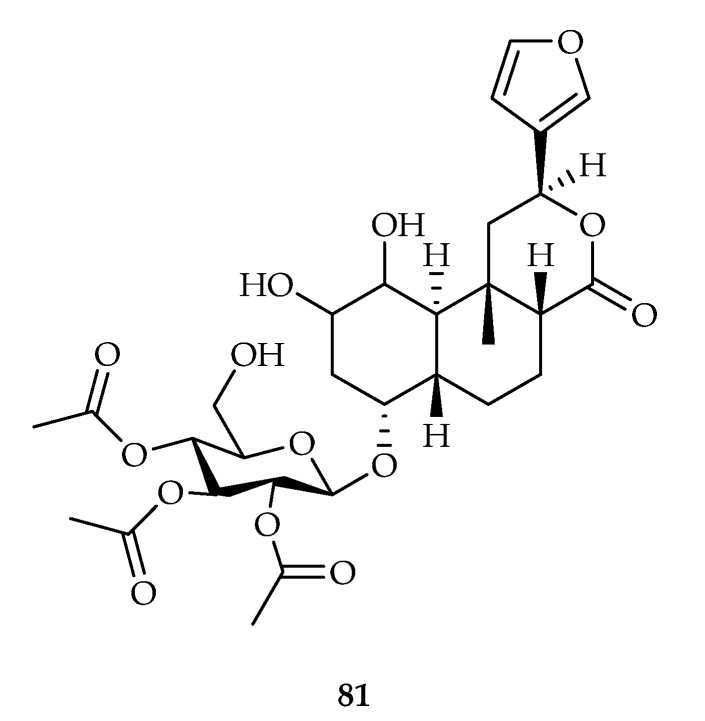
Structure of TC-2 **81** [50].

A new clerodane furano diterpene glycoside TC-2 **81** was isolated from the aqueous alcoholic extract of *Tinospora cordifolia* (Menispermaceae) stems (Figure 32). The structure of TC-2 **81** was determined on the basis of spectroscopic data interpretation and confirmed by a single-crystal X-ray crystallographic analysis of its corresponding acetate. The in vitro anti-cancer activity of TC-2 **81** was evaluated, and it showed cytotoxic activity, with an IC_50_ value of 8 μM against the HCT-116 cell line, using 5-FU as a positive control, 10.4 μM against the prostate cancer cell line (PC-3), using mitomycin-C as a positive control, and 14.8 μM against the melanoma cancer cell line (MDA-MB-435). It was demonstrated that the new clerodane diterpenoid **81** induced apoptosis of HCT-116 cells mainly by triggering ROS (reactive oxygen species) production [50].

#### 3.1.2. Halimane-Type Diterpenoids

The halimane skeleton derives from a methyl migration of the labdane skeleton and is present within a diverse number of species of the plant, animal, and bacteria kingdoms.

**Figure 33:**
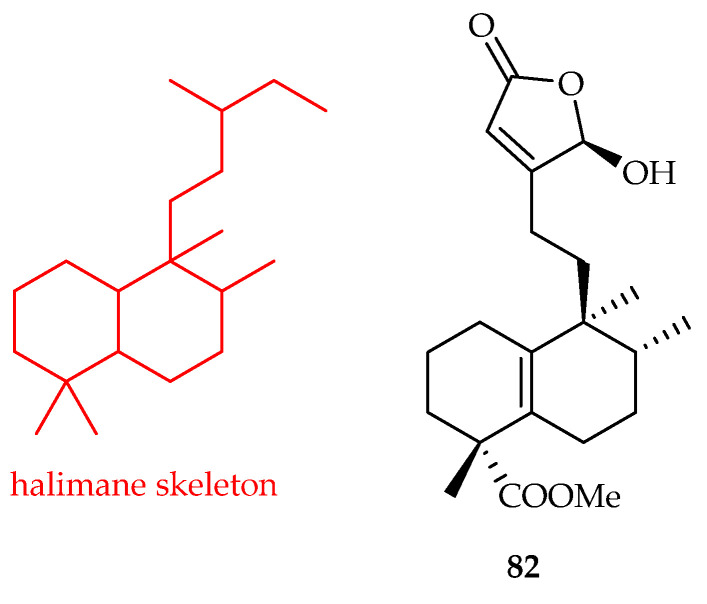
Structure of crassin H **82** [51].

Eight new diterpenoids, named crassins A–H, were isolated by 70% acetone extraction of *Croton crassifolius* Geisel (Euphorbiaceae) roots, which is primarily distributed in southern China, Laos, Thailand, and Vietnam [51]. The structures of the new compounds were determined by spectroscopic methods, and their absolute configurations were determined by electronic circular dichroism, single-crystal X-ray diffraction analysis, comparison with literature data, and biogenetic considerations. Crassin H **82** exhibited selective cytotoxicity against the A549 cells (IC_50_ 5.2 μM), compared with the HL-60 cells (IC_50_ 11.8 μM), using the MTT and SRB methods and cisplatin as the positive control (Figure 33).

#### 3.1.3. Labdane-Type Diterpenoids

**Figure 34:**
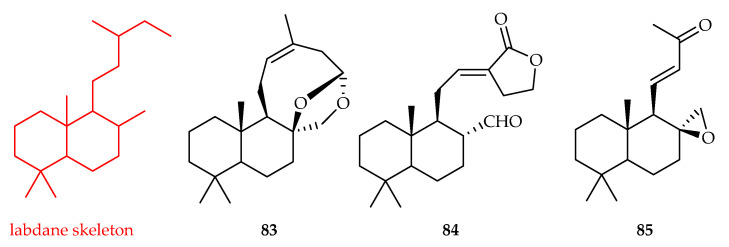
Structures of galangalditerpenes A–C **83–85** [52].

From the dried fruit of *Alpinia galanga* (Zingiberaceae), widely cultivated in China, India, and Southeast Asian countries, such as Thailand, Indonesia, and the Philippines, it was possible to isolate three new labdane-type diterpenes, named galangalditerpenes A–C **83**–**85**, by 80% aqueous acetone extraction, and their stereostructures were elucidated on the basis of their spectroscopic properties [52] (Figure 34). The melanogenesis inhibitory activities in theophylline-stimulated murine B16 melanoma 4A5 cells were evaluated, and they showed IC_50_ values of 4.4, 8.6, and 4.6 μM, respectively, better than the positive control arbutin (174 μM).

**Figure 35:**
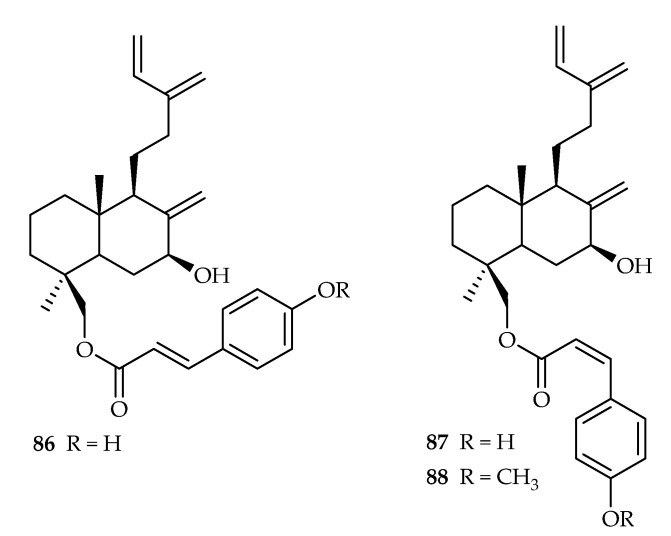
Structures of compounds **86–88** [53].

From the methanolic extract of leaves from *Araucaria bidwillii* (Araucariaceae), it was possible to isolate four new labdane diterpenes, which were unambiguously identified by applying extensive 1D and 2D NMR spectroscopic studies, as well as HR-ESI-MS [53] (Figure 35). Their relative and absolute configurations were determined using ROESY and a modified Mosher’s method, respectively. Three of the new labdane diterpenes, compounds **86**–**88**, revealed significant cytotoxic activity against the mouse lymphoma L5178Y cell line, with IC_50_ values ranging from 1.4 to 12.9 μM, using kahalalide F as a positive control.

**Figure 36:**
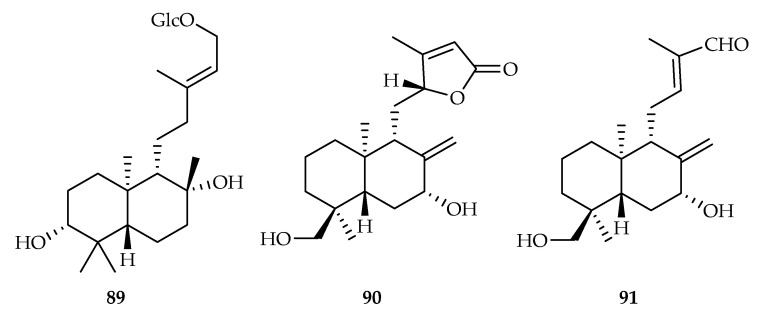
Structures of phocantoside A **89** [54], amentotaxin S **90** [55], and amentotaxin T **91** [55].

One diterpenoid glycoside labdane type, named phocantoside A **89**, was isolated from the ethanol extract of the air-dried whole plant of *Pholidota cantonensis* Rolfe (Orchidaceae) [54] (Figure 36). Its structure was determined to be (8*R*,13*E*)-*ent*-labd-13-ene-3α,8,15-triol 15-*O*-β-D-glucopyranoside by chemical and spectroscopic methods, including 1D and 2D NMR. It showed cytotoxic activity against the mouse leukemia p388D1 cancer cells, with an IC_50_ value of 17.7 μM, using taxol as the positive control.

Four *ent*-labdane-type diterpenoids (amentotaxins Q–T) were isolated by methanolic extraction and subsequent partitioning in different solvents from the leaves and twigs of *Amentotaxus argotaenia* (Taxaceae), a coniferous species endemic to China [55]. Their structures were elucidated on the basis of spectroscopic data, single-crystal X-ray diffraction, the modified Mosher’s method, and electronic circular dichroism data analyses. Amentotaxin S **90** and T **91** showed cytotoxic activity against the MDA-MB-231 cell line, with IC50 values of 1.6 and 1.5 μM, respectively, using paclitaxel as the positive control (Figure 36).

**Figure 37:**
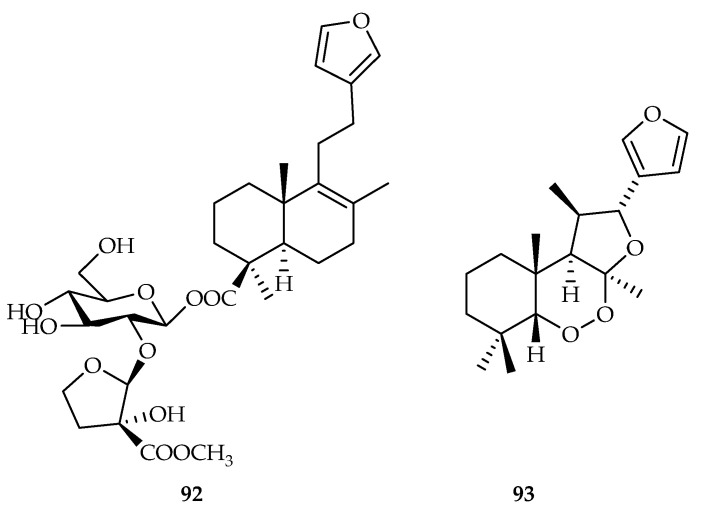
Structure of phlomoideside A **92** [56] and hedychin B **93** [57].

Phlomoidesides A–F, six unusual glycosidic labdane diterpenoids, were isolated by methanolic extraction and subsequent partitioning in different solvents, from the root of *Phlomoides betonicoides* (Diels) Kamelin and Makhm (Lamiaceae), a perennial herb present in China [56]. The structures of these compounds were determined by extensive spectroscopic analyses (including 1D NMR, 2D NMR, and HRMS), single crystal X-ray diffraction, and calculated ^13^C NMR. Only phlomoideside A **92** showed an IC_50_ value of 7.5 μM against the MCF-7 cell line, better than the positive control cisplatin (Figure 37).

Hedychin B **93** (Figure 37), an unprecedented 6,7-dinorlabdane ditepenoid with a peroxide bridge, was obtained from the rhizomes of *Hedychium forrestii* (Zingiberaceae) (China). Its structure and absolute configuration were unequivocally established by a combination of spectroscopic data and X-ray single-crystal diffractions. Hedychins B **93** was cytotoxic against the HepG2 and XWLC-05 (lung adenocarcinoma) cell lines, with IC_50_ values of 8.0 and 19.7 μM, respectively [57].

**Figure 38:**
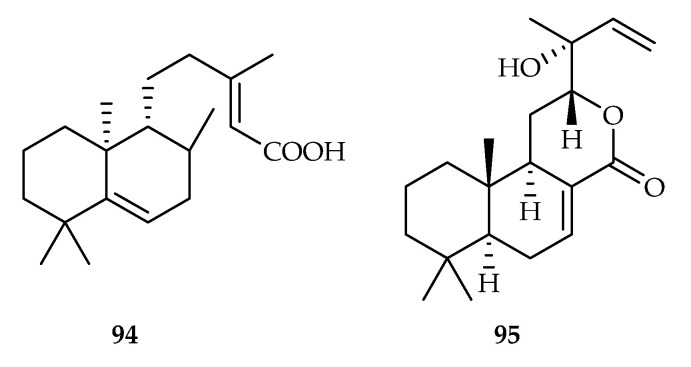
Structure of compound **94** [58] and sublyratin I **95** [59].

One novel diterpene with cytotoxic activity, *ent*-labda-5,13-dien-15-oic acid **94**, was isolated from the oleoresin obtained from the trunk of *Copaifera trapezifolia* L. (Fabaceae), a large tree present in Brazil [58]. Its structure was identified by ^1^H and ^13^C NMR, correlation 1H-1H (COSY), heteronuclear multiple quantum coherence (HMQC), heteronuclear multiple bond correlation (HMBC), and HR-ESIMS analyses. Compound **94** displayed relevant cytotoxic effect against the ACP01 (gastric cancer) and A549 cell lines, with IC_50_ values of 11.7 μM (3.57 μg mL^−1^) and 17.6 μM (5.35 μg mL^−1^), respectively, using doxorubicin as a positive control and the sulforhodamine B (SRB) assay (Figure 38).

Sublyratins A–O were isolated from the aerial parts of *Croton sublyratus* Kurz (Euphorbiaceae) by ethanolic extraction and subsequent partitioning with EtOAc [59]. Their structures were achieved by comprehensively analyzing the spectroscopic data and electronic circular dichroism spectra and using X-ray crystallographic analysis. Among them, sublyratin I **95** displayed cytotoxic activity against the HL-60 cell line, with an IC_50_ value of 2.8 μM, using the sulforhodamine B (SRB) and cell counting kit-8 (CCK-8) assays and adriamycin as the positive control (Figure 38).

**Figure 39:**
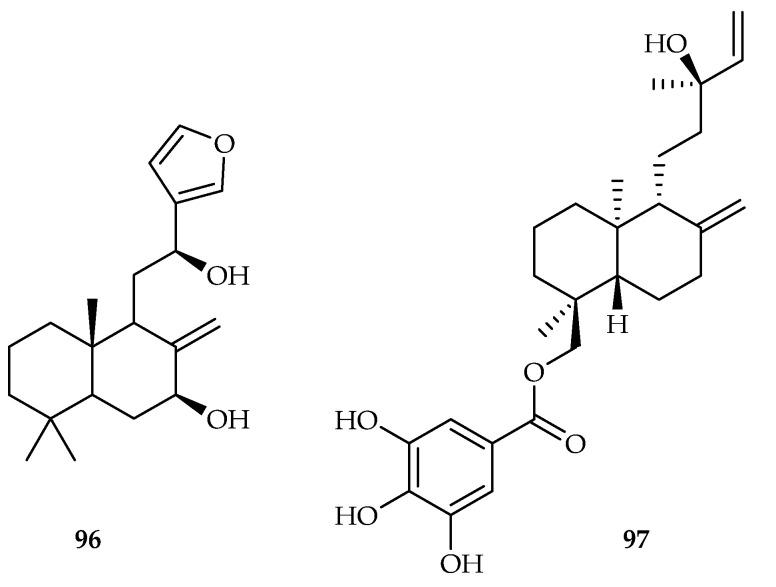
Structure of coronarin K **96** [60] and euphodane A **97** [61].

Purification of the chloroform soluble fraction from the methanol extract of *Roscoea purpurea* (Zingiberaceae) rhizomes, present in alpine grassland, grassy hillsides, and stony slopes of central to eastern Himalaya from Uttarakhand to north-east states, led to the identification of the new labdanes coronarin K and coronarin L [60]. Particularly, coronarin K **96** showed cytotoxic potential against A549 cell line, with an IC_50_ value of 13.49 µM, using the MTT assay and paclitaxel as a positive control (Figure 39).

From the roots of *Euphorbia pekinensis* Rupr. (Euphorbiaceae), by ethanolic extraction and subsequent partitioning with EtOAc, it was possible to isolate a new labdane-type diterpenoid, named euphodane A **97**, with cytotoxic activity against the U-937 (human monoblastic leukemia) cell line, with an IC_50_ value of 5.92 μM, using the cell counting kit-8 and paclitaxel as a positive control [61] (Figure 39).

**Figure 40:**
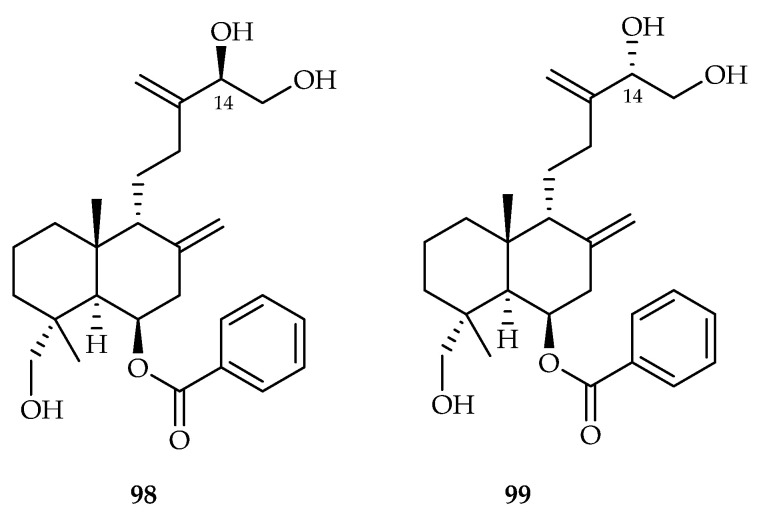
Structure of scoparicols C **98** and D **99** [62].

Two formerly undescribed labdane-type diterpenoids, scoparicols C **98** and D **99**, were separated from the aerial parts of *Scoparia dulcis* (Scrophulariaceae), a dispersed herb or subshrub now growing in most tropical areas of the world [62] (Figure 40). Extraction was performed with 95% ethanol and subsequent partitioning with EtOAc and *n*-BuOH, while structure determination was performed using spectroscopic techniques, including MS, NMR, and ECD. The two compounds are epimers at C-14, and both showed cytotoxicity against the MCF-7, MDA-MB231, and A549 cancer cell lines in the IC_50_ range of 7.63–18.2 μM, using the MTS assay and suberoylanilide hydroxamic acid (SAHA) as a positive control.

### 3.2. Tricyclic Diterpenes

#### 3.2.1. Abietane-Type Diterpenoids

**Figure 41:**
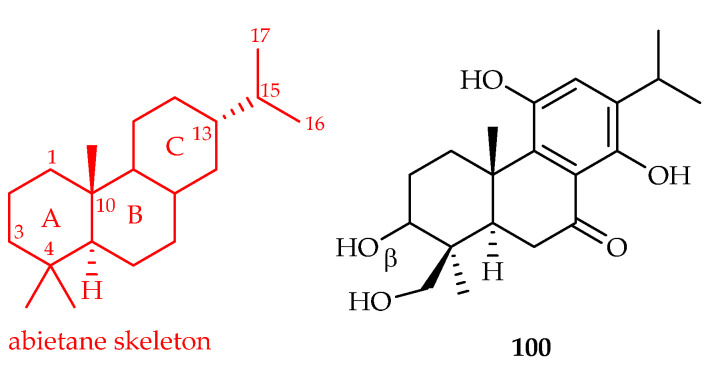
Structures of abietane skeleton and triregelin I **100** [63].

The abietane diterpenoids are characterised by a tricyclic skeleton with three fused six-membered rings and alkyl functional groups at carbons 4, 10, and 13 (Figure 41).

Triregelins A–K were isolated by methanolic extraction and subsequent partitioning with *n*-hexane, EtOAc, and *n*-butanol from the stems of *Tripterygium regelii* (Celastraceae) [63]. Their structures were determined by high-resolution electrospray ionization mass spectrometry (HR-ESI-MS), extensive NMR spectroscopic analysis, and comparison of their experimental CD spectra with the calculated ECD spectra. Triregelin I **100** showed significant cytotoxicity against the A2780 (human ovarian) and HepG2 cell lines, with IC_50_ values of 5.88 and 11.74 μM, respectively, using taxol as the positive control (Figure 41).

**Figure 42:**
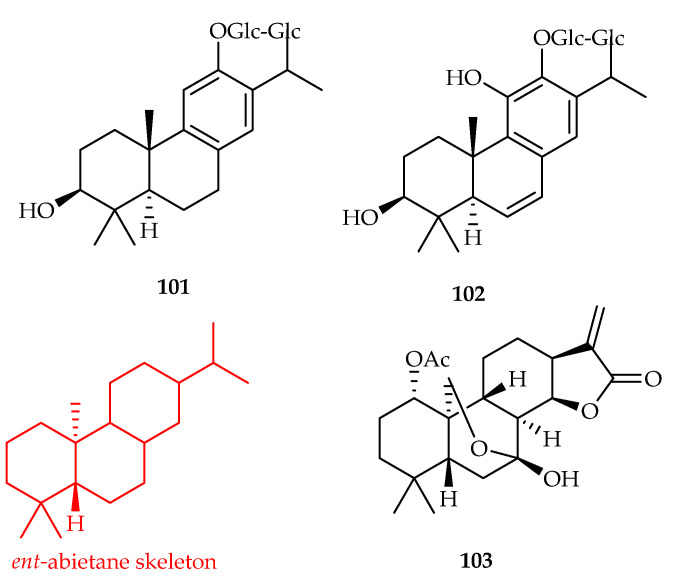
Structures of perovskiaditerpenoside A **101** [64], perovskiaditerpenosides C **102** [64], and serrin K **103** [65].

Perovskiaditerpenosides A–D were isolated from the BuOH extract of the whole plant of *Perovskia atriplicifolia* (Labiatae) [64]. Their structures were determined by chemical methods and comprehensive spectroscopic analyses, including MS, IR, and NMR (1D and 2D). Perovskiaditerpenoside A **101** was cytotoxic against the HepG2, NB4 (human acute promyelocytic leukemia cell), HeLa, MCF-7, and HL-60 cell lines, with IC_50_ values in the range of 0.23–8.72 μM, while perovskiaditerpenoside C **102** had significant cytotoxic activity against the NB4, HeLa, K562, MCF-7, and HL-60 cell lines, with IC_50_ values in the range of 0.35–5.17 μM, using cisplatin as the positive control (Figure 42).

Four new *ent*-abietane diterpenoids were isolated by extraction with 70% aqueous acetone from the aerial parts of *Isodon serra* (Maxim.) Hara (Lamiaceae), a periennal herb used as a Chinese folk medicine for the treatment of jaundice hepatitis, acute cholecystitis, and enteritis [65]. Their structures were elucidated by extensive spectroscopic analysis. Among them, compound **103**, named serrin K, showed cytotoxicity towards the HL-60, SMMC-7721, A549, MCF-7, and SW480 cell lines, with IC_50_ values ranging from 9.4 to 20.4 μM. The IC_50_ value was calculated by Reed and Muench’s method, using cisplatin as positive control (Figure 42).

**Figure 43:**
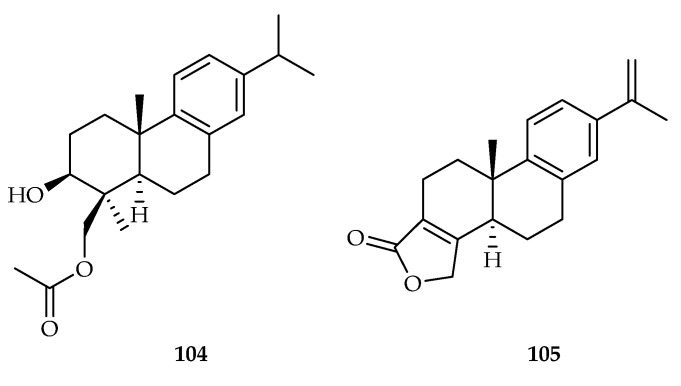
Structures of nepetaefolin F **104** [66] and nepetaefolin G **105** [66].

Nepetaefolins A–J were isolated from 95% EtOH extraction of the whole plant of *Caryopteris nepetaefolia* (Benth.) Maxim. (Verbenaceae), a folk medicine used to treat inflammatory diseases in eastern China [66]. Their structure was determined by extensive spectroscopic analysis. They were tested against the LUPF045 (non-small-cell lung), LUPF003 (non-small-cell lung), OVPF038 (ovarian cancer), and OVPF008 (ovarian cancer) cell lines; among them, compounds **104** and **105**, namely nepetaefolin F and nepetaefolin G, showed higher cytotoxicity than paclitaxel (IC_50_ 11.2 μM) in one non-small-cell lung cancer (LUPF045) patient-derived xenograft (PDX) model, when tested using PDX models and the adenosine triphosphate-tumor chemosensitivity assay (ATP-TCA) (Figure 43).

**Figure 44:**
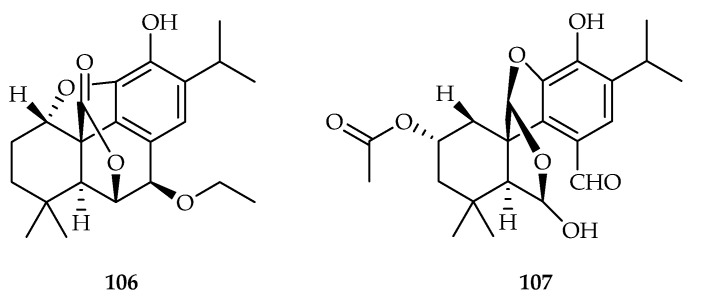
Structures of compounds **106** and **107** [67].

Four new diterpenoids, named plebeins C–F, were isolated from ethanolic extraction of the whole plant of *Salvia plebeian* R. Br. (Lamiaceae), an annual or biennial herb, which has been used in folk medicine for the treatment of cough, hepatitis, and haemorrhoids [67]. Compounds **106** and **107** showed anti-proliferative activity against the CaCo2 (human colorectal carcinoma) cell line, with IC_50_ values of 9.65 μM for Compound **106** and 17.86 μM for Compound **107**. Adriamycin was used as the positive control (Figure 44).

**Figure 45:**
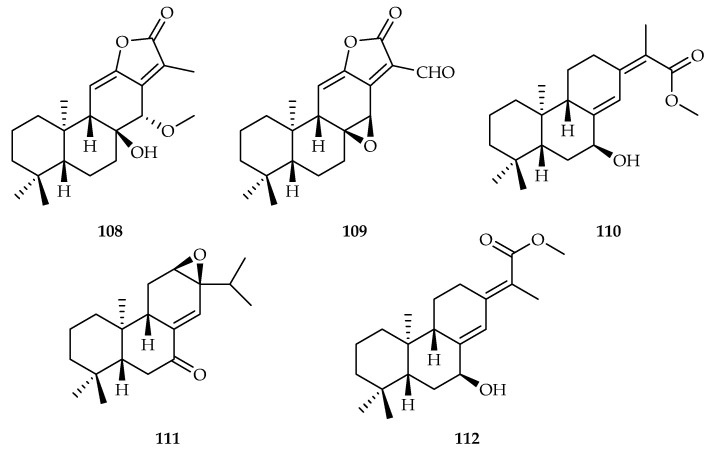
Structures of euphonoids A–D **108**–**111** [68] and fischeriabietane A **112** [68,69].

Four new *ent*-abietanes, euphonoids A–D **108**–**111**, were isolated from the roots of *Euphorbia fischeriana* (Euphorbiaceae), a perennial herb mainly distributed in northeastern China [68]. Anyway, the structure of a previously reported *ent*-abietane diterpenoid, fischeriabietane A **112** [69], was revised. The new compounds and revised one showed an IC_50_ value < 20 μM against the C4-2B and C4-2B/ENZR (enzalutamide-resistant C4-2B cells) cell lines, while compound **108** showed cytotoxicity also against the HCT-15 and RKO cell lines, with compound **112** against the HCT-15 cell line. Doxorubicin was used as the positive control. Biological activity was ascribed to the presence of the α,β-unsaturated carbonyl moiety (Figure 45).

**Figure 46:**
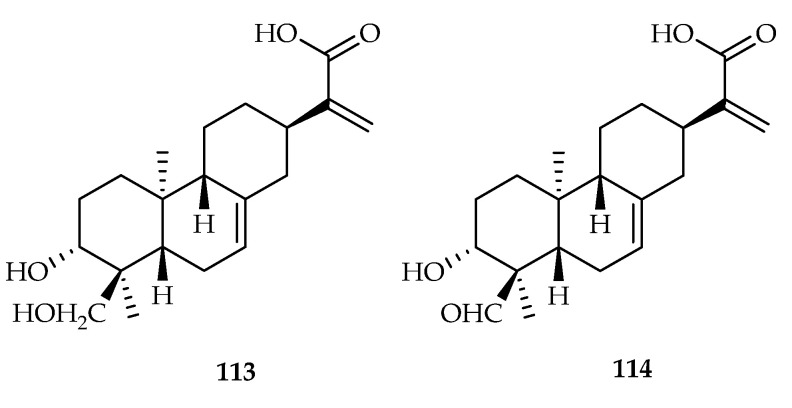
Structures of fischerianoids A **113** and B **114** [70].

Fischerianoids A–C were also isolated and identified from the ethyl acetate extracts of roots of *Euphorbia fischeriana* (Euphorbiaceae). Fischerianoid A **113**, and fischerianoid B **114** showed selective cytotoxicity against the MM-231 (breast cancer) and Hep3B cell lines, with IC_50_ values of 12.10 and 15.95 μM for compound **113**, respectively, with 9.12 and 8.50 μM, respectively, for compound **114**. Cisplatin was used as the positive control, and no obvious cytotoxicity was observed for the normal cell NCM460 [70] (Figure 46).

**Figure 47:**
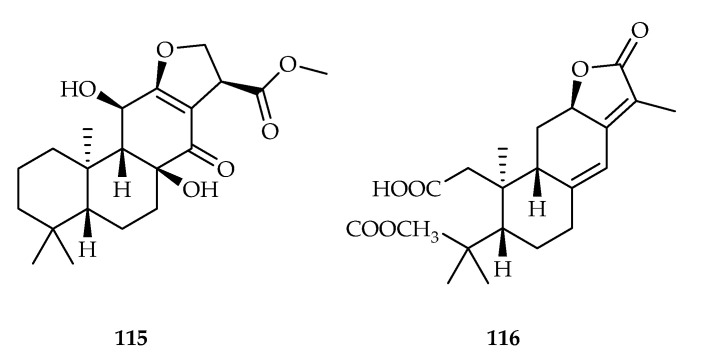
Structures of difischenoid A **115** and B **116** [71].

Moreover, difischenoids A–D and their structures were also isolated from the roots of *Euphorbia fischeriana*, as determined via extensive spectroscopic data, while the absolute configurations were elucidated by means of single-crystal X-ray diffraction analysis, Rh_2_(OCOCF_3_)_4_ induced CD spectrum, and ECD calculations [71]. Difischenoid A **115** was only active against HeLa cells, with an IC_50_ value of 15.47 μM, while difischenoid B **116** was more potent than cisplatin against the HeLa and MCF-7 cells, with IC_50_ values of 3.75 and 9.31 μM, respectively, using the MTT assay. Difischenoid B **116** shows a modified abietane skeleton, probably due to a cycloreversion reaction of ring A, which formed a *seco*-diterpenoid skeleton (Figure 47).

**Figure 48:**
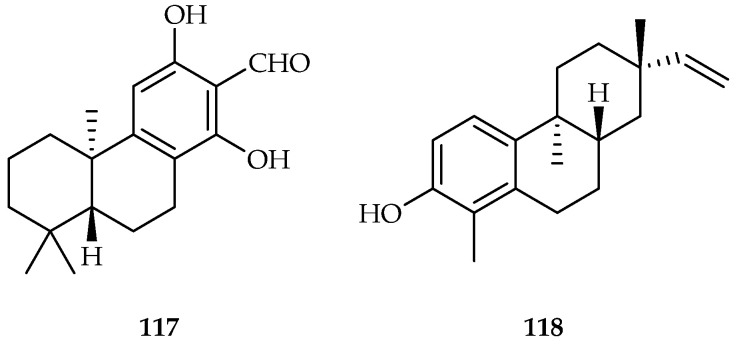
Structures of euphorfischerin A **117** [72] and euphorfischerin B **118** [72].

Finally, euphorfischerins A–E were isolated from the roots of *Euphorbia fischeriana*, as well, and their chemical structures and absolute configurations were determined by the interpretation of NMR, HR-ESI-MS, ECD, and X-ray diffraction data [72]. Euphorfischerin C-E had an abietane skeleton, while the only two active compounds, euphorfischerin A **117** and euphorfischerin B **118**, have C_18_ and C_19_ skeletons, respectively. Euphorfischerin A **117** showed cytotoxicity against the HeLa, H460 (non-small-cell lung cancer), and Namalwa (Burkitt lymphoma) cell lines, with IC_50_ values of 4.6, 11.5, and 16.4 μM, respectively, while euphorfischerin B **118** provided comparable IC_50_ values of 9.5, 17.4, and 13.3 μM against the three cancer cell lines, respectively, using the cell counting kit-8 assay and doxorubicin as a positive control (Figure 48).

**Figure 49:**
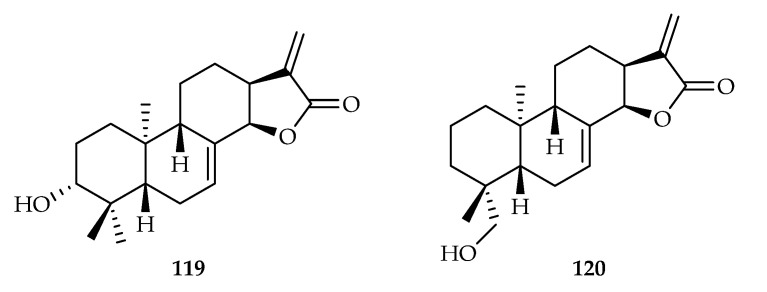
Structures of isoforrethins C **119** and D **120** [73].

Isoforrethins A–D were isolated from the aerial parts of *Isodon forrestii* var. *forrestii* (Lamiaceae), a perennial herb widely distributed at altitudes of 2650–3300 m in northwest of Yunnan Province and southwest of Sichuan Province, People’s Republic of China [73]. Their structures were elucidated on the basis of spectroscopic data interpretation, single crystal X-ray diffraction, and quantum chemical calculation. Isoforrethin C **119** showed equivalent cytotoxic activity with cisplatin to the SW480 (human colon cancer) cell line, while isoforrethins D **120** exhibited significant cytotoxic activity against SMMC-7721, A549, MCF-7, and SW480 (Figure 49).

**Figure 50:**
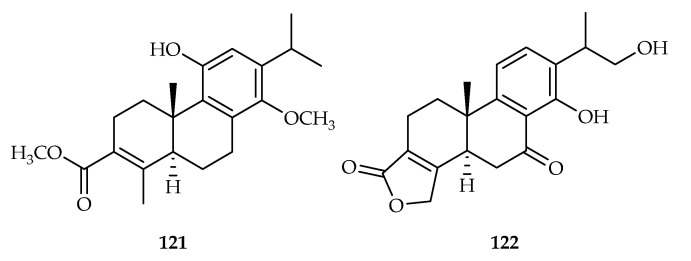
Structures of tripterregeline A **121** [74] and trigohowimine A **122** [75].

Tripterregeline A **121** was isolated from the roots of *Tripterygium regelii* (Celastraceae), which is naturally distributed throughout East Asia. Tripterregeline A **121** was evaluated for its cytotoxicity against the HL-60, SMMC-7721, A549, MCF-7, and SW480 cell lines and showed stronger inhibitory activities than those of cisplatin, with IC_50_ values ranging from 0.58 to 5.82 μM [74] (Figure 50).

Trigohowimine A **122** was isolated from the stems and leaves of *Trigonostemon howii* (Euphorbiaceae), which is widely distributed in the tropical and subtropical regions of Asia. Trigohowimine A **122** showed significant cell growth inhibitory activities against the HL-60, SMMC-7721, A54, MCF-7, and SW480 cell lines, with IC_50_ values of 0.82, 2.65, 5.64, 6.26, and 8.53 μM, respectively, better than the positive control cisplatin [75] (Figure 50).

**Figure 51:**
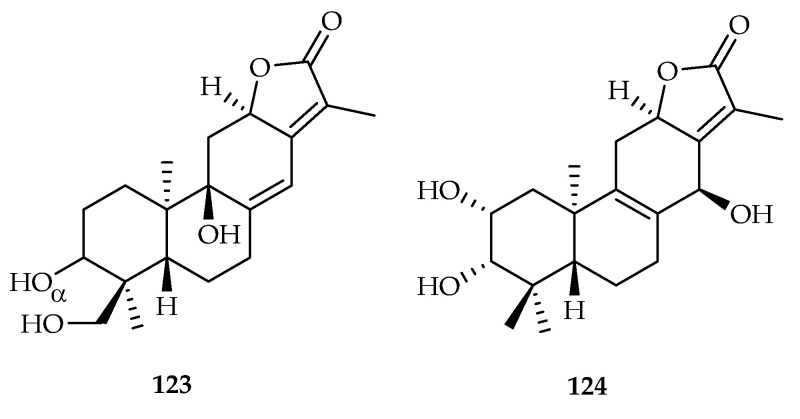
Structures of euphelionolides F **123** [76] e L **124** [76].

A phytochemical investigation on the aerial part of *Euphorbia helioscopia* Linn. (Euphorbiaceae) led to the isolation of 14 highly oxygenated new *ent*-abietane diterpenoids, euphelionolides A–N [76]. The cytotoxicities of the isolates were evaluated in vitro against MCF-7 and PANC-1 cell lines via the MTT method. Paclitaxel and gemcitabine were used as positive controls. The results showed that only euphelionolides F **123** and L **124** had significant cytotoxic activity against the MCF-7 (IC_50_ 9.5 and 9.8 μM, respectively) and PANC-1 (IC_50_ 10.7 and 10.3 μM, respectively) cells. However, compounds analogues of **123** and **124** did not exhibit significant cytotoxic effects; the structure–activity relations of the *Euphorbia* diterpenoids results are still unclear (Figure 51).

**Figure 52:**
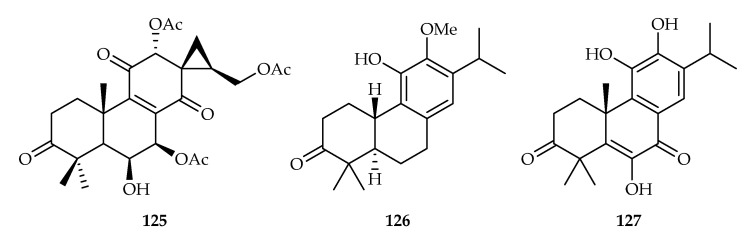
Structure of spiroscutelone B **125** [77], cuceolatin D **126** [78], and metaglyptin A **127** [79].

Three new abietane-type diterpenoids possessing a spiro-cyclopropane moiety, named spiroscutelones A–C, were isolated from the leaves of *Plectranthus scutellarioides* (Lamiaceae), which is distributed in China, India, Indonesia, the Philippines, and Australia. Spiroscutelone B **125** exhibited the most potent cytotoxicity against the MCF-7 cell line, with an IC_50_ value of 17.9 μM, while it had low cytotoxicity against the normal cell line (WI-38) [77] (Figure 52).

One new abietane-type diterpene was isolated from the leaves of *Cunninghamia lanceolata* (LAMB.) HOOK. (Taxodiaceae), an evergreen arbor mainly distributed in the regions south to the Yangtze River of China [78]. The new compound **126**, named cuceolatin D, showed significant cytotoxicity against the MDA-MB-231, MCF-7, and HeLa cell lines, with IC50 values all below 5 μM, using the MTT method and doxorubicin as a positive control (Figure 52).

From the stem bark of *Metasequoia glyptostroboides* (Taxodiaceae), thirteen previously undescribed diterpenoids (metaglyptins A–M) were isolated by ethanolic extraction, and their cytotoxicity was evaluated against the HeLa, AGS (gastric cancer), and MDA-MB-231 cancer cell lines using the MMT assay [79]. Only metaglyptin A **127** exhibited moderate cytotoxicity against the MDA-MB-231 cell line, with an IC_50_ value of 20.02 μM, using cisplatin as a positive control (Figure 52).

**Figure 53:**
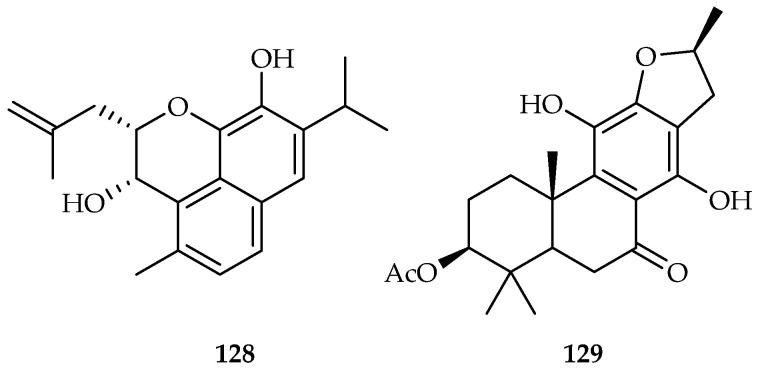
Structures of ceratodiol **128** [80] and 3-acetoxylteuvincenone G **129** [81].

Two previously undescribed rearranged abietanes, named ceratol and ceratodiol **128**, were isolated by extraction with CH_2_Cl_2_ from the roots of *Salvia ceratophylla* (Lamiaceae), a biennial lemon-scented herb that grows widely in Iran, Anatolia, Afghanistan, and the northern parts of Iraq and Transcaucasia [80] (Figure 53). The structures of the unknown compounds were defined based on the analyses of their spectroscopic data, including 1D and 2D NMR and high-resolution electrospray mass spectra. Their cytotoxicity was evaluated against MOLT-4 (human lymphoblastic leukemia) and MCF-7 using the MTT assay. Ceratodiol **128** showed an IC_50_ value of 7.9 against MOLT-4, using cisplatin as a positive control.

A new abietane diterpenoid, named 3-acetoxylteuvincenone G **129**, was isolated by ethanolic extraction and subsequent partitioning with EtOAc and *n*-BuOH from the whole plant of *Ajuga ovalifolia* var. *calantha* (Lamiaceae), an endemic medicinal plant in the west region of China [81] (Figure 53). The structure of the new compound was elucidated by means of extensive spectroscopic analyses. Compound **129** suppressed the A549 cell proliferation, with an IC_50_ value of 10.79 μM, and induced cell apoptosis through the SHP2/ERK1/2 and SHP2/AKT pathways.

**Figure 54:**
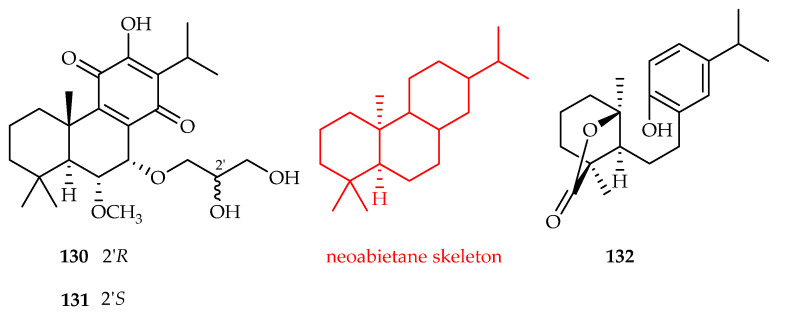
Structures of salvidesertone E **130** [82], salvidesertone F **131** [82], and kunminolide A **132** [83].

Ten new abietane quinone diterpenoids were isolated by ethanolic extraction and subsequent partitioning with petroleum ether from the roots extract of *Salvia deserta* Schang (Lamiaceae), a perennial plant mainly distributed in the northern Xinjiang, China [82]. Their chemical structures were delineated by extensive spectrometric and spectroscopic techniques, including HRESIMS, NMR, UV, IR, and single-crystal X-ray diffraction analysis, and calculated ^13^C NMRDP4+ analysis, calculated ECD, and Mo_2_(OAc)_4_-induced ECD. They were evaluated for their antiproliferative activities against the A-549, SMMC-7721, SW480, MCF-7, and HL-60 cancer cell lines and a normal epithelial cell line, BEAS-2B, in vitro. Salvidesertone E **130** and salvidesertone F **131** showed more potent antiproliferative effect against A549 than the positive control cisplatin, with IC_50_ values of 5.89 and 6.94 μM, respectively, while IC_50_ values of >40 and 35.48 μM were reported for normal epithelial cell line BEAS-2B, respectively (Figure 54).

Kunminolide A **132** is a novel abietane-like diterpene, designated as 9,10-*seco*-*neo*-abietane, isolated from the leaves of *Isodon interruptus* (Lamiaceae), a medicinal plant abundant in central Kunming, Yunnan Province, P.R. China, together with the new *ent*-kaurene diterpene rabdokunmin F (see Section 3.3.4) [83]. Their structures were determined by spectroscopic means, including the analysis of 1D and 2D NMR spectral data and X-ray analysis of **132** confirmed the chemical structure. Kunminolide A **132** showed cytotoxic activity, with IC_50_ values of <20 μM against KB and MCF7 cell lines, using paclitaxel as a positive control (Figure 54).

**Figure 55:**
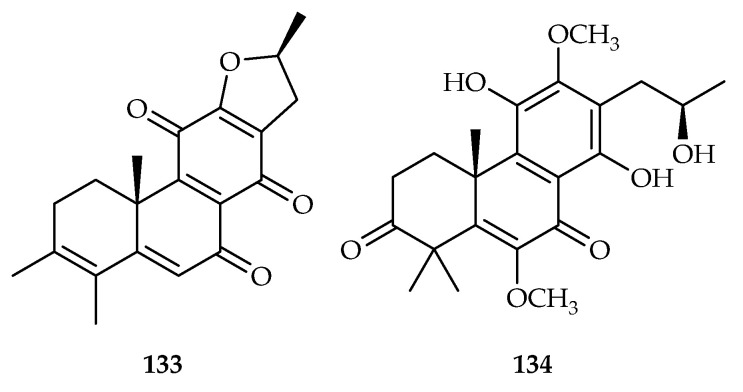
Structures of compounds **133** and **134** [84].

Two new abietane diterpenoids, **133** and **134**, were first isolated by ethanolic extraction and subsequent partitioning with EtOAc and *n*-BuOH from the stems of *Clerodendrum bracteatum* (Lamiaceae) [84]. Their structures were established by extensive analysis of mass spectrometric and 1D and 2D NMR spectroscopic data; additionally, the in vitro cytotoxic activities were evaluated against the HL-60 and A549 cell lines by the MTT method, using cisplatin as a positive control. Compounds **133** and **134** demonstrated cytotoxic activities against the HL-60 (IC_50_ 21.22 and 10.91 μM) and A549 (IC_50_ 13.71 and 18.42 μM) cell lines, respectively (Figure 55).

**Figure 56:**
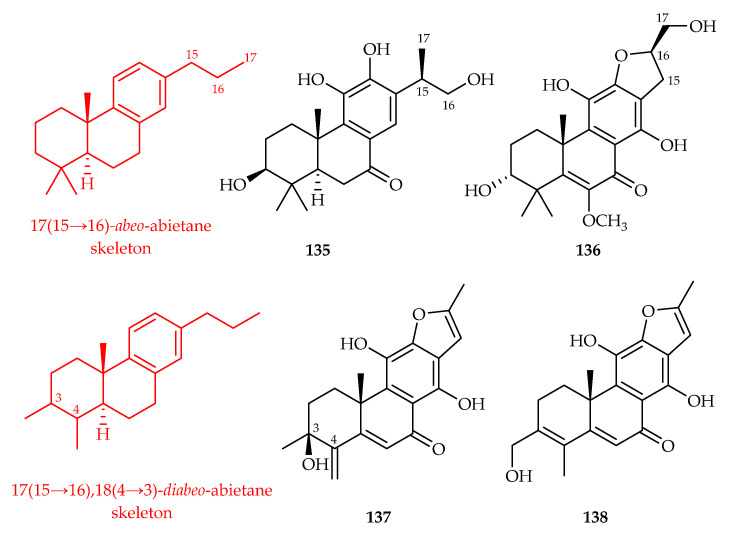
Structures of clerodenoid A **135**, clerodenoid D **136**, clerodenoid E **137**, and clerodenoid F **138** [85].

Six new C-ring aromatised abietane diterpenoids, clerodenoids A–F, were isolated by ethanolic extraction and subsequent partitioning with EtOAc from the roots of *Clerodendrum chinense* var. simplex (Verbenaceae), an ethnomedicinal plant of the Dai minority in China [85]. Their structures were elucidated by extensive spectroscopic data, X-ray crystallography and ECD calculation. Among the new compounds, clerodenoids A **135** and D **136** showed IC_50_ values of 10.75 and 10.58 μM against HL-60 cancer cell lines, respectively, while clerodenoids E **137** and F **138** showed a strong antiproliferative activity against HL-60 and A549, with IC_50_ values ranging from 1.00 to 5.03 μM, using the sulforhodamine B (SRB) assay and adriamycin as a positive control. Compound **136** belongs to the rearranged abietane skeletons of 17 (15 → 16)-*abeo*-abietane, while compounds **137** and **138** belong to the 17 (15 → 16),18 (4 → 3)-*diabeo*-abietane (Figure 56).

**Figure 57:**
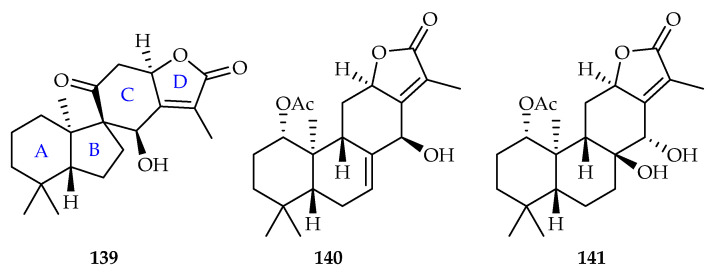
Structures of phorneroids A-C **139–141** [86].

From the aerial parts of *Euphorbia neriifolia* (Euphorbiaceae), a poisonous plant mainly distributed in tropical and subtropical regions of Asia, it was possible to isolate thirteen undescribed diterpenoids by ethanolic extraction and subsequent partitioning with EtOAc [86]. Four of the new compounds showed cytotoxic activity in the IC_50_ range values of 2.5–9.9 μM against the A549 and HL-60 cancer cells, using the sulforhodamine B (SRB) and cell counting kit-8 (CCK-8) methods and adriamycin as the positive control. Compounds **139**, **140**, and **141**, named phorneroids A-C, are *ent*-abietane, while phorneroid H is an *ent*-atisane-type (see Section 3.3.1). Phorneroid A **139** represents the first example of an 8-spiro-fused 9,10-*seco*-*ent*-abietane diterpenoid lactone featuring a unique 6/5/6/5 spirocyclic framework. The structures and absolute configurations of all the undescribed compounds were unambiguously determined, based on the comprehensive analyses of their NMR, MS, ECD, and X-ray crystallography data (Figure 57).

**Figure 58:**
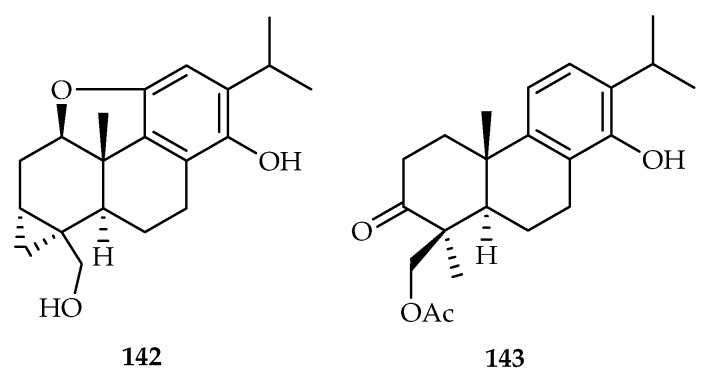
Structures of hypoglicin R **142** [87] and 18-acetyl-triptobenzene A **143** [87].

From *Tripterygium hypoglaucum* (H. L’ev.) Hutch. (Celastraceae), a plant present in China, it was possible to isolate two new compounds with cytotoxic activity against the SW1990 (human pancreatic cancer) cell line [87]. Compounds **142** and **143**, named hypoglicin R and 18-acetyl-triptobenzene A, respectively, were obtained after extraction with 90% EtOH of *T. hypoglaucum* stem and subsequent partitioning with EtOAc. Their structures were elucidated by HRESIMS, 1D NMR, and 2D NMR spectroscopic analysis. The IC_50_ values for **142** and **143** were 19.28 and 9.91 μM, respectively, using the MTT assay and paclitaxel as a positive control (Figure 58).

**Figure 59:**
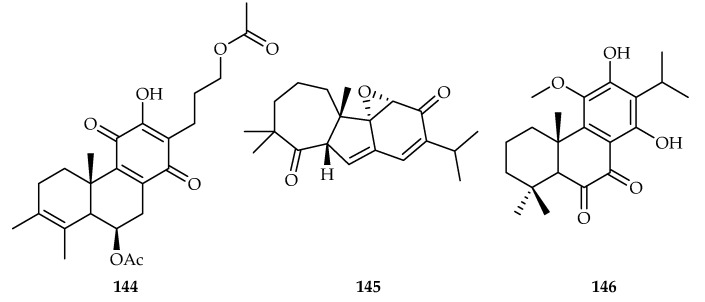
Structures of 6-acetylfredericone B **144** [88], salviyunnanone A **145** [89], and complanatin D **146** [90].

Chemical investigation of the dichloromethane extract of the aerial parts of *Plectranthus scutellarioides* (Lamiaceae) led to the isolation and characterization of the abietane diterpene 6-acetylfredericone B **144** (Figure 59). Compound **144** exhibited a cytotoxic effect, with an IC_50_ value of 17.6 μM against MM-CSCs (human multiple myeloma cancer stem) cells, using bortezomib as a positive control [88].

Salviyunnanone A **145** (Figure 59), a cytotoxic diterpenoid possessing an unprecedented 7/5/6/3 ring system derived from normal abietane skeleton, was characterised from *Salvia yunnanensis* (Lamiaceae) roots. A plausible biosynthetic pathway for salviyunnanone A **145** was also described. Compound **145** showed significant toxicity against the HL-60, SMMC-7721, A549, MCF-7, and SW480 cell lines, with IC_50_ values of 2.63, 2.52, 4.84, 1.18, and 3.23 μM, respectively, using cisplatin as a positive control [89].

Complanatin D **146** (Figure 59) was isolated from the club moss *Lycopodium complanatum* (Lycopodiaceae) whole herbs. A plausible biogenetic pathway of abietane diterpenoid **146** was also proposed. Compound **146** inhibited the MSTO-211H cell line (human lung cancer), with an IC_50_ value of 17.34 μM, using staurosporine as a positive control [90].

**Figure 60:**
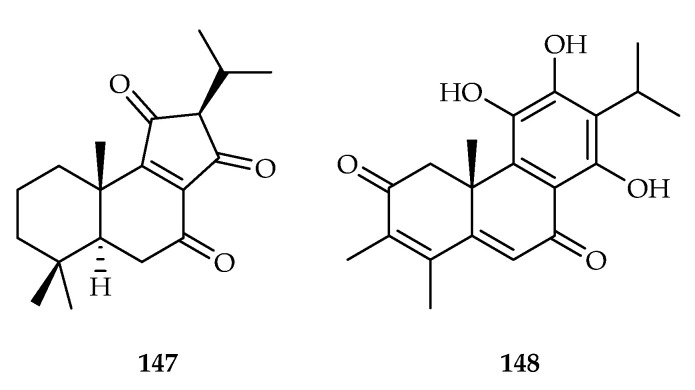
Structure of leriifolione 1**47** [91] and salvimulticanol **148** [92].

Phytochemical investigation of the lipophilic extract of the roots of *Salvia leriifolia* (Lamiaceae) resulted in the isolation of leriifolione **147**, an unprecedented nor-abietane isoprenoid possessing a rare five-membered C-ring (Figure 60). A putative pathway for its biosynthesis was proposed. The cytotoxicity of leriifolione **147** against the rat myoblast L6 cells showed an IC_50_ value of 2.6 μM, using podophyllotoxin as a positive control [91].

Salvimulticanol **148** was isolated from the aerial parts of *Salvia multicaulis* (Labiatae) (Figure 60). Structure–activity relationships and a biosynthetic pathway for this aromatic abietane diterpenoid were proposed. Compound **148** was screened against a panel of human drug sensitive and multidrug-resistant cancer lines. Salvimulticanol **148** showed cytotoxicity, with IC_50_ values of 15.32 and 8.36 μM, towards the CCRF-CEM (drug-sensitive leukemia) and CEM/ADR5000 (its multidrug resistant P-glycoprotein-overexpressing subline) cell lines (treated once per week with 5000 ng/mL doxorubicin), respectively [92].

#### 3.2.2. Cassane-Type Diterpenoids

**Scheme 7:**
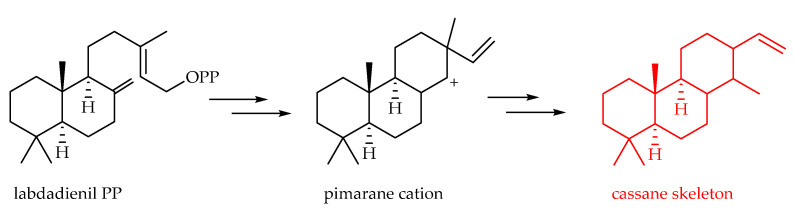
Formation of cassane skeleton.

Cassane diterpenoids have been isolated in recent years from different species of medicinal plants belonging to the *Caesalpinia* genus, and their skeleton seems to derive from the pimarane skeleton, due to a migration of the methyl group [93] (Scheme 7).

**Figure 61:**
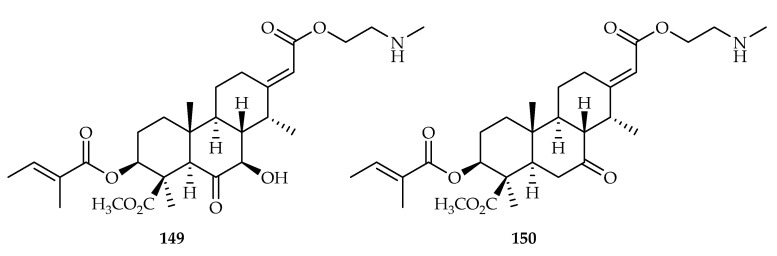
Structures of erythroformine A **149** and B **150** [94].

The two cassaine diterpenoid amines erythroformine A **149** and B **150** were isolated from the bark of *Erythrophleum fordii* Oliver (Fabaceae), a species with both medicinal and poisonous properties, which is widely distributed throughout China, Vietnam, and Taiwan (Figure 61) [94]. Their cytotoxic activity was examined in vitro against the A549, NCI-H1975 (lung adenocarcinoma), and NCI-H1229 (lung large cell carcinoma) cell lines, using the MTT assay and camptothecin as a positive control. Erythroformine A **149** and B **150** showed potent cytotoxicity against all three cell lines, with IC_50_ values in the range of 0.4–1.4 μM, even better than camptothecin.

**Figure 62:**
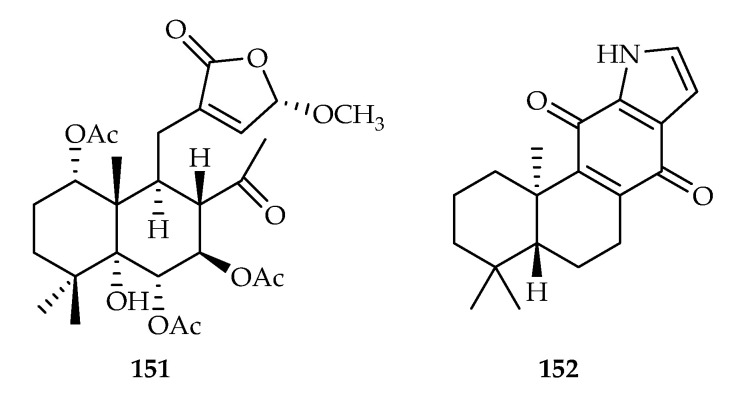
Structures of caesappine A **151** [95] and euphkanoids H **152** [96].

From *Caesalpinia sappan* seeds, caesappine A **151** was isolated by methanolic extraction and subsequent partitioning with petroleum ether, CHCl_3_, EtOAc, and *n*-BuOH [95]. Its structure was elucidated by analysing its 1D NMR, 2D NMR, and HR-ESI-MS spectra. Compound **151** showed cytotoxic activity against HeLa cells, with an IC_50_ value of 11.42 μM, using the MTT method and cisplatin as a positive control (Figure 62).

Euphkanoids H **152**, the first example of C-17 norcassane indole-diterpene, was isolated from the roots of *Euphorbia fischeriana* by ethanolic extraction and subsequent partitioning with EtOAc [96]. Its structure was identified by spectral methods, and the absolute configuration was determined by ECD calculation and single crystal X-ray diffraction. Compound **152** showed significant cytotoxicity against HEL (human erythroleukemia) cells, with an IC_50_ value of 3.2 μM, using the MTT assay and doxorubicin as a positive control. A simple mechanistic study revealed that **152** could induce cell cycle arrest at G0/G1 phase and apoptosis in HEL cells (Figure 62).

#### 3.2.3. Dolabrane-Type Diterpenoids

**Figure 63:**
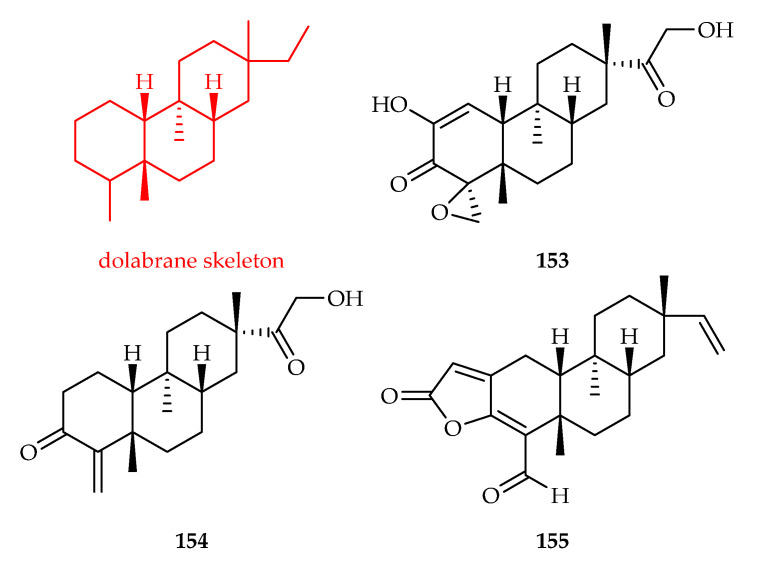
Structures of dolabrane skeleton, tagalene I **153** [97,99] tagalene K **154** [99], and tagalide A **155** [100].

Tagalenes G–I [97] and tagalenes J–K [98] (Figure 63) were isolated from the stems and twigs of Mangrove plant *Ceriops tagal*, which is the sole species of the genus *Ceriops* (Rhizophoraceae) present in China, mainly in Hainan Island. The absolute configuration of tagalene I **153** was established by single-crystal X-ray diffraction analyses by Zhang and Coll [99]. Tagalene I **153** displayed potent cytotoxic effects against the MDA-MB-453, MDA-MB-231, SK-BR-3 (human breast cancer), and MT-1 (human contaminated breast cancer) cell lines, with IC_50_ values of 8.97, 8.97, 4.62, and 3.93 μM, respectively, whereas tagalene K **154** (named tagalon D by Zhang and Coll) exhibited selective cytotoxicity against MT-1 cell line, with an IC_50_ value of 8.07 μM [99].

Tagalide A **155**, the first C_22_ skeletal dolabrane, with an unprecedented 5/6/6/6-fused tetracyclic core, was also isolated from the Chinese mangrove *Ceriops tagal* (Rhizophoraceae) stems and twigs (Figure 63). A biosynthetic pathway of tagalide A **155** was proposed. Tagalide A **155** exhibited potent cytotoxicities against: MDA-MB-453, MDA-MB-231, SK-BR-3, MCF-7, MT-1, and ZR-75-1 (epithelial from the mammary gland) cell lines, with IC_50_ values of 1.73, 8.12, 2.45, 12.03, 3.75, and 1.97 μM, respectively. Cisplatin was used as the positive control. Investigation of the mechanisms on MD-MBA-453 cells revealed that tagalide A **155** induces ROS-mediated apoptosis and cell-cycle arrest at the G2/M phase [100].

#### 3.2.4. Icetexane-Type Diterpenoids

**Figure 64:**
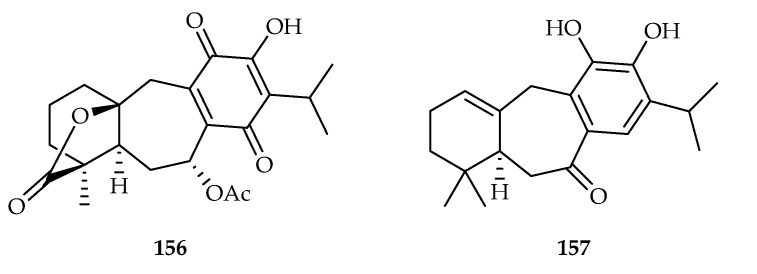
Structure of compound **156** [101] and salviadenone A **157** [102].

From the aerial parts of *Salvia ballotiflora* (Lamiaceae), a new icetexane, derivative **156**, was isolated (Figure 64). The relative configuration of **156** was established with the aid of a NOESY spectrum, while vibrational circular dichroism (VCD) allowed for the establishment of the absolute configuration. The natural product **156** showed cytotoxicity against U-251 (human glioblastoma) and SKLU-1 (human lung adenocarcinoma), with IC_50_ values of 1.4 and 0.82 μM, respectively, while it was non-toxic to FGH (healthy gingival human fibroblasts). Adriamycin at 0.5 μM was used as the positive control [101].

Four new icetexane diterpenoids, salviadenones A–D, together with two highly modified spirocyclic diterpenoids (see Section 5.3), were isolated from the roots of *Salvia deserta (Lamiaceae)* by ethanolic extraction and subsequent partitioning with petroleum ether [102]. Their structures were elucidated by spectroscopic analysis, quantum chemical calculations (including the ^13^C chemical shift calculations with DP4+ probability analysis and ECD calculations), and single-crystal X-ray diffraction analysis. Only salviadenone A **157** showed moderate cytotoxicity against the HL-60 cell line, with an IC_50_ value of 17.70 μM, using the MTS method and cisplatin as a positive control.

#### 3.2.5. Pimarane-Type Diterpenoids

**Figure 65:**
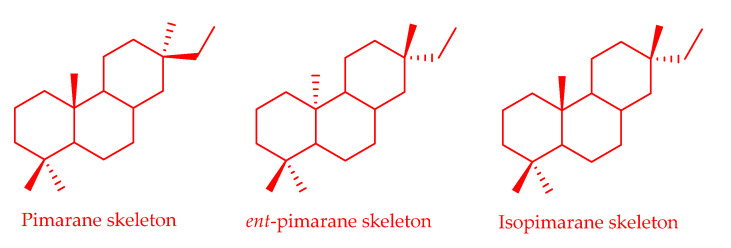
Skeleton of pimarane type.

The skeletons of pimarane, *ent*-pimarane, and isopimarane are reported in Figure 65.

Tagalon A, an isopimarane diterpene and tagalons B and C, two 16-nor-pimaranes, were isolated from the Chinese mangrove *Ceriops tagal* (Rhizophoraceae) stems and twigs [99].

**Figure 66:**
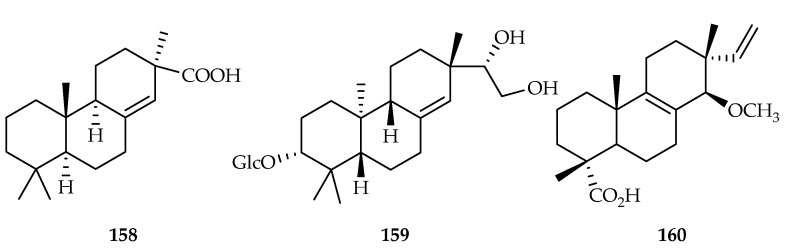
Structures of agalong C **158** [99], siegeside B **159** [103], and euonymusisopimaric acid B **160** [104].

Among them, only tagalon C **158** exhibited selective cytotoxicity against MT-1 cells, with IC_50_ value of 3.75 μM, using cisplatin as a positive control (Figure 66).

Diterpenoid glycosides, siegesides A–D were isolated and characterised from the ethanol extract of *Siegesbeckia pubescens* (Asteraceae) aerial parts, widely distributed in China [103]. All isolates were evaluated for their inhibition on the migration of the MB-MDA-231 cells induced by the chemokine epithelial growth factor, and siegeside B **159** showed superior inhibitory activities, in comparison with the positive control drug (2-morpholin-4-yl-8-phenylchromen-4-one) with an IC_50_ value of 0.27 μM (Figure 66).

Euonymusisopimaric acids A–F and euonymusone A were isolated from the stems of *Euonymus oblongifolius* (Celastraceae). All the isolated compounds were evaluated for their cytotoxic activity against five human cancer cell lines, but only euonymusisopimaric acid B **160** exhibited cytotoxicity against A549 cell line growth, with an IC_50_ value of 2.6 μM [104] (Figure 66).

**Figure 67:**
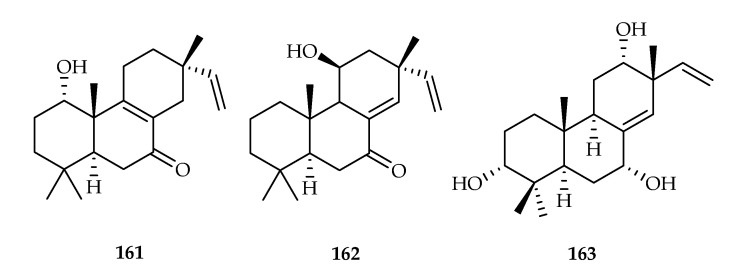
Structures of compounds **161** [105] and **162** [105] and alatavnol A **163** [106].

Two new isopimarane diterpenoids, 1α-hydroxy-7-oxoisopimara-8,15-diene **161** and 11β-hydroxy-7-oxoisopimara-8(14),15-diene **162**, were isolated by ethanolic extraction and subsequent partitioning with petroleum ether, EtOAc and *n*-BuOH, from the air-dried branches and leaves of *Salacia cochinchinensis* (Celastraceae), a perennial shrub mainly distributed in countries in south-east Asia, such as China, Vietnam, and Cambodia [105]. Their structures were assigned by comprehensive analyses of IR, MS, HRESIMS, 1D, and 2D NMR spectral data. The two compounds possessed significant cytotoxic activity against the HepG2, HL60, and HeLa cell lines, with IC_50_ values ranging from 0.23 to 4.29 μM, using cisplatin as a positive control (Figure 67).

Alatavnol A **163**, a new isopimarane diterpene (Figure 67), was isolated from the whole areal part of *Ephorbia alatavica* (Euphorbiaceae). A plausible biosynthetic pathway for the new compound **163** was also proposed. Alatavnol A **163** showed potential cytotoxic activities, with IC_50_ values of 14.327 and 12.033 μM, against the MCF-7 and A549 cell lines respectively, using doxorubicin as a positive control [106].

#### 3.2.6. Podocarpane-Type Diterpenoids

Podocarpane diterpenoids usually possess a backbone of seventeen carbons arranged in a tricyclohexane system close to abietane and pimarane.

**Figure 68:**
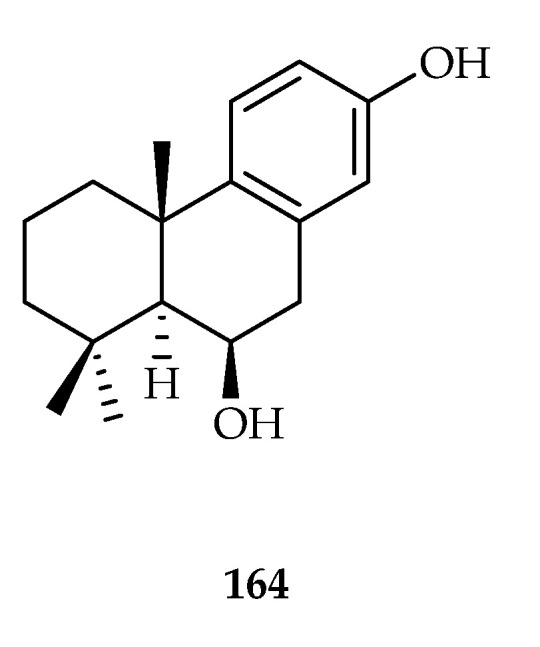
Structure of anemhupehin A **164** [107].

Anemhupehin A **164**, a new podocarpane diterpenoid, was isolated from aerial parts of *Anemone hupehensis* (Ranunculaceae). Compound **164** showed moderate activity against SW480 cell line with IC_50_ value of 13.2 μM, using cisplatin as a positive control [107] (Figure 68).

#### 3.2.7. Staminane-Type Diterpenoids

**Scheme 8:**
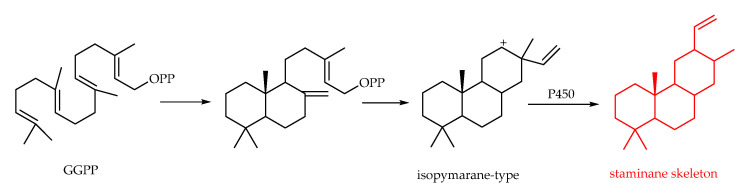
Formation of staminane skeleton.

Started from geranylgeranyl diphosphate (GGPP), isopimarane-type diterpenoids can be formed by the both protonation-initiated cyclization and ionization-dependent cylization of GGPP. With the possible catalyzation of cytochrome P-450, the isopimarane-type diterpenoids acted as a precursor, which can be transformed into staminane-type diterpenoids through the migration of the vinylic group from C-13 to C-12 (Scheme 8).

**Figure 69:**
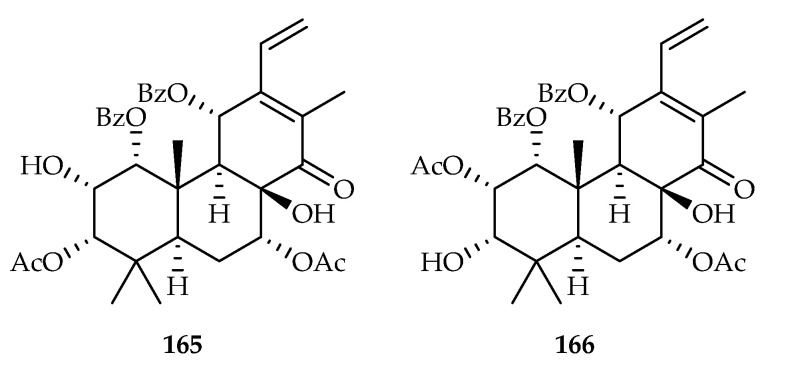
Structures of spicatusenes B **165** and C **166** [108].

This is the proposed biosynthetic mechanism for the formation of the two new diterpenoids spicatusenes, B **165** and C **166**, which were isolated from the aerial parts of *Clerodendranthus spicatus* (Lamiaceae). *C. spicatus* is one of the oldest popular herbs distributed in Southeast Asia, and it is extensively used as a traditional medicine for the treatment of many disorders, especially for kidney diseases [108] (Figure 69). Extraction was performed with 80% ethanol and subsequent partitioning with chloroform and ethyl acetate. Their structures were identified by spectroscopic methods. Their cytotoxic activities were tested against the HL-60, SMMC-7721, A549, MCF-7, and SW-480 cell lines, and they showed significant inhibitory activities toward all of the five human cancer cell lines, with IC_50_ values in the range of 5.62–36.60 μM, using cisplatin and taxol as positive controls.

### 3.3. Tetracyclic Diterpenes

#### 3.3.1. Atisane-Type Diterpenoids

**Figure 70:**
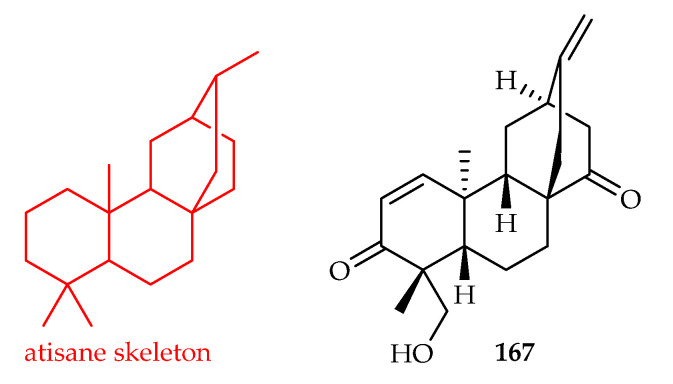
Atisane skeleton and structure of phorneroid H **167** [86].

A new *ent*-atisane-type diterpenoid, named phorneroid H **167**, with cytotoxic activity, was isolated from the *Euphorbia neriifolia* (Euphorbiaceae) aerial parts, together with *ent*-abietane-type diterpenoids (see Section 3.2.1). It showed an IC_50_ value of 4.1 and 4.0 μM against A549 and HL-60 cancer cells, respectively, using adriamycin as a positive control [86] (Figure 70).

#### 3.3.2. Beyerane-Type Diterpenoids

**Figure 71:**
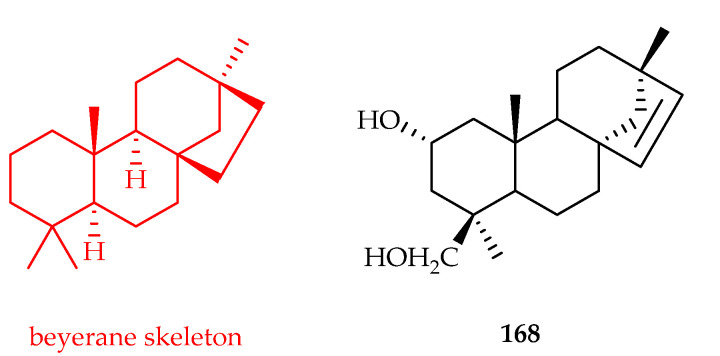
Structures of beyerane skeleton and aleuritopsis A **168** [109].

One new beyerene-type diterpenoid, named aleuritopsis A **168**, together with a new *ent*-kaurane-type diterpenoid, named aleuritopsis B (see Section 3.3.4), was extracted with ethanol and separated from the whole plant of *Aleuritopteris argentea* (Gmél.) Fée (Sinoptiridaceae), which is distributed in most parts of China [109]. Their structures and absolute configurations were characterised by UV, FT-IR, NMR, MS, and single-crystal X-ray diffraction. The cytotoxic activity of aleuritopsis A **168** was tested on the MCF-7 and SKOV3 cell lines using the MTT method. The IC_50_ values of the inhibition ratio were calculated by the Reed and Muench’s method. Aleuritopsis A **168** showed inhibitory activities against SKOV3 cells, with an IC_50_ value of 11.1 μM, using cisplatin as a positive control (Figure 71).

#### 3.3.3. Cephalotane-Type Diterpenoids

Cephalotane diterpenoids are a class of compounds within the Cephalotaxus genus that have been gaining increasing attention, especially in recent years, due to their unique and complicated carbon skeletons and remarkable antitumor activities. Their structure and biological activity were recently summarised in a review, and their biosynthetic pathway were described [110].

**Figure 72:**
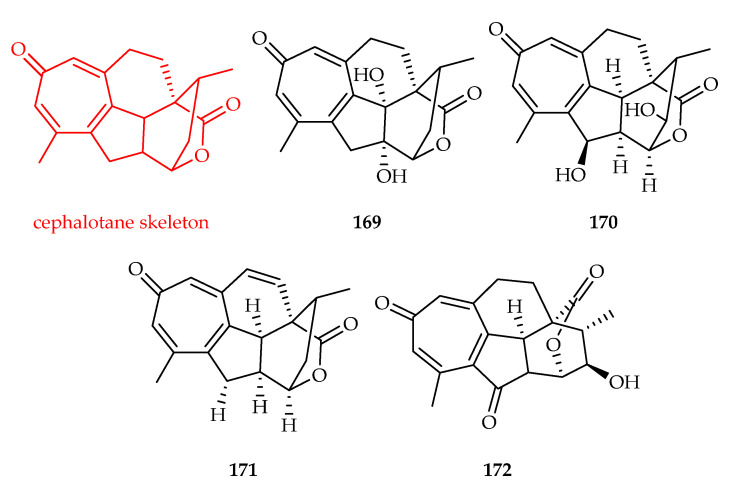
Structures of cephalotane skeleton, fortalpinoids E, F, and H **169**–**171** [111], and 20-Oxohainanolidol **172** [112].

Seventeen new 17-nor-cephalotane-type diterpenoids, fortalpinoids A-Q, were isolated from the seeds of *Cephalotaxus fortunei* var. alpine (Taxaceae) by extraction with EtOH/H_2_O (95:5) and subsequent partitioning in EtOAc and petroleum ether [111]. The new compounds were tested against the cancer cell lines HL60 and A549 using the CCK-8 assay. Fortalpinoids E, F, and H **(169–171)** showed significant inhibitory activities against both cell lines, with IC_50_ values ranging from 1.0 to 9.8 μM, using adriamycin as the positive control. The tropone ring seems to be essential for the anticancer activity, since compounds lacking the tropone ring were inactive (Figure 72).

A new cephalotaxus troponoid (Figure 72), 20-oxohainanolidol **172**, was identified from the twigs and leaves of *Cephalotaxus fortunei* (Taxaceae). Compound **172** exhibited cytotoxic activities against HL60 and A549 cells, with IC_50_ values of 0.77 and 1.129 μM, respectively, using doxorubicin as a positive control [112].

**Figure 73:**
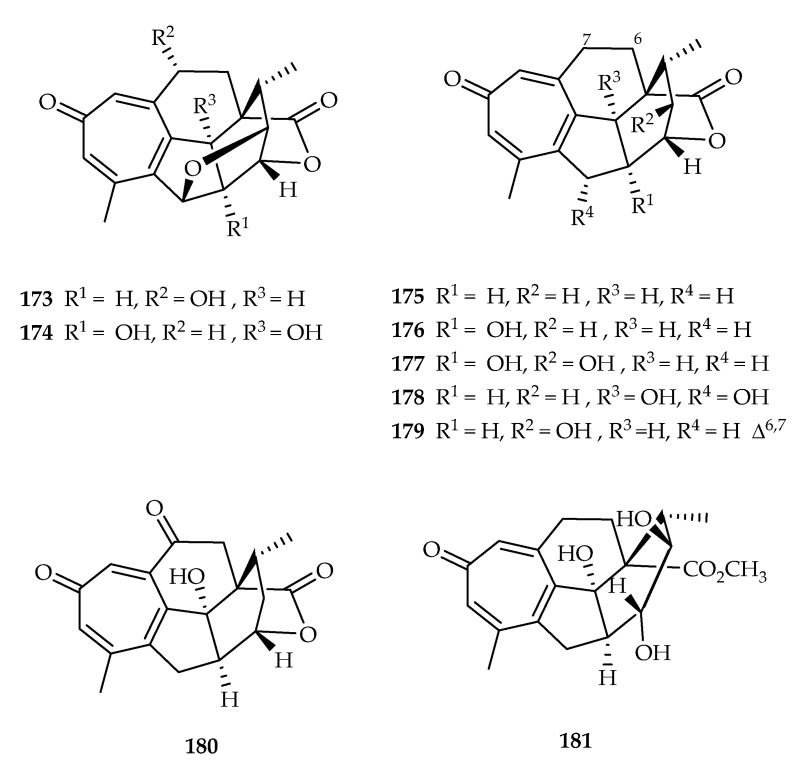
Structures of cephinoids F–L **173**–**179** [113] and N-O **180–181** [113].

The Cephalotaxus tropone analogues, cephinoids A-S (Figure 73), were identified from *Cephalotaxus fortunei* var. alpina and *C. lanceolata* (Taxaceae) leaves and twigs. The cytotoxic activities of the tested compounds **173–181** were evaluated against the HeLa, SGC7901, and A549 cell lines. In particular, cephinoids H **175** and I **176** exhibited excellent inhibitory effects, with IC_50_ values ranging from 0.1 to 0.71 μM, using cisplatin as a positive control. The IC_50_ values were calculated by the Reed and Muench method [113].

#### 3.3.4. Kaurane-Type Diterpenoids

**Figure 74:**
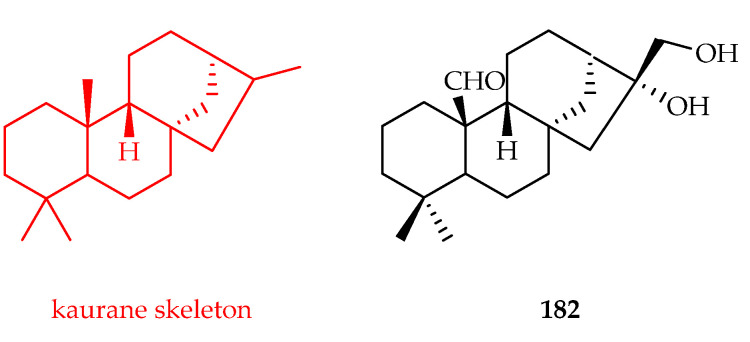
Structures of kaurane skeleton and fischericin D **182** [119].

Diterpenes of the kaurane family (Figure 74) are present in one of the most popular beverages, which is coffee; the two commercially exploited species of coffee, *Coffea arabica* (arabica) and *Coffea canephora* (var. Robusta), have been extensively studied. Among the many compounds identified, there are three important diterpenes, such as cafestol, 16-*O*-methylcafestol and kahweol, which belong to the kaurene family [114], that have several biological activities [115,116]. In particular, 16-*O*-methylcafestol is considered a marker of the species *Coffea canephora* [117]. Since the first discovery in 1961, more than 1300 ent-kaurane diterpenoids have been isolated and identified from different plant sources, mainly the genus *Isodon*. A large number of reports describe the anticancer potential and mechanism of action of *ent*-kaurane compounds in a series of cancer cell lines [118].

Six new diterpenoids were isolated by methanolic extraction and susequent partitioning in EtOAc from the whole plant of *Ligularia fischeri* (Ledeb.) Turcz (Compositae), a perennial leafy edible plant distributed in wet shady regions of Europe and Asia that has been used to treat jaundice, hepatic problems, scarlet fever, coughs, and hepatic diseases [119]. Among the new isolated compounds, fischericin D **182** showed moderate inhibitory activity against human B lymphoblast HMy2.CIR cells in a dose-dependent manner at concentrations of 2.5–40 μM. It showed an IC_50_ value of 13.3 μM using the sulforhodamine B (SRB) method (Figure 74). Its structure was elucidated by spectroscopic analysis, and the absolute configuration was determined by single-crystal X-ray diffraction.

**Figure 75:**
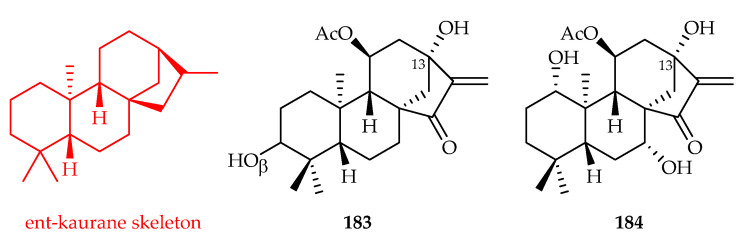
Structures of *ent*-kaurane skeleton, 3-e*pi*-isodopharicin A **183** [120], and scopariusol C **184** [120].

Two C-13-oxygenated *ent*-kauranes, 3-e*pi*-isodopharicin A **183** and scopariusol C **184**, with cytotoxic activity were isolated by extraction with 70% aqueous acetone and subsequently partitioned in water-EtOAc from the aerial parts of *Isodon scoparius* (Lamiaceae) present in China [120] (Figure 75). The two compounds were active against the HL-60, SMMC-7721, A549, MCF-7, and SW-480 cell lines, using the 3-(4,5-dimethylthiazol-2-yl)-5-(3-carboxymethoxyphenyl)-2-(4-sulfophenyl)-2H-tetrazolium (MTS) method and cisplatin and paclitaxel as positive controls. Compound **183** showed significant cytotoxic activity against all the cancer cell lines, with IC_50_ values of 1.0, 1.5, 4.4, 2.9, and 0.9 μM, respectively, better than cisplatin, while compound **184** showed IC_50_ values of 7.0, 4.7, 19.6, 11.0, and 2.5 μM, respectively, with a stronger effect than cisplatin for the cell lines SMMC-7721, MCF-7, and SW-480.

**Figure 76:**
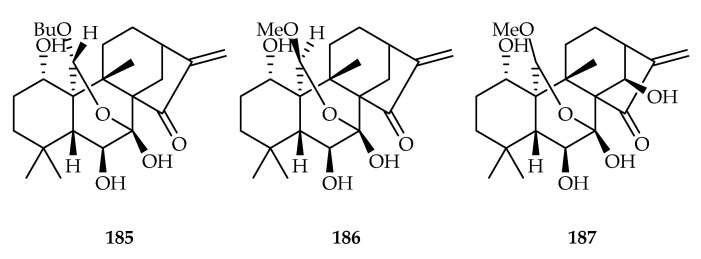
Structures of isojiangrubesins B **185** [121], isojiangrubesins C **186** [121], and isojiangrubesins E **187** [121].

Seven previously undescribed 7,20-epoxy-*ent*-kaurane diterpenoids, named isojiangrubesins A-G, were isolated from the aerial parts of *Isodon rubescens* (Lamiaceae), a perennial herb widely distributed in the Yellow River Basin and southern area of China, by extraction with 70% aqueous acetone [121]. Their structures were characterised on the basis of spectroscopic methods and signal-crystal X-ray diffraction. All of these compounds were evaluated for their in vitro cytotoxicity against the HL-60, SMMC-7721, A549, MCF-7, and SW480 cell lines using the MTS assay, but only isojiangrubesins B **185**, C **186**, and E **187** exhibited potent active effects against all cell lines, with IC_50_ values in the range 0.8–8.6 μM, using cisplatin and paclitaxel as reference compounds (Figure 76).

**Figure 77:**
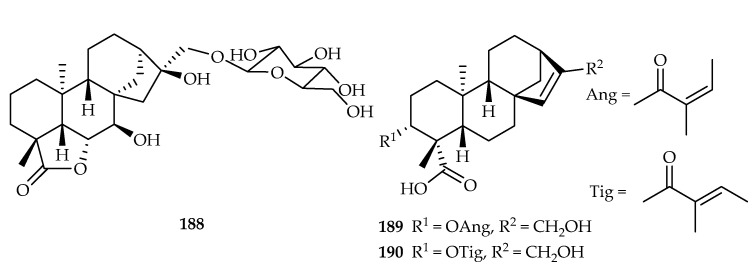
Structures of pharboside H **188** [122] and compounds **189–190** [123].

Pharboside H **188**, an *ent*-kaurane-type diterpene glycoside with a γ-lactone-ring in the structure, was isolated from the seeds of *Pharbitis nil* (Convolvulaceae) present in Korea (Figure 77). Pharboside H **188** showed cytotoxicity against the SKOV3, SK-MEL-2 (human melanoma), and HCT15 cell lines, with IC_50_ values of 5.92, 15.31, and 17.27 μM, respectively, using etoposide as a positive control [122].

Compounds **189–190** (Figure 77) were isolated from the *Wedelia prostrata* (Asteraceae) herbs present in China and exhibited cytotoxic activity against HepG2 cells, with IC_50_ values of 16.94 and 12.38 μM, respectively, using the MTT assay and cisplatin as a positive control [123].

**Figure 78:**
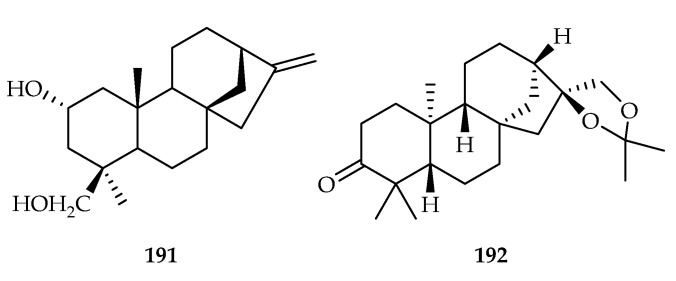
Structures of aleuritopsis B **191** [109] and compound **192** [124].

One new *ent*-kaurane-type diterpenoid, named aleuritopsis B **191**, together with the new beyerene-type diterpenoid aleuritopsis A **168** (see Section 3.3.2), were extracted with ethanol and separated from the whole plant of *Aleuritopteris argentea* (Gmél.) Fée (Sinoptiridaceae), which is distributed in most parts of China [109] (Figure 78). Their structures and absolute configurations were characterised by UV, FT-IR, NMR, MS, and single-crystal X-ray diffraction. Cytotoxic activity of aleuritopsis B **191** was tested on MCF-7 and SKOV3 cell lines using the MTT method, and the IC_50_ values of the inhibition ratios were calculated by Reed and Muench’s method. Aleuritopsis B **191** showed inhibitory activities against the SKOV3 and MCF-7 cell lines, with IC_50_ values of 8.7 and 11.3 μM, respectively, using cisplatin as a positive control.

A similar compound, *ent*-kaurane-3-oxo-16b, 17-acetonide **192**, was isolated by ethanolic extraction of the dried roots of *Euphorbia fischeriana* Steud (Euphorbiaceae) [124], a plant mainly growing in the northeast of China and used in traditional Chinese medicine to treat many diseases, such as chronic tracheitis, tuberculosis, and tumors. Its structure was determined by 1D NMR, 2D NMR, and HRESIMS analyses (Figure 78). The cytotoxic activity of **192** was tested against the growth of the Hep3B and A549 cell lines by the MTT assay. In the cytotoxic assays on the Hep3B cell line, **192** showed an IC_50_ value of 8.1 μM, which was stronger than the positive control, 5-FU.

**Figure 79:**
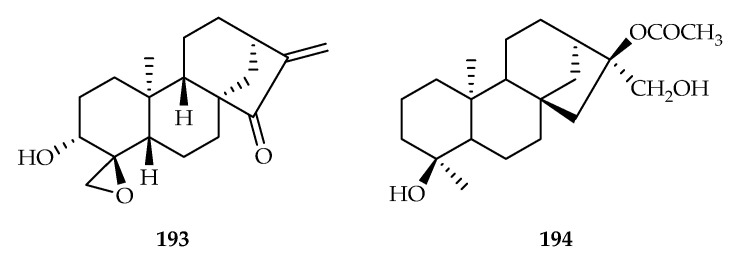
Structures of amentotaxin C **193** [55] and compound **194** [125].

Eleven *ent*-kaurane-type diterpenoids (amentotaxins C-M) were isolated, by methanolic extraction and subsequent partitioning in different solvents, from the leaves and twigs of *Amentotaxus argotaenia* (Taxaceae), a coniferous species endemic to China [55]. Their structures were elucidated on the basis of spectroscopic data, single-crystal X-ray diffraction, the modified Mosher’s method, and ECD data analyses. They were evaluated for their cytotoxic effects against the HeLa, A549, MDA-MB-231, SKOV3, Huh-7 (human hepatoma), and HCT-116 cell lines; additionally, amentotaxin C **193** showed cytotoxic activity against all six cell lines, with IC50 values ranging from 1.9 to 9.8 μM, using paclitaxel as the positive control. Compound **193** is a rare 18-nor-*ent*-kaurane-type diterpenoid featuring a 4β,19-epoxy ring (Figure 79).

Five new *ent*-kaurane diterpenes were isolated from *Annona squamosa* L. (Annonaceae) pericarp, and they were evaluated for their cytotoxic activities against the SMMC-7721 and HepG2 cell lines, among which, 4β,17-dihydroxy-16α-acetoxy-18-nor-*ent-*kaurane **194** displayed powerful anti-tumor capacity against the SMMC-7721 and HepG2 cell lines, with IC_50_ values of 17.28 and 11.56 μM, respectively [125] (Figure 79).

**Figure 80:**
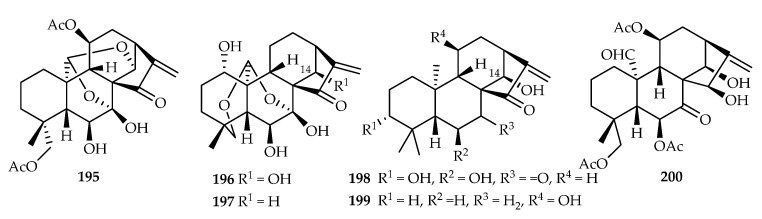
Structures of 19-acetyl xerophilusin A **195**, 1α-hydroxy xerophilusin D **196**, 1α-hydroxy-14-dehydroxy xerophilusin D **197**, pharicusin D **198**, pharicusin E **199**, and pharicusin F **200** [126].

*Ent*-kauranoid diterpenes 19-acetyl xerophilusin A **195**, 1α-hydroxy xerophilusin D **196**, 1α-hydroxy-14-dehydroxy xerophilusin D **197**, pharicusin D **198**, pharicusin E **199**, and pharicusin F **200** (Figure 80) were isolated from the aerial parts of *Isodon pharicus* (Lamiaceae), which is primarily distributed in the northwest district of Sichuan Province and the southern district of Tibetan Region [126]. Compounds **195–200** were evaluated for the in vitro growth inhibitory effects against HL-60, SMMC-7721, A549, MCF-7, and SW-480 cell lines, with cisplatin and paclitaxel as the positive controls. Compound **199** exhibited significant inhibitory effects against all five human tumor cell lines, with IC_50_ values ranging from 2.20 to 5.61 μM, whereas compounds **195**, **197**, and **198** showed some cytotoxic potency, indicating that the α,β-unsaturated ketone group is structurally required for cytotoxicity. Compound **196** also contained this motif, but almost showed no cytotoxicity, possibly due to the hydroxy group substitution at C-14. Despite having no α,β-unsaturated ketone group, compound **200** exhibited significant inhibitory activities against all five human tumor cell lines, with IC_50_ values ranging from 2.58 to 6.03 μM, suggesting that an improvement of the lipid solubility might aid in promoting the cytotoxic potency.

**Figure 81:**
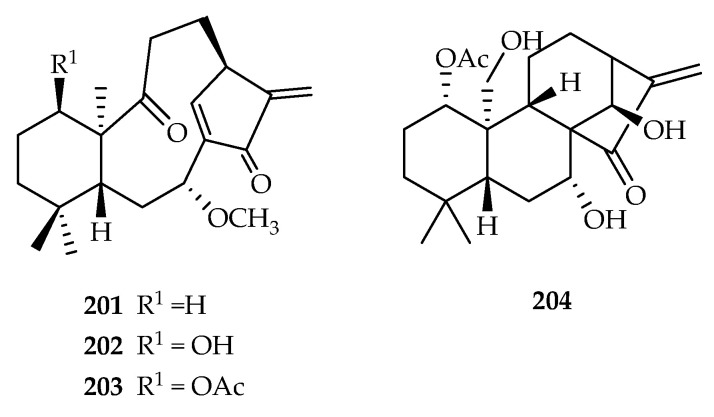
Structures of kongeniods A–C **201**–**203** [127] and compound **204** [128].

Three new 8,9-*seco*-*ent*-kaurane diterpenoids, kongeniods A–C **201**–**203** (Figure 81), were isolated from the aerial parts of *Croton kongensis* (Euphorbiaceae). Compounds **201**–**203** were evaluated in vitro against the HL-60 and A549 cell lines, with adriamycin as the positive control. Kongeniods A–C **201**–**203** exhibited strong cytotoxicity against the HL-60 cell line, with IC_50_ values of 1.27, 0.47, and 0.58 μM, respectively; compounds **201** and **202** showed moderate inhibition against the A549 cell line, with IC_50_ values of 5.74 and 3.24 μM, respectively [127].

The novel compound 1α-acetoxy-7α, 14β, 20α-trihydroxy-*ent*-kaur-16- en-15-one **204** was isolated from the aerial parts of *Isodon excisoides* (Lamiaceae) using an orientational preparation method based on a UPLC-LTQ-Orbitrap-MS [128] (Figure 81). Its cytotoxic effect was tested against the HCT-116, HepG2, BGC-823 (**human gastric cancer**), NCI-H1650 (human lung adenocarcinoma bronchioalveolar carcinoma), and A2780 cell lines, and it showed cytotoxic activity, with IC_50_ values of 1.34, 1.07, 3.60, 2.35, and 2.53 µM, respectively, using cisplatin as the positive control.

**Figure 82:**
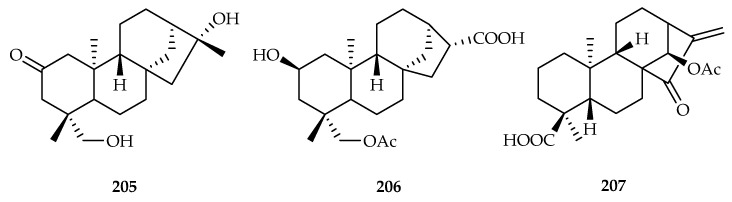
Structures of maizediterpene A **205** [129], maizediterpene B **206** [129], and rabdokunmin F **207** [83].

Four new *ent*-kaurane diterpenoids, named maizediterpenes A-D, were isolated from the roots of *Zea mays* (Poaceae) by 70% EtOH extraction and subsequent partitioning with petroleum ether, EtOH, and *n*-BuOH [129]. Cytotoxicity was tested against the A549, MDA-MB-231, SK-Hep-1 (liver cancer), SNU638 (stomach cancer), and HCT116 cell lines, using the SRB assay and etoposide as a positive control. Maizediterpene A **205** was active against the SNU638 and HCT116 cell lines, with IC_50_ values of 19.5 and 19.6 μM, respectively, while maizediterpene B **206** was more active then etoposide, with IC_50_ values of 15.18 and 1.99 μM against MDA-MB-231 and HCT116, respectively, but was also active on SK-Hep-1 and SNU638, although to a minor extent (Figure 82).

Rabdokunmin F **207** is a novel *ent*-kaurene diterpene with C-18 oxidised to a carboxylic acid group, isolated from *Isodon interruptus* (Lamiaceae) leaves, together with kunminolide A **132** (see Section 3.2.1) [83]. It exhibited strong inhibitory effects against A549, KB, MCF7, and HCT116 cell lines, with IC_50_ values in the range 1.1–3.0 μM, using paclitaxel as positive control (Figure 82).

**Figure 83:**
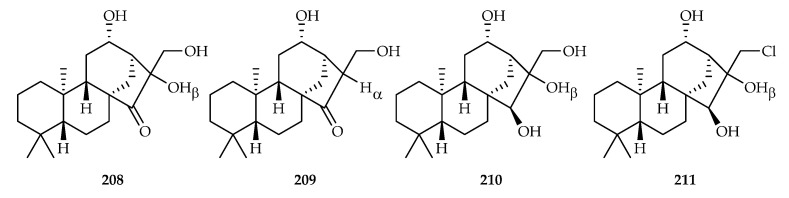
Structures of mesonols A–C **208**–**211** [130].

From *Mesona procumbens* Hemsl. (Lamiaceae) whole plant, a traditional Chinese herb, it was possible to isolate seven new *ent*-kauranes, named mesonols A–G, by methanolic extraction and subsequent partitioning with *n*-hexane and CH_2_Cl_2_ [130]. Their structures were determined by spectroscopic methods, especially 2D NMR, HRESIMS, and X-ray crystallographic analysis. Mesonols A–C **208**–**211** showed potential antiproliferative activities against A549, Hep3B, PC3, HT29, and U937 cancer cells, with IC_50_ values ranging from 1.97 to 19.86 μM, using the cell counting Kit-8 and CPT-11 as positive controls. Further investigations on cell cycle progression, apoptosis, and ROS generation in U937 human leukemia cancer cells showed that compounds **208** and **209** are potent and promising anticancer agents (Figure 83).

**Figure 84:**
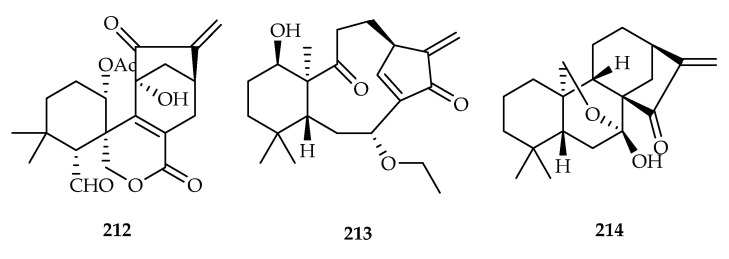
Structures of isoresbin A **212** [131] and croyanhuins A **213** and C **214** [132].

Isoresbin A **212**, with a rearranged 15 (8→11)-*abeo*-6,7-*seco*-*ent*-kaurane skeleton, was isolated from *Isodon oresbius* (W. W. Smith) Kudo (Labiatae), a small shrub distributed on dry hillside rocks or shrubs in China [131] (Figure 84). Extraction was performed with 70% aqueous acetone on the air-dried and powdered aerial parts of *I. oresbius* and subsequent partitioning with EtOAc and H_2_O. Compound **212** showed cytotoxicity against the HeLa, SK-OV-3, SK-HEP-1, Caco2, MDA-MB-231, PC-3, SW480, and A549/Taxol (taxol resistant lung cancer) cell lines, with IC_50_ values in the 2.07–14.60 μM range, using the MTS assay, cisplatin, and taxol as positive controls.

Croyanhuins A **213** and C **214** were isolated from the leaves and twigs of *Croton yanhuii* (Euphorbiaceae) by ethanolic extraction and subsequent partitioning with EtOAc and *n*-BuOH [132] (Figure 84). Their structures were determined by extensive spectroscopic methods (1D and 2D NMR, IR, and HRESIMS); compound **213** is an 8,9-*seco*-*ent*-kaurane diterpenoid, while compound **214** is a 7,20-epoxy-*ent*-kaurane diterpenoid. Compounds **213** and **214** inhibited cell proliferation and viability in a dose- and time-dependent manner, whereas both induced the cleavage of either caspase-3 or PARP-1 in the SW480 cell line. Both compounds had IC_50_ values <20 μM against the HeLa and SW480 cell lines, while compound **213** was also cytotoxic against the A549 and ACHN cell lines, using cisdiaminedichloroplatinum (CDDP) as a positive control.

**Figure 85:**
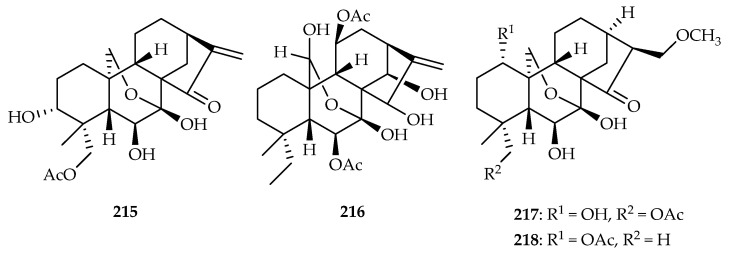
Structures of pharicin D, M, Q, and R **215**–**218** [133].

Pharicin D **215**, pharicin M **216**, pharicin Q **217**, and pharicin R **218** (Figure 85) were isolated from ethyl acetate extract of the aerial parts of *Isodon pharicus* (Labiatae). Structurally, the isolates were characterised as 7*α*,20-epoxy-*ent*-kaurane diterpenoids. Preliminary structure—activity relationship studies were evaluated, in terms of their in vitro cytotoxic activities against the HL-60, SMMC-7721, A-549, MCF-7, and SW-480 cell lines. Pharicins D, M, Q and R **215**–**218** displayed inhibitory activities, with IC_50_ values in the range of 1.23–18.04 μM, using cisplatin and paclitaxel as positive controls [133].

**Figure 86:**
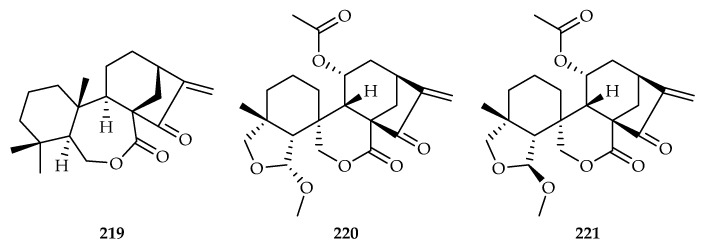
Structures of scapanacin A **219** [134], 6-*epi*-11-*O*-acetylangustifolin **220** [135], and 11-*O*-acetylangustifolin **221** [135].

Scapanacin A **219** (Figure 86) was isolated from the Chinese liverwort *Scapania carinthiaca* J.B. Jack ex Lindb. (Scapaniaceae) whole plant. Scapanacin A **219** showed significant inhibitory effects against the MCF-7, A549, and PC-3 cell lines, with IC_50_ values of 6.6, 2.3, and 11.5 μM, respectively, using adriamycin as a positive control [134].

6-*Epi*-11-*O*-acetylangustifolin **220** and 11-*O*-acetylangustifolin **221**, two 6,7-*seco*-spiro-lacton-*ent*-kauranoids, were isolated from the branches and leaves of *Isodon rubescens* (Lamiaceae) (Figure 86). The 11-*O*-acetylangustifolin **221** was previously reported as the acetylation product of angustifolin. Compounds **220** and **221** were tested for their cytotoxicity against the A549 and K562 cell lines. Compared to positive control adriamycin, both diasteroisomers **220** and **221** showed moderate cytotoxicity against A549 cells, with IC_50_ values of 15.81 and 9.89 μM, respectively, while compounds **220** and **221** displayed significant cytotoxicity against K562 cells, with IC_50_ values of 1.93 and 0.59 μM, respectively [135].

**Figure 87:**
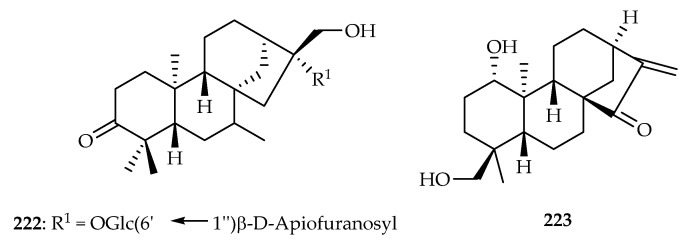
Structures of diosmarioside D **222** [136] and scopariusol L **223** [137].

Diosmarioside D **222**, a new glycoside based on *ent*-kaurane-type diterpenoid, was isolated from the leaves of *Diospyros maritima* (Ebenaceae) (Figure 87). Diosmarioside D **222** showed strong activity, with an IC_50_ value of 5.11 μM against the A549 cell line, using etoposide as a positive control [136].

Scopariusol L **223** (Figure 87) was isolated from the aerial parts of *Isodon scoparius* (Lamiaceae), and its absolute configuration was established by a single crystal diffraction analysis using Cu Kα radiation. The new *ent*-kaurane diterpenoid **223** showed stronger cytotoxicity than cisplatin, with respective IC_50_ values of 2.8, 3.4, 3.6, 4.0, and 2.6 μM against the HL-60, SMMC-7721, A549, MCF-7, and SW-480 cell lines [137].

**Figure 88:**
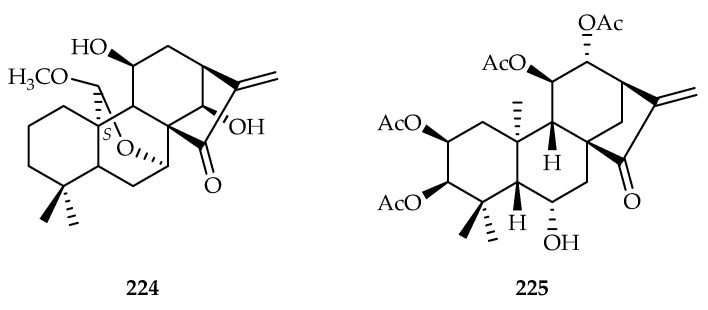
Structures of JDA-202 **224** [138] and IsoA **225** [139].

JDA-202 **224**, an *ent*-kaurene diterpenoid isolated from *Isodon rubescens* (Lamiaceae) leaves, proved to possess strong anti-proliferative activities on the esophageal cancer (EC) cell line (Figure 88). JDA-202 **224** not only induced EC cell apoptosis but also inhibited tumor growth by targeting the over-activated antioxidant protein Prx I (peroxiredoxin I). The cytotoxicity of **224** was determined on the human esophageal cancer cell lines EC109, EC9706, KYSE-450, and human immortalised normal esophageal epithelial cell HET-1A and it showed IC_50_ values of 8.6, 9.4, 26.2, and 36.1 μM, respectively, using the natural product adenanthin as positive control. Furthermore, JDA-202 showed inhibitory activity against SHG-44 (human glioblastoma) and MCF-7 cell lines with IC_50_ values of 2.4 and 4.2 μM, respectively [138].

Isoforretin A **225** (IsoA) (Figure 88), a novel *ent*-kaurane isolated from the leaves of *Isodon forrestii* (Lamiaceae), significantly inhibited Thioredoxin 1 (Trx1) activity and mediated anticancer effects in multiple preclinical settings. IsoA **225** displayed antiproliferative activity with IC_50_ values < 20μM against HepG2, Caco2, U2OS (human osteosarcoma), and MDA-MB-231 cell lines, while it was not cytotoxic to non-malignant human hepatic cells (IC_50_ 113μM). Of all the tested cancer cell lines, HepG2 cells were the most sensitive to IsoA **225**, with an IC_50_ value of 15.83 μM. Thus, this cancer cell line was chosen as a model to further investigate the anticancer activity and underlying mechanisms of IsoA **225** [139].

**Figure 89:**
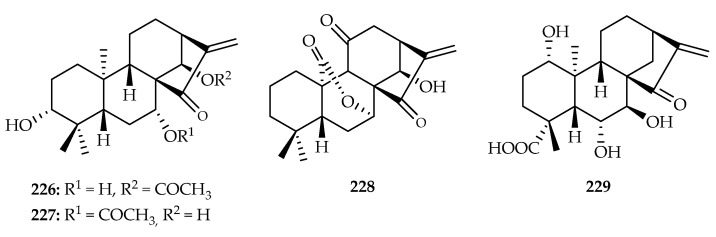
Structure of compound **226**, **227** [140], Jaridon 6 **228** [141], and pteisolic acid G **229** [142].

Two new *ent*-kauranoid-type diterpenoids **226** and **227** (Figure 89) were obtained from the aerial parts of *Rabdosia japonica* (Lamiaceae). Their chemical structures were established by 1D and 2D NMR techniques and mass spectrometry. They were tested for their cytotoxic effects against the A549, HCT116, CCRF-CEM, and HL-60 cell lines. Compounds **226** and **227** showed significant inhibitory activities toward the four human cancer cell lines, with IC_50_ values in the range 3.01–15.45 μM, using doxorubicin as a positive control [140].

A novel *ent*-kaurene diterpenoid, Jaridon 6 **228** (Figure 89), was isolated from *Rabdosia rubescens* (Lamiaceae). The results of the MTT assay showed that Jaridon 6 **228** treatment had an obvious cytotoxic effect in EC109 cells, with an IC_50_ of 15.59 μM, whereas the cytotoxic effect on normal HET-1A cells was minimal. Cell viability and apoptosis results, obtained by flow cytometry, confirmed the tumor inhibitory effect of Jaridon 6 **228** in esophageal cancer cells [141].

Pteisolic acid G **229** (Figure 89) was isolated from *Pteris semipinnata* (Pteridaceae) aerial parts showed significant cytotoxicity against the HCT116 cell line in a dose- and time-dependent manner, with IC_50_ values of 4.07 μM after 72 h, using a CCK-8 assay and TBE test. The effects of pteisolic acid G **229** on the cell cycle and apoptosis in HCT116 cells were investigated [142].

## 4. Prenylsesquiterpenes

### Prenyleudesmane-Type Diterpenoids

**Figure 90:**
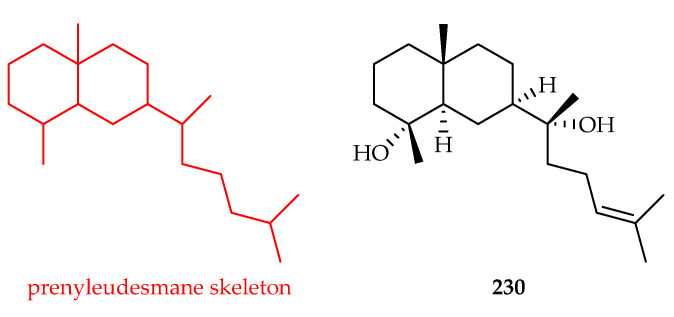
Structures of prenyleudesmane skeleton and lonimacranthoidin C **230** [143].

Prenyleudesmanes are a rare class of diterpenes that were originally isolated from marine algae and mollusks, fungi, and plants of the genus *Dysoxylum* [143]. Two prenyleudesmanes, lonimacranthoidin C **230** and lonimacranthoidin D, were isolated from the roots of *Lonicera macranthoides* (Caprifoliaceae), which is mainly distributed in the southwest of China (Figure 90). Their structures were established based on 1D and 2D NMR spectra and high-resolution electrospray ionization mass spectral (HR-ESI-MS) data. The absolute configuration of **230** was determined by X-ray diffraction. They were tested for their antiproliferative effect against HepG2, HeLa, and the human aortic smooth muscle cell line (HASMC). Only lonimacranthoidin C **230** showed moderate antiproliferative activity against the HeLa cell lines, with an IC_50_ value of 13.8 μM, using etoposide as positive control, while no cytotoxicity was observed for the normal cells HASMC.

## 5. Other Diterpenoid Skeletons

### 5.1. Norditerpenoids and Dinorditerpenoids

**Figure 91:**
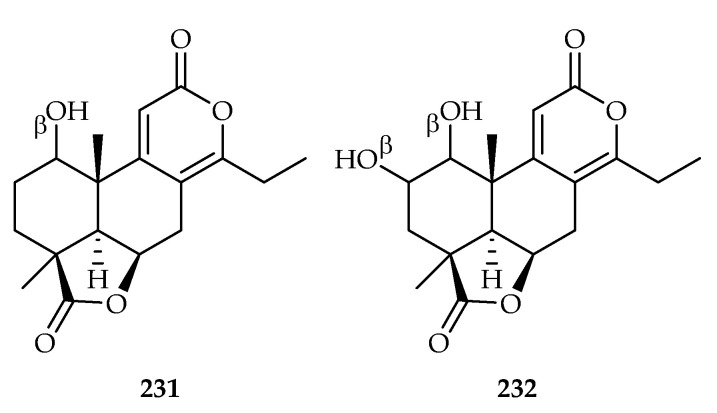
Structures of nagilactones L **231 [144]** and 2β-hydroxynagilactone L **232 [144]**.

Nine new norditerpenoids were isolated from a 95% ethanolic extract of the seeds of *Podocarpus nagi* (Thunb.) Pilg. Herein (Podocarpaceae) [144]. The structures of the new compounds were established based on detailed NMR and HRESIMS analysis, as well as from their ECD spectra. All of the isolates were tested for their cytotoxic activities against cancer cells. The results indicated that nagilactone L **231** and 2β-hydroxynagilactone L **232** displayed cytotoxicity against A2780 and HEY cancer cells. Compound **232** showed the highest potential, with IC_50_ values of less than 2.5 μM on the two cell lines. Compound **231** showed IC_50_ values of 3.8 μM against A2780 cells and 6.4 μM against HEY cells, respectively (Figure 91).

**Figure 92:**
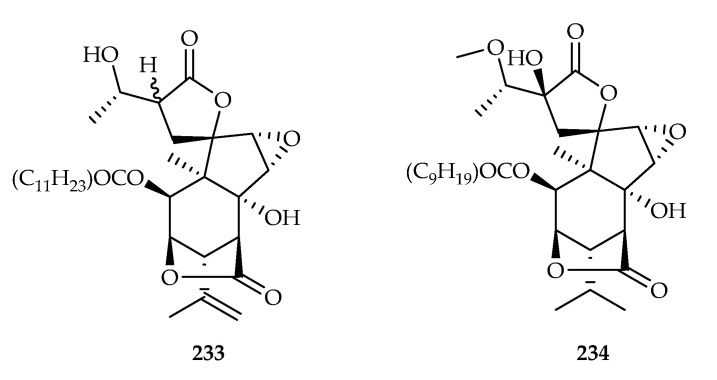
Structures of austrobuxusin F **233** [145] and austrobuxusin L **234** [145].

Twelve structurally related “norditerpenes”, named austrobuxusins E–M and 16-*epi*-austrobuxusins B, G, and H, were isolated from the EtOAc fruit extract of the species *Austrobuxus carunculatus* (Baill.) Airy Shaw (Picrodendraceae), a plant endemic to New Caledonia [145]. All compounds were tested against the HCT116, U87-MG (glioblastoma), and A549 cell lines. In particular, austrobuxusin F **233** and austrobuxusin L **234** showed IC_50_ values of 5.8 μM for the A549 cell line and 0.7 μM for the U87-MG cell line, respectively. Doxorubicin was used as a positive control (Figure 92).

**Figure 93:**
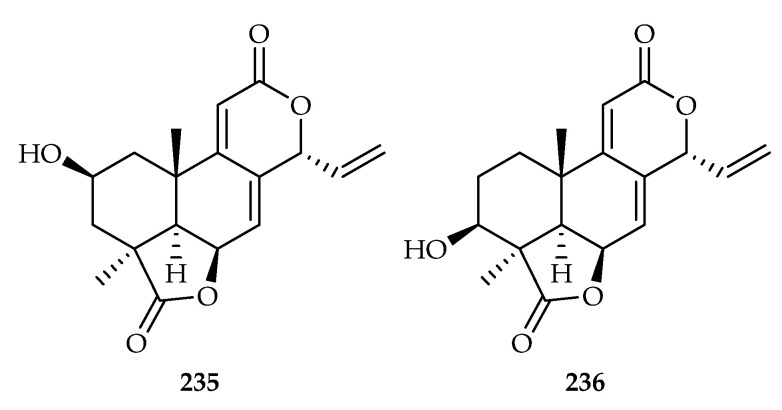
Structures of 2β-hydroxymakilactone A **235** [146] and 3β-hydroxymakilactone A **236** [146].

Two new dinorditerpene dilactones, 2β-hydroxymakilactone A **235** and 3β-hydroxymakilactone A **236**, were isolated from the roots of *Podocarpus macrophyllus* (Podocarpaceae) (Figure 93). The structures of **235** and **236** were elucidated by spectroscopic analysis, including 1D NMR, 2D NMR, and HR-ESI-MS data. Compounds **235** and **236** were evaluated for their cytotoxicities against the AGS, HeLa, MDA-MB-231, HepG-2, and PANC-1 cell lines, using cisplatin as a positive control. The 2β-Hydroxymakilactone A **235** exhibited significant activities, with IC_50_ values of 0.38, 0.87, and 4.23 μM against AGS, HeLa, and MDA-MB-231 cells, respectively, while 3β-hydroxymakilactone A **236** exhibited obvious activities, with IC_50_ values ranging from 11.61 to 0.88 μM against all the five cell lines [146].

### 5.2. Vibsane-Type Diterpenoid

Vibsane-type diterpenoids are rare 6–11 membered ring polysubstituted macrocyclic diterpenoids, and their natural occurrence is confined to the genus *Viburnum* and liverwort *Odontoschisma denudatum.* Their plausible biogenetic pathway starts from GGPP, which cyclise to form a 11-membered ring, as described in a recent review [147].

**Figure 94:**
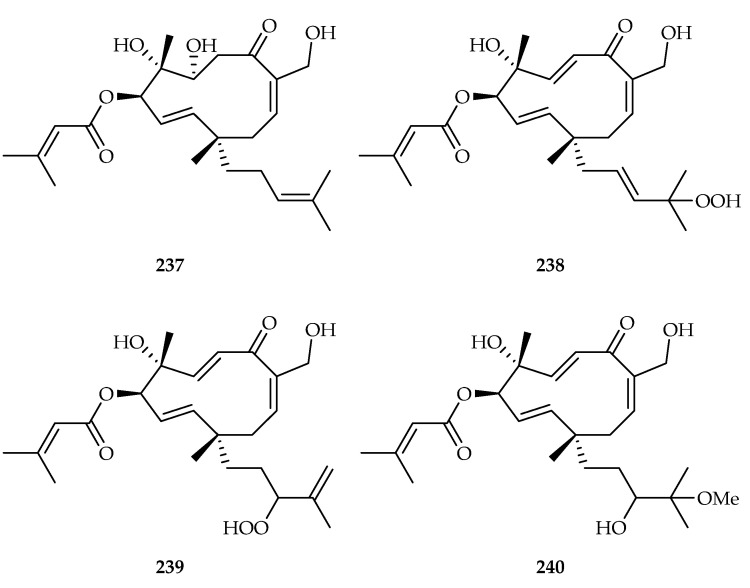
Structures of vibsanol I **237** [148], 15-hydroperoxyvibsanol A **238** [148], 14-hydroperoxyvibsanol B **239** [148], and 15-*O*-methylvibsanin U **240** [148].

Four new eleven-membered vibsane-type diterpenoids, vibsanol I **237**, 15-hydroperoxyvibsanol A **238**, 14-hydroperoxyvibsanol B **239**, and 15-*O*-methylvibsanin U **240** (Figure 94) were isolated from the twigs and leaves of *Viburnum odoratissimum* (Viburnaceae). Their structures have been elucidated by spectroscopic analyses and the chemical derivatization method. All the compounds showed different levels of cytotoxicity against the cell lines HL-60, A-549, SMMC-7721, MCF-7, and SW480, with IC_50_ values in the range of 2.56–27.06 μM, with cisplatin and taxol as positive controls [148].

**Figure 95:**
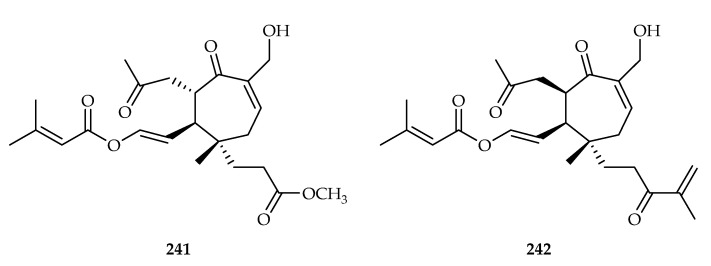
Structures of vibsanolid B **241** [149] and C **242** [149].

Further, seven new diterpenoids, named vibsanolid A–G, were isolated from the crude extracts of the leaves of *Viburnum odoratissimum* in 2021, using small molecule accurate recognition technology (SMART). Their structures were elucidated by means of comprehensive analyses of spectroscopic data, as well as comparison of the experimental and calculated ECD spectra. The cytotoxic activity was evaluated against A549 and HepG2 cells via MTT assay, using taxol and sorafenib as positive controls. Among them, vibsanolid B **241** and C **242** resulted active against both cell lines; particularly, vibsanolid B had a significant cytotoxic activity against A549 cells, with an IC_50_ value of 1.11 μM. Further staining experiments indicated that **241** could promote apoptosis induction, enhance reactive oxygen species (ROS) level, and attenuate mitochondrial membrane potential (MMP) in A549 cells [149] (Figure 95).

### 5.3. Other Skeletons

**Figure 96:**
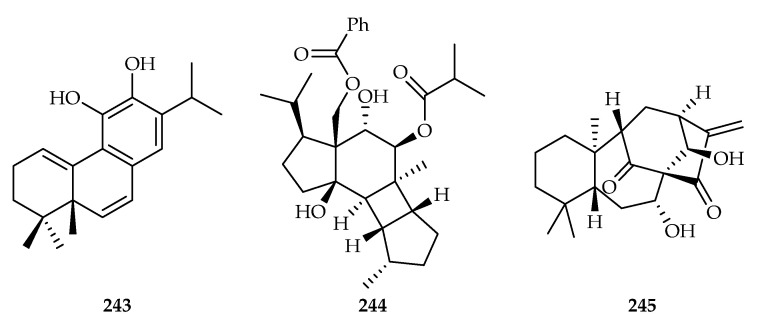
Structures of prattinin A **243** [150], vulgarisin B **244** [151], and crokonoid A **245** [152].

Prattinin A **243**, is a new diterpenoid with a rearranged skeleton, isolated by methanolic extraction of the roots of *Salvia prattii* (Lamiaceae) [150] (Figure 96). The isolation of the new compound was possible after fractionation of the crude extract by silica gel, ODS column chromatography, and the subsequent purification of the fractions by reverse-phase (RP) HPLC. Its structure was established by extensive NMR analyses, and its absolute configuration was elucidated by comparing the calculated and experimental electronic circular dichroism (ECD) spectra. Prattinin A **243** showed significant activity for HL-60 cells, with an IC_50_ value of 2.6 μM, using mitomycin C as a positive control.

Three new diterpenoids, vulgarisins B–D were isolated by ethanolic extraction from the whole plant of *Prunella vulgaris* Linn. (Labiatae), an annual to perennial herb widely used for treatment of human tuberculosis, infectious hepatitis, thyroid gland swell, hypertension, and bacillary dysentery, as well as high blood pressure [151]. These diterpenoids contain a rare 5/6/4/5 fused tetracyclic ring skeleton, and their structures were determined through the analysis of spectroscopic data, while the absolute configurations were assigned based on single crystal X-ray diffraction, as well as CD analysis. Vulgarisin B **244** demonstrated modest cytotoxic activity against the A549 cell line, with IC_50_ values of 18.0 μM, using the MTT method and 5-FU as the positive control (Figure 96).

Crokonoid A **245**, a highly rearranged diterpenoid with a new 6/7/6/5-fused tetracyclic carbon skeleton featuring a dual-bridged tricyclo [4.4.1.1^1,4^]dodecane-2,11-dione ring system, was isolated by ethanolic extraction and subsequent partitioning in water and ethyl acetate from *Croton kongensis* (Euphorbiaceae) aerial parts [152]. Compound **245** exhibited significant cytotoxicity against the HL-60 and A549 cell lines, with IC_50_ values of 1.24 and 1.92 μM, respectively (Figure 96).

**Figure 97:**
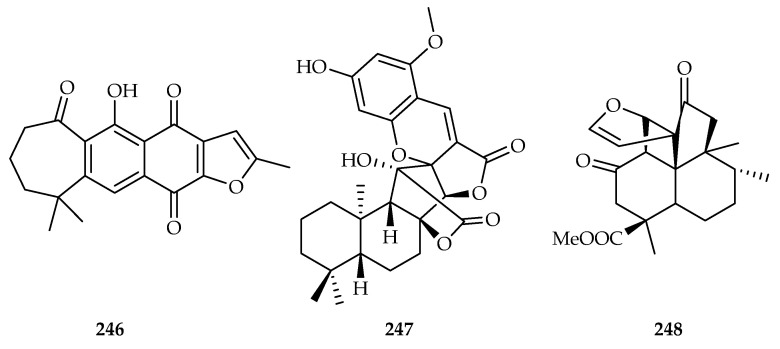
Structures of aureoglandulosin A **246** [153], fischeriana A **247** [154], and cracroson D **248** [43].

Seven new diterpenoids were isolated by ethanolic extraction and subsequent partitioning in CH_2_Cl_2_ from *Caryopteris aureoglandulosa* (Van.) C. Y. Wu (Verbenaceae) (whole plant) [153]. These diterpenoids were structurally determined by HRESIMS and NMR spectroscopic analyses, single-crystal X-ray diffraction, and ECD data. The new compounds were tested against five types of lung cancer cells (H1650, PC-9, 95-D, A549, and H460) by the CCK-8 method; among them, aureoglandulosin A **246** exhibited significant cytotoxic activity against the PC-9, H1650, and A549 cell lines, with IC_50_ values of 8.2, 5.4, and 10.4 μM respectively, using 5-FU as the positive control. Structurally, aureoglandulosin A **246** is a highly oxygenated abietane diterpenoid, with an unprecedented 7/6/6/5-ring system (Figure 97).

A novel meroterpenoid with a rare carbon skeleton, named fischeriana A **247**, was isolated from the roots of *Euphorbia fischeriana* (Euphorbiaceae), which is widely distributed in northeast China. Fischeriana A **247** possesses an unusual heptacyclic ring system (6/6/5/5/5/6/6), featuring a modified *ent*-abietane diterpene, combined with a phloroglucinol moiety by direct carbon–carbon linkage (Figure 97). In the in vitro bioactivity assays, fischeriana A **247** showed marked anti-tumor activity against the HepG2 cell line, with an IC_50_ value of 15.75 μM, using cisplatin as a positive control [154].

One new diterpenoid, named cracroson D **248**, with an undescribed carbon skeleton, was isolated by methanolic extraction of *Croton crassifolius* Geisel. (Euphorbiaceae) roots, together with three clerodane diterpenoids (see Section 3.1.1) [43]. Compound **248** was evaluated in vitro for cytotoxicity against the T24 and A549 cells using the CCK-8 method, and it showed moderate activity, with an IC_50_ value of 14.48 μM against T24, using gemcitabine as a positive control (Figure 97).

**Figure 98:**
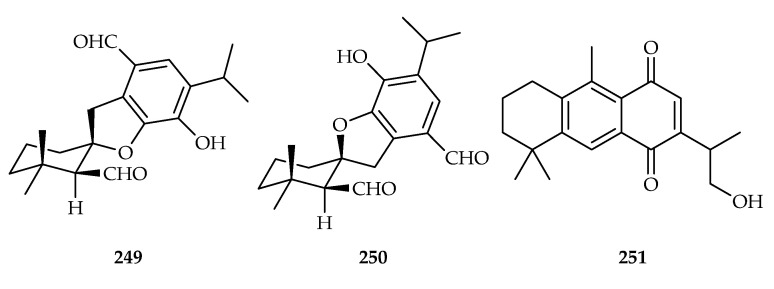
Structures of spirodesertols A **249** [102] and B **250** [102] and tebesinone B **251** [155].

Two highly modified spirocyclic diterpenoids, with an unprecedented 6-isopropyl-3*H*-spiro[benzofuran-2,1′-cyclohexane] motif, spirodesertols A **249** and B **250**, together with four new icetexane diterpenoids, salviadenones A–D (see Section 3.2.4), were isolated from the root extract of *Salvia deserta* [102] (Figure 98). Their structures were elucidated by spectroscopic analysis, quantum chemical calculations (including the ^13^C chemical shift calculations with DP4+ probability analysis and electronic circular dichroism (ECD) calculations), and single-crystal X-ray diffraction analysis. The two spirodesertols **249** and **250** exhibited cytotoxicity against the five cancer cell lines considered (A-549, SMMC-7721, HL-60, MCF-7, and SW-480), with IC_50_ values in the range of 6.60–21.65 μM; particularly, spirodesertol **249** was more cytotoxic than the positive control, cisplatin. The MTS method was used for cytotoxic analysis.

Phytochemical investigation of the root extracts of *Salvia tebesana* Bunge (Lamiaceae), an endemic plant of Iran, led to the isolation of tebesinone B **251** (Figure 98), which was examined for cytotoxicity on the MCF-7, B16F10 (melanoma), PC-3, and C26 (colon cancer) cells lines, with IC_50_ values of 7.56, 10.34, 5.47, and 5.48 μM, respectively [155].

**Table 1:** List of compounds with cytotoxic activity < 20 μM (in red, IC_50_ values better than the positive control).

Skeleton	Comp.	IC_50_ (<20 μM)	Cell line	Positive Control (IC_50_ μM)	Ref.
Monocyclic diterpenes	**1**	13.9, 19.3	ACHN, HeLa,	Vinblastin (28.0, 0.01)	[5]
	**2**	10.3, 1.6, 9.2, 11.3	ACHN, HeLa, SMMC-7721, MCF-7	Vinblastin (28.0, 0.01, 2.85, 7.5)	[5]
	3	9.39, 4.68, 4.24, 12.17	KB, MCF-7, A549, HCT116	DOX (0.53, 0.13, 1.12, 0.39)	[6]
	**4**	16.20, 15.41, 8.29 and 6.30	KB, MCF-7, A549, HCT116	DOX (0.53, 0.13, 1.12, 0.39)	[6]
Casbane	**5**	3, 5, 5, 4, 6, 7	HeLa, HT29, MCF-7, MM96L, K562, NFF	-	[8]
	**6**	6, 5, 3, 4, 6, 6	HeLa, HT29, MCF-7, MM96L, K562, NFF	-	[8]
Daphnane	**7**	3.0, 6.5	SW620, RKO	5-FU (10, >10)	[10]
	**8**	15.76, 18.30	A549, MCF-7	DDP (5.33), DOX (0.24)	[11]
	**9**	9.67, 10.44, 15.57	A549, Hep3B, MCF-7	DDP (5.33), sorafenib (11.27), DOX (0.24)	[11]
	**10**	9.14, 7.83, 16.55	A549, Hep3B, MCF-7	DDP (5.33), sorafenib (11.27), DOX (0.24)	[11]
	**11**	6.84	Hep3B	Sorafenib (11.27)	[11]
	**12**	13.42	HepG2	Oxaliplatin (24.26)	[12]
	**13**	17.59, 15.59, 14.99, 13.24	HepG2, HL-60, K562, HeLa	DDP (7.12, 10.31, 5.63, 1.74)	[13]
	**14**	11.23, 18.34, 12.82, 17.82, 5.31	HepG2, A375, HL-60, K562, HeLa	DDP (7.12, 3.45, 10.31, 5.63, 1.74)	[13]
Fusicoccane	**15**	18.0	HeLa	Etoposide (4.0)	[14]
Ingenane	**16**	14.20, 6.19	HepG2, DU145	-	[16]
	**17**	15.82, 9.27	MCF-7, DU145	-	[16]
	**18**	10.26	MCF-7	-	[16]
	**19**	15.37, 15.62	A375, HMCB	-	[17]
	**20**	12.6, 11.2, 5.05, 4.72, 12.2, 11.5, 2.28, 3.83, 5.23	HepG2, HepG2/DOX, HCT-15, HCT-15/5-FU, A549, A549/CDDP, A375, RKO, MDA-MB-231	DOX (0.49, 6.41, 0.77, 3.94, 0.78, 4.25, 1.32, 0.93, 0.26)	[18]
	**21**	5.92	MV4-11	Taxol (0.055)	[19]
	**22**	9.8	HeLa	Parthenolide (2.1)	[20]
Jatrophane	**23**	17.63, 10.97, 15.49	NCI-H460, U87, U87-TxR	-	[21]
	**24**	9.9	HeLa	Parthenolide (2.1)	[20]
	**25**	20.19, 7.21	HepG2, DU145	-	[16]
	**26**	15.25, 13.24, 7.24	MCF-7, HepG2, DU145	-	[16]
	**27**	11.25, 9.47, 8.29	MCF-7, HepG2, DU145	-	[16]
	**28**	6.29, 10.07, 4.19	MCF-7, HepG2, DU145	-	[16]
Latyrane	**29**	8.08, 14.64	Saos-2, MG-63	5-FU (19.01, 25.00)	[22]
	**30**	18.4, 15.8	A549, HeLa	DOX (1.23, 0.52)	[23]
	**31**	12.5	A549	DOX (0.52)	[23]
	**32**	11.3	C4-2B	DOX (0.23)	[24]
	**33**	12.29	MV4-11	Taxol (0.055)	[19]
	**34**	14.80	MV4-11	Taxol (0.055)	[19]
	**35**	9.43, 13.22	MCF-7, HepG2	DDP (16.55, 2.29)	[25]
Pepluane	**36**	5.8	HeLa	Parthenolide (2.1)	[20]
Rhamnofolane	**37**	8.21, 6.71, 13.8, 19.1	A549, MDAMB231, HepG2, HepG2-DOX	DOX (1.23, 0.34, 0.76, 30.5)	[23]
	**38**	2.69, 4.22, 6.44, 4.20, 5.80	A549, HeLa, MDAMB231, HepG2, HepG2-DOX	DOX (1.23, 0.52, 0.34, 0.76, 30.5)	[23]
Tigliane	**39**	3.7	A549	ADR (0.4)	[28]
	**40**	4.6, 18.9	A549, HL-60	ADR (0.4, 0.08)	[28]
	**41**	4.1	A549	ADR (0.4)	[28]
	**42**	0.9	A549	ADR (0.4)	[28]
	**43**	1.3, 2.4	A549, HL-60	ADR (0.4, 0.08)	[28]
	**44**	0.20, 17.60	K562, MCF-7	Paclitaxel (3.67, 7.56)	[29]
	**45**	0.21	K562	Paclitaxel (3.67)	[29]
Taxane	**46**	4.9	HeLa	Paclitaxel (0.0014)	[30]
Clerodane	**47**	4.7, 5.1, 4.9, 5.0, 4.9	A549, MDA-MB-231, MCF-7, KB, KB-VIN	Paclitaxel (6.2, 8.8, 10.4, 6.3, 1926.0)	[32]
	**48**	4.6, 4.8, 4.8, 4.8, 4.9	A549, MDA-MB-231, MCF-7, KB, KB-VIN	Paclitaxel (6.2, 8.8, 10.4, 6.3, 1926.0)	[32]
	**49**	4.8, 5.1, 5.2, 4.9, 4.8	A549, MDA-MB-231, MCF-7, KB, KB-VIN	Paclitaxel (6.2, 8.8, 10.4, 6.3, 1926.0)	[32]
	**50**	10.0, 6.4, 11.0, 9.9, 11.4	A549, MDA-MB-231, MCF-7, KB, KB-VIN	Paclitaxel (5.8, 6.4, 8.5, 5.0, 1421.9)	[33]
	**51**	12.84, 12.43, 9.46, 11.21, 11.21	BT474, Chago-K1, HepG2, KATO-III, SW-620	DOX (0.64, 0.47, 0.07, 0.85, 0.10)	[34]
	**52**	10.4, 15.3	A549, HL-60	DDP (7.8, 3.4)	[35]
	**53**	17.91	Hep3B	5-FU (10.53)	[36]
	**54**	4.4	A549	DDP (-)	[37]
	**55**	4.6	A549	DDP (-)	[37]
	**56**	19.0, 15.8	A549, HeLa	Etoposide (12.5, 36.1)	[38]
	**57**	8.7, 5.3, 8.1	A549, HeLa, HepG2	Etoposide (12.5, 36.1, 2.5)	[38]
	**58**	17.9	HeLa	Etoposide (25.8)	[39]
	**59**	9.7, 10.9, 12.4, 7.2	HepG2, A549, HeLa, K562	Etoposide (16.0, 10.4, 36.1, 17.9)	[40]
	**60**	19.7, 12.1	A549, HeLa	Etoposide (16.5, 25.8)	[41]
	**61**	18.3, 9.0	A549, HeLa	Etoposide (16.5, 25.8)	[41]
	**62**	10.2, 5.3, 10.7	A549, HeLa, HepG2	Etoposide (16.5, 25.8, 16.0)	[41]
	**63**	1.9, 4.6	A549, HepG2	Homoharringtonine (0.0316, 0.0345)	[42]
	**64**	12.26	T24	Gemcitabine (6.50)	[43]
	**65**	11.6, 7.1, 9.3	HeLa, PANC-1, A549	Etoposide (3.8, 5.2, 9.9)	[44]
	**66**	9.4, 5.6, 6.8	HeLa, PANC-1, A549	Etoposide (3.8, 5.2, 9.9)	[44]
	**67**	17.2, 9.8, 12.5	HeLa, PANC-1, A549	Etoposide (3.8, 5.2, 9.9)	[44]
	**68**	5.6	ECC-1	-	[45]
	**69**	17.9	SGC-7901	DDP (16.7)	[46]
	**70**	19.7	HepG2	DOX (0.38)	[47]
	**71**	10	MDA MB 231	-	[48]
	**72**	7.8	MDA MB 231	-	[48]
	**73**	0.66, 0.48, 0.68, 0.56, 0.98	A549, MDA-MB-231, MCF-7, KB, KB-VIN	Paclitaxel (0.0065, 0.0084, 0.0121, 0.0071, 2.21)	[49]
	**74**	0.47, 0.49, 0.50, 0.45, 0.49	A549, MDA-MB-231, MCF-7, KB, KB-VIN	Paclitaxel (0.0065, 0.0084, 0.0121, 0.0071, 2.21)	[49]
	**75**	4.60, 4.95, 4.94, 5.19, 4.92	A549, MDA-MB-231, MCF-7, KB, KB-VIN	Paclitaxel (0.0065, 0.0084, 0.0121, 0.0071, 2.21)	[49]
	**76**	5.04, 4.90, 5.82, 5.23, 5.19	A549, MDA-MB-231, MCF-7, KB, KB-VIN	Paclitaxel (0.0065, 0.0084, 0.0121, 0.0071, 2.21)	[49]
	**77**	4.75, 3.31, 4.65, 4.25, 4.76	A549, MDA-MB-231, MCF-7, KB, KB-VIN	Paclitaxel (0.0065, 0.0084, 0.0121, 0.0071, 2.21)	[49]
	**78**	5.98, 4.93, 6.39, 5.16, 5.03	A549, MDA-MB-231, MCF-7, KB, KB-VIN	Paclitaxel (0.0065, 0.0084, 0.0121, 0.0071, 2.21)	[49]
	**79**	2.29, 0.49, 0.69, 0.56, 0.61	A549, MDA-MB-231, MCF-7, KB, KB-VIN	Paclitaxel (0.0065, 0.0084, 0.0121, 0.0071, 2.21)	[49]
	**80**	4.76, 4.73, 5.19, 4.74, 4.88	A549, MDA-MB-231, MCF-7, KB, KB-VIN	Paclitaxel (0.0065, 0.0084, 0.0121, 0.0071, 2.21)	[49]
	**81**	8, 10.4, 14.8	HCT-116, PC-3, MDA-MB-435	5-FU (52), Mitomycin-C (63)	[50]
Halimane	**82**	5.2, 11.8	A549, HL-60	DDP (7.8, 3.4)	[51]
Labdane	**83**	4.4	theophylline-stimulated murine B16 melanoma 4A5 cells	Arbutin (174)	[52]
	**84**	8.6	theophylline-stimulated murine B16 melanoma 4A5 cells	Arbutin (174)	[52]
	**85**	4.6	theophylline-stimulated murine B16 melanoma 4A5 cells	Arbutin (174)	[52]
	**86**	2.22	L5178Y	Kahalalide F (4.30)	[53]
	**87**	1.42	L5178Y	Kahalalide F (4.30)	[53]
	**88**	12.9	L5178Y	Kahalalide F (4.30)	[53]
	**89**	17.7	p388D1	Taxol (0.03)	[54]
	**90**	1.6	MDA-MB231	Paclitaxel (0.003)	[55]
	**91**	1.5	MDA-MB231	Paclitaxel (0.003)	[55]
	**92**	7.5	MCF-7	DDP (22.2)	[56]
	**93**	8.0, 19.7	HepG2, XWLC-05	DDP (3.0, 4.3)	[57]
	**94**	11.7, 17.6	ACP01, A549	DOX (8.3, 3.1)	[58]
	**95**	2.8	HL-60	ADR (0.02)	[59]
	**96**	13.49	A549	Paclitaxel (6.2)	[60]
	**97**	5.92	U937	Paclitaxel (0.001)	[61]
	**98**	10.9, 14.6, 18.2	MCF-7, MDA-MB231, A549	SAHA (14.2, 6.91, 3.85)	[62]
	**99**	7.63, 13.5, 15.3	MCF-7, MDA-MB231, A549	SAHA (14.2, 6.91, 3.85)	[62]
Abietane	**100**	5.88, 11.74	A2780, HepG2	Taxol (0.006, 0.003)	[63]
	**101**	0.37, 7.85, 1.45, 8.72, 0.23	HepG2, NB4, HeLa, MCF-7, HL-60	DDP (0.2, 0.03, 0.05, 0.12, 0.18)	[64]
	**102**	1.27, 1.02, 0.35, 0.96, 5.17	NB4, HeLa, K562, MCF-7, HL-60	DDP (0.03, 0.05, 0.2, 0.12, 0.18)	[64]
	**103**	9.4–20.4	HL-60, SMMC-7721, A549, MCF-7, SW480	DDP (1.9–18.3)	[65]
	**104**	6.3, 12.7, 7.9	LUPF045, OVPF038, OVPF008	Paclitaxel (11.2, 1.4, 1.1)	[66]
	**105**	9.0, 11.7, 19.3, 16.8	LUPF045, LUPF003, OVPF038, OVPF008	Paclitaxel (11.2, 11.0, 1.4, 1.1)	[66]
	**106**	9.65	CaCo2	ADR (0.348)	[67]
	**107**	17.86	CaCo2	ADR (0.348)	[67]
	**108**	9.18, 9.70, 18.3, 16.2	C4-2B, C4-2B/ENZR, HCT-15, RKO	DOX (0.12, 0.34, 0.56, 0.87)	[68]
	**109**	13.4, 11.1	C4-2B, C4-2B/ENZR	DOX (0.12, 0.34)	[68]
	**110**	17.7, 15.2	C4-2B, C4-2B/ENZR,	DOX (0.12, 0.34)	[68]
	**111**	9.23, 15.1	C4-2B, C4-2B/ENZR,	DOX (0.12, 0.34)	[68]
	**112**	7.39, 9.20, 19.0	C4-2B, C4-2B/ENZR, HCT-15	DOX (0.12, 0.34, 0.56)	[68]
	**113**	12.10, 15.95	MM-231, Hep3B	DDP (3.82, 2.97)	[70]
	**114**	9.12, 8.50	MM-231, Hep3B	DDP (3.82, 2.97)	[70]
	**115**	15.47	HeLa	DDP (11.34)	[71]
	**116**	3.75, 9.31	HeLa, MCF-7	DDP (11.34, 25.14)	[71]
	**117**	4.6, 11.5, 16.4	HeLa, H460, Namalwa	DOX (8.2, 0.7, 70.1)	[72]
	**118**	9.5, 17.4, 13.3	HeLa, H460, Namalwa	DOX (8.2, 0.7, 70.1)	[72]
	**119**	15.7, 19.8, 13.2	HL-60, MCF-7, SW-480	DDP (2.2, 10.5, 12.7)	[73]
	**120**	15.6, 17.4, 17.0, 16.6, 10.1	HL-60, SMMC-7721, A549, MCF-7, SW480	DDP (2.2, 17.3, 15.6, 10.5, 12.7)	[73]
	**121**	0.58, 1.36, 5.82, 2.06, 4.21	HL-60, SMMC-7721, A549, MCF-7, SW-480	DDP (1.56, 12.32, 15.34, 20.58, 24.26)	[74]
	**122**	0.82, 2.65, 5.64, 6.26, 8.53	HL-60, SMMC-7721, A549, MCF-7, SW-480	DDP (1.49, 13.32, 11.54, 24.59, 28.32)	[75]
	**123**	9.5, 10.7	MCF-7, PANC-1	Paclitaxel (0.65), gemcitabine (0.059)	[76]
	**124**	9.8, 10.3	MCF-7, PANC-1	Paclitaxel (0.65), gemcitabine (0.059)	[76]
	**125**	17.9	MCF-7	5-FU (3.6)	[77]
	**126**	4.3, 2.8, 4.5	MDA-MB-231, MCF-7, HeLa	DOX (0.48, 0.36, 0.82)	[78]
	**127**	20.02	MDA-MB-231	DDP (20.27)	[79]
	**128**	7.9	MOLT-4	DDP (2.7)	[80]
	**129**	10.70	A549	-	[81]
	**130**	5.89	A549	DDP (20.66)	[82]
	**131**	6.94	A549	DDP (20.66)	[82]
	**132**	16.6, 17.4	KB, MCF7	Paclitaxel (0.0031, 0.008)	[83]
	**133**	13.71	A549	DDP (15.27)	[84]
	**134**	10.91, 18.42	HL-60, A549	DDP (11.70, 15.27)	[84]
	**135**	10.75	HL-60	ADR (0.019)	[85]
	**136**	10.58	HL-60	ADR (0.019)	[85]
	**137**	2.86, 5.03	A549, HL-60	ADR (0.852, 0.019)	[85]
	**138**	1.00, 1.36	A549, HL-60	ADR (0.852, 0.019)	[85]
	**139**	9.9	HL-60	ADR (0.02)	[86]
	**140**	3.8, 2.8	A549, HL-60	ADR (0.85, 0.02)	[86]
	**141**	9.0, 2.5	A549, HL-60	ADR (0.85, 0.02)	[86]
	**142**	19.28	SW1990	Paclitaxel (92.16)	[87]
	**143**	9.91	SW1990	Paclitaxel (92.16)	[87]
	**144**	17.6	MM-CSCs	Bortezomib (0.008)	[88]
	**145**	2.63, 2.52, 4.84, 1.18, 3.23	HL-60, SMMC-7721, A549, MCF-7, SW480	DDP (1.81, 8.86, 11.68, 15.92, 8.86)	[89]
	**146**	17.34	MSTO-211H	Staurosporine (0.0011)	[90]
	**147**	2.6	L6	Podophyllotoxin (0.02)	[91]
	**148**	15.32, 8.36	CCRF-CEM, CEM/ADR5000	DOX (0.01, 66.83)	[92]
Cassane	**149**	1.4, 1.1, 1.2	A549, NCI-H1975, NCI-H1299	Camptothecin (0.7, 1.2, 0.8)	[94]
	**150**	0.5, 0.4, 0.9	A549, NCI-H1975, NCI-H1299	Camptothecin (0.7, 1.2, 0.8)	[94]
	**151**	11.42	HeLa	DDP (1.65)	[95]
	**152**	3.2	HEL	DOX (0.12)	[96]
Dolabrane	**153**	8.97, 8.97, 4.62, 17.11, 3.93	MDA-MB-453, MDA-MB-231, SK-BR-3, ZR-75-1, MT-1	DDP (4.37, 6.25, 8.42, 20.65, 7.90)	[99]
	**154**	18.06, 17.24, 8.07	MDA-MB-453, SK-BR-3, MT-1	DDP (4.37, 8.42, 7.90)	[99]
	**155**	1.73, 8.12, 2.45, 12.03, 3.75, 1.97	MD-MBA-453, MD-MBA-231, SK-BR-3, MCF-7, MT-1, ZR-75-1	DDP (4.37, 3.73, 8.42, 3.21, 7.90, 20.65)	[100]
Icetexane	**156**	1.4, 0.82	U-251, SKLU-1	ADR (0.08, 0.05)	[101]
	**157**	17.70	HL-60	DDP (2.47)	[102]
Pimarane	**158**	3.75	MT-1	DDP (7.90)	[99]
	**159**	0.27	MB-MDA-231	2-morpholin-4-yl-8-phenylchromen-4-on (0.38)	[103]
	**160**	2.6	A549	-	[104]
	**161**	0.35, 0.23, 1.42	HepG2, HL60, HeLa	DDP (0.17, 0.21, 0.06)	[105]
	**162**	4.29, 1.55, 1.27	HepG2, HL60, HeLa	DDP (0.17, 0.21, 0.06)	[105]
	**163**	14.327, 12.033	MCF-7, A549	DOX (15.7, 4.2)	[106]
Podocarpane	**164**	13.2	SW480	DDP (12.8)	[107]
Staminane	**165**	12.31, 16.36, 5.62, 14.70, 15.24	HL-60, A549, SMMC-7721, MCF-7, SW-480	DDP (3.19, 23.25, 22.53, 19.56, 25.57)	[108]
	**166**	15.76, 19.15, 19.84	HL-60, SMMC-7721, SW-480	DDP (3.19, 22.53, 25.57)	[108]
Atisane	**167**	4.1, 4.0	A549, HL-60	ADR (0.85, 0.02)	[86]
Beyerane	**168**	11.1	SKOV3	DDP (-)	[109]
Cephalotane	**169**	4.5, 6.8	A549, HL-60	ADR (0.44, 0.075)	[111]
	**170**	2.4, 2.2	A549, HL-60	ADR (0.44, 0.075)	[111]
	**171**	1.0, 9.8	A549, HL-60	ADR (0.44, 0.075)	[111]
	**172**	0.77, 1.129	HL60, A549	DOX (0.031, 0.124)	[112]
	**173**	1.63, 1.72, 1.73	A549, HeLa, SGC7901	DDP (7.32, 8.42, 11.43)	[113]
	**174**	4.64, 6.73, 3.84	A549, HeLa, SGC7901	DDP (7.32, 8.42, 11.43)	[113]
	**175**	0.10, 0.13, 0.14	A549, HeLa, SGC7901	DDP (7.32, 8.42, 11.43)	[113]
	**176**	0.31, 0.71, 0.35	A549, HeLa, SGC7901	DDP (7.32, 8.42, 11.43)	[113]
	**177**	4.92, 6.85, 5.88	A549, HeLa, SGC7901	DDP (7.32, 8.42, 11.43)	[113]
	**178**	16.5, 18.3	A549, HeLa	DDP (7.32, 8.42)	[113]
	**179**	19.7	HeLa	DDP (8.42)	[113]
	**180**	14.6, 15.3	A549, HeLa	DDP (7.32, 8.42)	[113]
	**181**	16.6	HeLa	DDP (8.42)	[113]
Kaurane	**182**	13.3	HMy2.CIR	-	[119]
	**183**	1.0, 1.5, 4.4, 2.9, 0.9	HL-60, SMMC-7721, A549, MCF-7 SW-480	DDP (3.0, 10.2, 16.0, 15.3, 9.3)	[120]
	**184**	7.0, 4.7, 19.6, 11.0, 2.5	HL-60, SMMC-7721, A549, MCF-7, SW-480	DDP (3.0, 10.2, 16.0, 15.3, 9.3)	[120]
	**185**	1.2, 5.3, 3.0, 2.9, 0.8	HL-60, A549, SMMC-7721, MCF-7, SW-480	DDP (2.1, 14.7, 5.7, 15.3, 9.2)	[121]
	**186**	3.4, 8.6, 4.1, 2.1	HL-60, SMMC-7721, MCF-7, SW-480	DDP (2.1, 5.7, 15.3, 9.2)	[121]
	**187**	1.0, 5.8, 3.2, 3.4, 1.9	HL-60, A549, SMMC-7721, MCF-7, SW-480	DDP (2.1, 14.7, 5.7, 15.3, 9.2)	[121]
	**188**	5.92, 15.31, 17.27	SKOV3, SK-MEL-2, HCT15	Etoposide (-)	[122]
	**189**	16.94	HepG2	DDP (22.19)	[123]
	**190**	12.38	HepG2	DDP (22.19)	[123]
	**191**	8.7, 11.3	SKOV3, MCF-7	DDP (-)	[109]
	**192**	8.1	Hep-3B	5-FU (9.36)	[124]
	**193**	5.1, 9.8, 6.8, 2.9, 4.1, 1.9	HeLa, A549, MDA-MB-231, SKOV3, Huh-7, HCT-116	Paclitaxel (0.004, 0.005, 0.003, 0.002, 0.005, 0.006)	[55]
	**194**	17.28, 11.56	SMMC-7721, HepG2	-	[125]
	**195**	4.84, 1.89, 12.10, 13.54, 8.02	HL-60, SMMC-7721, A549, MCF-7, SW-480	DDP (2.06, 11.73, 9.09, 13.93, 7.79)	[126]
	**196**	17.91	SW-480	DDP (7.79)	[126]
	**197**	14.94, 14.06, 13.78, 13.95, 3.26	HL-60, SMMC-7721, A549, MCF-7, SW-480	DDP (2.06, 11.73, 9.09, 13.93, 7.79)	[126]
	**198**	14.91	SW-480	DDP (7.79)	[126]
	**199**	5.61, 2.20, 3.33, 4.45, 2.59	HL-60, SMMC-7721, A549, MCF-7, SW-480	DDP (2.06, 11.73, 9.09, 13.93, 7.79)	[126]
	**200**	6.03, 2.58, 5.71, 3.96, 3.40	HL-60, SMMC-7721, A549, MCF-7, SW-480	DDP (2.06, 11.73, 9.09, 13.93, 7.79)	[126]
	**201**	1.27, 5.74	HL-60, A549	ADR (0.06, 0.50)	[127]
	**202**	0.47, 3.25	HL-60, A549	ADR (0.06, 0.50)	[127]
	**203**	0.58	HL-60	ADR (0.06)	[127]
	**204**	1.34, 1.07, 3.60, 2.35, 2.53	HCT-116, HepG2, BGC-823, NCI-H1650, A-2780	DDP (7.81, >10, 8.56, >10, 8.65)	[128]
	**205**	19.5, 19.6	SNU638, HCT116	Etoposide (3.10, 7.94)	[129]
	**206**	15.18, 7.22, 5.57, 1.99	MDA-MB-231, SK-Hep-1 SNU638, HCT116	Etoposide (28.56, 0.83, 3.10, 7.94)	[129]
	**207**	1.99, 2.97, 1.11, 1.51	A549, KB, MCF7, HCT116	Paclitaxel (0.011, 0.0031, 0.0083, 0.0069)	[83]
	**208**	17.36, 12.08, 12.47, 9.10, 2.66	A549, Hep-3B, PC-3, HT29, U937	CPT-11 (15.26, 23.21, 31.03, 15.11, 4.95)	[130]
	**209**	7.39, 7.06, 4.19, 2.78, 1.97	A549, Hep-3B, PC-3, HT29, U937	CPT-11 (15.26, 23.21, 31.03, 15.11, 4.95)	[130]
	**210**	17.12, 17.08, 16.49, 14.18, 6.73	A549, Hep-3B, PC-3, HT29, U937	CPT-11 (15.26, 23.21, 31.03, 15.11, 4.95)	[130]
	**211**	19.39, 19.86, 15.96, 12.64, 8.25	A549, Hep-3B, PC-3, HT29, U937	CPT-11 (15.26, 23.21, 31.03, 15.11, 4.95)	[130]
	**212**	3.56, 14.60, 3.50, 10.23, 2.96, 3.28, 2.07, 4.04	HeLa, SK-OV-3, SK-HEP-1, Caco2, MDA-MB-231, PC-3, SW480, A549/Taxol	DDP (23.12, 26.98, 8.37, 6.53, 18.54, 16.76, 22.80, 10.22)	[131]
	**213**	9.92, 13.37, 10.2, 16.12	HeLa, SW480, A549, ACHN	CDDP (16.2, 29.93, NA, 20.36)	[132]
	**214**	8.27, 10.37	HeLa, SW480	CDDP (16.2, 29.93)	[132]
	**215**	12.48, 14.20, 16.04, 14.65, 3.74	HL-60, SMMC-7721, A-549, MCF-7, SW-480	DDP (2.06, 11.73, 9.09, 13.93, 7.79)	[133]
	**216**	5.53, 2.69, 6.23, 3.86, 3.17	HL-60, SMMC-7721, A-549, MCF-7, SW-480	DDP (2.06, 11.73, 9.09, 13.93, 7.79)	[133]
	**217**	18.04, 12.28, 14.50, 15.95, 9.68	HL-60, SMMC-7721, A-549, MCF-7, SW-480	DDP (2.06, 11.73, 9.09, 13.93, 7.79)	[133]
	**218**	3.77, 3.99, 6.80, 3.20, 1.23	HL-60, SMMC-7721, A-549, MCF-7, SW-480	DDP (2.06, 11.73, 9.09, 13.93, 7.79)	[133]
	**219**	6.6, 2.3, 11.5	MCF-7, A549, PC-3	ADR (0.4, 0.1, 0.8)	[134]
	**220**	15.81, 1.93	A549, K562	ADR (3.48, 3.49)	[135]
	**221**	9.89, 0.59	A549, K562	ADR (3.48, 3.49)	[135]
	**222**	5.11	A549	Etoposide (36.5)	[136]
	**223**	2.8, 3.4, 3.6, 4.0, 2.6	HL-60, SMMC-7721, A549, MCF-7, SW480	DDP (3.0, 10.2, 16.0, 15.3, 9.3)	[137]
	**224**	3.9, 2.4, 4.2	EC109, SHG-44, MCF-7	Adenanthin (6.5, 4.8, 7.6)	[138]
	**225**	15.83, 17.37, 19.47, 19.50	HepG2, Caco2, U2OS, MDA-MB-231	-	[139]
	**226**	15.45, 10.05, 3.01, 3.38	A549, HCT116, CCRF-CEM, HL-60	DOX (0.20, 0.070, 0.014, 0.01)	[140]
	**227**	11.60, 8.64, 2.77, 3.16	A549, HCT116, CCRF-CEM, HL-60	DOX (0.20, 0.070, 0.014, 0.01)	[140]
	**228**	15.59	EC109	-	[141]
	**229**	4.07	HCT116	-	[142]
Prenyleudesmane	**230**	13.8	HeLa	Etoposide (21.2)	[143]
Norditerpenoids and Dinorditerpenoids	**231**	3.8, 6.4	A-2780, HEY	Paclitaxel (0.16, 0.10)	[144]
	**232**	<2.5	A-2780, HEY	Paclitaxel (0.16, 0.10)	[144]
	**233**	5.8	A549	DOX (0.090)	[145]
	**234**	0.7	U87-MG	DOX (0.0996)	[145]
	**235**	0.87, 0.38, 4.23, 19.17	HeLa, AGS, MDA-MB-231, HepG2	DDP (6.30, 20.98, 16.26, 24.07)	[146]
	**236**	11.61, 0.88, 5.46, 5.56, 1.35	HeLa, AGS, MDA-MB-231, HepG2	DDP (6.30, 20.98, 16.26, 24.07, 18.27)	[146]
Vibsane-type diterpenoid	**237**	2.88, 7.31, 6.89, 5.88, 5.22	HL60, A549, SMMC-7721, MCF7, SW480	DDP (5.77, 19.68, 22.32, 21.17, 29.21)	[148]
	**238**	14.45, 18.47	HL60, SMMC-7721	DDP (5.77, 22.32)	[148]
	**239**	2.56, 17.36, 3.42, 14.61, 5.34	HL60, A549, SMMC-7721, MCF7, SW480	DDP (5.77, 19.68, 22.32, 21.17, 29.21)	[148]
	**240**	13.09, 16.51, 19.46, 15.98	HL60, SMMC-7721, MCF7, SW480	DDP (5.77, 22.32, 21.17, 29.21)	[148]
	**241**	1.11, 13.62	A549, HepG2	Taxol (0.024), sorafenib (0.49)	[149]
	**242**	19.75, 12.61	A549, HepG2	Taxol (0.024), sorafenib (0.49)	[149]
Other skeletons	**243**	2.6	HL-60	mitomycin C (0.19)	[150]
	**244**	18.0	A549	5-FU (9.5)	[151]
	**245**	1.24, 1.92 μM	HL-60, A549	ADR (0.06, 0.50)	[152]
	**246**	8.2, 5.4, 10.4	PC-9, H1650, A549	5-FU (>10, >10, >10)	[153]
	**247**	15.75	HepG2	DDP (16.28)	[154]
	**248**	14.48	T24	Gemcitabine (6.50)	[43]
	**249**	6.60, 7.13, 11.32, 11.50, 18.20	A549, SMMC-7721, HL-60, MCF-7, SW480	DDP (13.84, 7.82, 2.47, 13.46, 10.06)	[102]
	**250**	18.00, 13.64, 18.42, 18.21, 21.65	A549, SMMC-7721, HL-60, MCF-7, SW480	DDP (13.84, 7.82, 2.47, 13.46, 10.06)	[102]
	**251**	7.56, 10.34, 5.47, 5.48	MCF-7, B16F10, PC-3, C26	DOX (1.72, 0.68, 1.84, 0.25)	[155]

DDP: cisplatin; DOX: doxorubicin; ADR: Adriamycin.

**Table 2:** Cell lines and compounds which showed better activity than the positive control.

Organ	Cell Lines	Compound (Grouped by Cell Line Higlighted in Red)
Bladder	T24	-
Blood	CCRF-CEM, CEM/ADR5000, HEL, HL-60, HMy2.CIR, Jurkat, K562, L5178Y, MM-CSCs, MOLT-4, MV4-11, Namalwa, NB4, p388D1, U937	K562: **44, 45, 59, 220, 221**; L5178Y: **86, 87**; Namalwa: **117, 118**; HL-60: **121, 122, 134, 183, 185, 187, 223, 237, 239**; CEM/ADR5000: **148; 208**, U937: **209**
Bones	MG-63, Saos-2, U2OS	Saos-2 and MG-63: **29**
Brain	SHG-44, U87, U87-TxR, U87-MG, U-251	SHG-44: **224**
Breast	BT474, MCF-7, MDA-MB-231, MDA-MB-453, MM-231, MT-1, SK-BR-3, ZR-75-1	MCF-7: **35, 47, 48, 49, 92, 98, 99, 116, 121, 122, 145, 163, 165, 183, 184, 185, 186, 187, 195, 199, 200, 216, 223, 224, 237, 239, 240, 249**; MDA-MB-231: **47, 48, 49, 127, 159, 206, 212, 235, 236**; MDA-MB-453: **155**; MT-1: **153, 155, 158**; SK-BR-3: **153, 155**; ZR-75-1: **153, 155**
Cervix	HeLa	HeLa: **56, 57, 58, 59, 60, 61, 62, 116, 117, 173–177, 212, 213, 214, 230, 235**
Colon	C26, CaCo2, HCT-15, HCT-15/5-FU, HCT-116, HT29, LoVo, RKO, SW480, SW620	SW620 and RKO: **7**; HCT-116: **81, 204, 206**; SW480: **120, 121, 122, 145, 165, 166, 183, 184, 185, 186, 187, 197, 199, 200, 212, 213, 214, 215, 216, 218, 223, 237, 239, 240**; HT29: **208, 209, 210, 211**
Endometrium	ECC-1	-
Epithelium	A375, B16F10, B16melanoma4A5, HMCB, KB, KB-VIN, MDA-MB-435, MM96L, SK-Mel2	KB: **47, 48, 49**; KB-VIN: **47, 48, 49, 50, 73, 74, 79**; B16melanoma4A5: **83–85**
Esophage	EC, EC109, EC9706, KYSE-450,	EC109: **224**
Kidney	ACHN	ACHN: **1, 2, 213**
Liver	HepG2, HepG2/DOX, Hep3B, Huh-7, SK-Hep-1, SMMC-7721	Hep3B: **9, 10, 11, 192, 208, 209, 210, 211**; HepG2: **12, 59, 62, 189, 190, 204, 235, 236, 247;**HepG2/DOX: **37, 38**; SMMC-7721: **121, 122, 145, 165, 166, 183, 185, 187, 195, 199, 200, 216, 218, 223, 237, 238, 239, 240, 249**; SK-Hep-1: **212**
Lung	95-D, A549, A549/CDDP, A549/Taxol, Chago-K1, LUPF003, LUPF045, MSTO-211H, NCI-H1229, NCI-H1650, NCI-H1975, NCI-H460, PC-9, SKLU-1, XWLC-05	A549: **47, 48, 57, 62, 65, 66, 82, 121, 122, 130, 131, 133, 145, 150, 165, 173–177, 183, 185, 187, 199, 200, 209, 216, 218, 222, 223, 237, 239, 249**; LUPF045: **104, 105**; NCI-H1975: **149, 150**; A549/Taxol: **212**; PC-9: **246**; NCI-H1650: **204, 246**
Ovaries	A2780, HEY, OVPF008, OVPF038, SKOV3	A2780: **204**; SKOV3: **212**
Pancreas	PANC-1, SW1990	SW1990: **142, 143**
Prostate	C4-2B, C4-2B/ENZR, DU145, PC3	PC-3: **81, 208, 209, 210, 211, 212**
Stomach	ACP01, AGS, BGC-823, KATO-III, SGC7901, SNU638	SGC7901: **173–177**; BGC-823: **204**, AGS: **235, 236**

**Table 3:** Plant families and isolated compounds.

Plant Family	Species	Compound
Acanthaceae	*Hypoestes forsskaolii*	**15**
Annonaceae	*Annona squamosa*	**194**
Araucariaceae	*Araucaria bidwillii*	**86–88**
Asteraceae	*Sheareria nana*	**65–68**
	*Siegesbeckia pubescens*	**159**
	*Tagetes minuta*	**3,4**
	*Wedelia prostrata*	**189, 190**
Caprifoliaceae	*Lonicera macranthoides*	**230**
Celastraceae	*Euonymus oblongifolius*	**160, 161**
	*Salacia cochinchinensis*	**162**
	*Tripterygium hypoglaucum*	**142, 143**
	*Tripterygium regelii*	**100, 121**
Compositae	*Ligularia fischeri*	**182**
Convolvulaceae	*Pharbitis nil*	**188**
Ebenaceae	*Diospyros maritima*	**222**
Euphorbiaceae	*Croton crassifolius*	**53, 64, 82, 248**
	*Croton damayeshu*	**39–43**
	*Croton insularis*	**5,6**
	*Croton kongensis*	**201–203, 245**
	*Croton sublyratus*	**95**
	*Croton tiglium*	**44, 45**
	*Croton yanhuii*	**213, 214**
	*Ephorbia alatavica*	**163**
	*Euphorbia deflexa*	**22, 24, 36**
	*Euphorbia erythradenia*	**19**
	*Euphorbia fischeriana*	**12, 13, 14, 32, 108–118, 152, 192, 247**
	*Euphorbia helioscopia*	**123, 124**
	*Euphorbia kansuensis*	**20**
	*Euphorbia kansui*	**16–18, 25–28**
	*Euphorbia lathyrism*	**35**
	*Euphorbia neriifolia*	**139–141, 167**
	*Euphorbia nicaeensis*	**23**
	*Euphorbia pekinensis*	**97**
	*Euphorbia stracheyi*	**21, 33, 34**
	*Jatropha multifida*	**30, 31, 37, 38**
	*Jatropha podagrica*	**29**
	*Trigonostemon howii*	**122**
Fabaceae	*Caesalpinia sappan*	**151**
	*Copaifera trapezifolia*	**94**
	*Erythrophleum fordii*	**149, 150**
Flacourtiaceae	*Casearia graveolens*	**63**
	*Cesearia grewiifolia*	**51**
	*Casearia kurzii*	**54–62**
Labiateae	*Ajuga decumbens*	**70**
	*Isodon oresbius*	**212**
	*Isodon pharicus*	**215–218**
	*Perovskia atriplicifolia*	**101, 102**
	*Prunella vulgaris*	**244**
	*Salvia multicaulis*	**148**
Lamiaceae	*Ajuga ovalifolia*	**129**
	*Clerodendranthus spicatus*	**165, 166**
	*Clerodendrum bracteatum*	**133, 134**
	*Isodon excisoides*	**204**
	*Isodon forrestii*	**119, 120, 225**
	*Isodon interruptus*	**132, 207**
	*Isodon pharicus*	**195–200**
	*Isodon rubescens*	**185–187, 220, 221, 214**
	*Isodon scoparius*	**183, 184, 223**
	*Isodon serra*	**103**
	*Mesona procumbens*	**208–211**
	*Phlomoides betonicoides*	**92**
	*Plectranthus scutellarioides*	**125, 144**
	*Rabdosia japonica*	**226, 227**
	*Rabdosia rubescens*	**224, 228**
	*Salvia ballotiflora*	**156**
	*Salvia ceratophylla*	**128**
	*Salvia deserta*	**130, 131, 157, 249, 250**
	*Salvia leriifolia*	**147**
	*Salvia plebeia*	**106, 107**
	*Salvia prattii*	**243**
	*Salvia tebesana*	**251**
	*Salvia yunnanensis*	**145**
	*Scutellaria barbata*	**52, 69**
	*Vitex cofassus*	**50**
Lycopodiaceae	*Lycopodium complanatum*	**146**
Meliaceae	*Aphanamixis polystachya*	**1, 2**
Menispermaceae	*Tinospora cordifolia*	**81**
	*Tinospora crispa*	**71, 72**
Orchidaceae	*Pholidota cantonensis*	**89**
Picrodendraceae	*Austrobuxus carunculatus*	**233, 234**
Poaceae	*Zea mays*	**205, 206**
Podocarpaceae	*Podocarpus macrophyllus*	**235, 236**
	*Podocarpus nagi*	**231, 232**
Pteridaceae	*Pteris semipinnata*	**229**
Rhizophoraceae	*Ceriops tagal*	**153–155, 158**
Ranunculaceae	*Anemone hupehensis*	**164**
Salicaceae	*Laetia corymbulosa*	**47–49, 73–80**
Scapaniaceae	*Scapania carinthiaca*	**219**
Scrophulariaceae	*Scoparia dulcis*	**98, 99**
Sinoptiridaceae	*Aleuritopteris argentea*	**168, 191**
Taxaceae	*Amentotaxus argotaenia*	**90–91, 193**
	*Cephalotaxus fortunei*	**169–171, 172, 173–181**
	*Cephalotaxus lanceolata*	**173–181**
	*Taxus wallichiana*	**46**
Taxodiaceae	*Cunninghamia lanceolata*	**126**
	*Metasequoia glyptostroboides*	**127**
Thymelaeaceae	*Daphne genkwa*	**7, 8–11**
Verbenaceae	*Caryopteris aureoglandulosa*	**246**
	*Caryopteris nepetaefolia*	**104, 105**
	*Clerodendrum chinense*	**135–138**
Viburnaceae	*Viburnum odoratissimum*	**237–242**
Zingiberaceae	*Alpinia galanga*	**83–85**
	*Hedychium forrestii*	**93**
	*Roscoea purpurea*	**96**

## 6. Methodology

The references of this paper were obtained by searching in the main sources of chemistry, such as SciFinder, Scopus, and Web of Science. The research was performed until 31 May 2022 and covers the years 2017–2022. The main topic searched was “diterpen”, and the articles found were refined by year of publication, language (English), and type of document (article and communication). Since the present review regards diterpenes from plants with cytotoxic activity, they were further refined using the keyword “cytotoxic or cancer” and excluding marine, coral, fungi, alkaloid, and dimer. Finally, due to the large number of articles obtained, only articles reporting the isolation of new compounds, with their complete characterization and demonstrating cytotoxic activity, were selected. SciFinder provided the larger number of articles, while web of science and scopus provided comparable results. To highlight the most potent compounds with cytotoxic activity, only compounds with IC_50_ < 20 μM were considered, according to the classification of samples that define “good activity” or “very strong cytotoxicity” compounds with IC_50_ < 20 μM [156].

## 7. Conclusions

The isolation and characterization of new diterpenes compounds from higher plants has increased constantly from 1970, with a higher number of published articles in the years 2000–2017. Although, in the present review, we have observed a decreasing number of published articles from 2017, plants represent one of the most promising reservoir of compounds, often structurally complex, that could be useful in the treatment of cancer disease. In fact, many plant species are still unexplored—for example, the *Euphorbia* species, for which less than 5% has been studied until now [1]. In the present review, 251 new diterpenes that showed cytotoxic activity with IC_50_ < 20 μM have been described. These compounds could be further studied, in order to evaluate their use as potential anticancer drugs. The main skeletons that showed cytotoxic activity were: kaurane (29 compounds), abietane (17 compounds), clerodane (17 compounds), and labdane (8 compounds). The most active compounds were extracted from 29 different plant families; particularly, Euphorbiaceae (69 compounds) and Lamiaceae (54 compounds) were the richest sources of active compounds. Cytotoxicity of the compounds was verified on several cancer cell lines, especially against the most common cancer diseases, such as human non-small-cell lung carcinoma (A549 cell line), breast cancer (MCF-7 cell line), promyelocytic leukemia (HL-60 cell line), cervical carcinoma (HeLa cell line), colon cancer (SW480), and liver carcinoma (HepG2, Hep3B, and SMMC-7721 cell lines). A better activity than the positive control was obtained with 33 compounds against the A549 cell line, 28 compounds against the MCF-7 cell line, 9 compounds against the HepG2 cell line, 8 compounds against the Hep3B cell line, 19 compounds against the SMMC-7721 cell line, 9 compounds against the HL-60 cell line, 24 compounds against the SW480 cell line, and 19 compounds against HeLa. In general, the structure–activity relations still remain unclear, and it seems very difficult to find a correlation between the diterpene structures and cytotoxicity against a specific cancer cell line.

During our bibliographic research, we observed that many new discovered diterpenes have not been tested for their biological activities, sometimes for the low amount available, and were not considered in the present review. Not all the papers cited in the present review reported the cytotoxic activity against normal cell lines that we consider important to determine the selectivity against cancer cells. Moreover, the positive controls used were not the same, and values of cytotoxic activity better than the positive control cannot be completely compared. It should be useful, in our opinion, to define a protocol for anticancer activity, with a list of the most common cancer cell lines to be tested, as well as a definition of the positive control and assay to be used (Appendix A, Table A1).

## 8. Future Perspective

Although the number of papers published on new natural products isolation and characterization has decreased in the last few years, we think that this kind of work remains a very important topic to focus on, since cancer disease represents one of the major health problems in our society. Plants are able to synthesise complex structures that are difficult to obtain in the laboratory by chemical synthesis; so, plants are a good source for the development of new drugs. Moreover, a large amount of plants still remains unexplored and represents a reservoir of potentially active substances. One limit on this research may be the low amount of secondary metabolites that are usually isolated and the complex separation processes needed. Anyway, biotechnological techniques could overcome this problem and help to direct secondary metabolite biosynthesis towards the target product.

## Data Availability

Not applicable.

## Appendix A

**Table A1:** Cancer cell lines.

95-D	lung giant-cell carcinoma
A2780	human ovarian cancer
A375	human melanoma cancer
A549	human non-small-cell lung carcinoma
A549/CDDP	cisplatin resistant human adenocarcinomic alveolar basal epithelial
A549/Taxol	taxol resistant lung cancer
ACHN	human kidney cancer cells
ACP01	gastric cancer
AGS	gastric cancer
B16F10	melanoma
B16 melanoma 4A5	theophylline-stimulated murine melanoma
BGC-823	human gastric cancer
BT474	breast ductal carcinoma
C26	colon cancer
C4-2B	human prostate cancer
C4-2B/ENZR	enzalutamide-resistant C4-2B cell line
CaCo2	human colorectal carcinoma
CCRF-CEM	drug-sensitive leukemia
CEM/ADR5000	multidrug resistant P-glycoprotein-overexpressing subline
Chago-K1	undifferentiated lung carcinoma
DU145	human prostate cancer
EC	esophageal cancer
EC109	human esophageal cancer
EC9706	human esophageal cancer
ECC-1	endometrial carcinoma
HCT-15	human colon cancer
HCT-15/5-FU	fluorouracil resistant human colorectal adenocarcinoma
HCT-116	colon cancer
HEL	human erythroleukemia
HeLa	human cervical carcinoma
HepG2	human hepatocellular liver carcinoma
HepG2/DOX	doxorubicin-resistant human hepatocellular liver carcinoma
Hep3B	human liver cancer
HEY	human ovarian cancer
HL-60	human erythroleukemia (promyelocytic leukemia)
HMy2.CIR	human B lymphoblast
HT29	human colon cancer
HMCB	human melanoma cancer
Huh-7	human hepatoma
Jurkat	T-cell lymphoma
K562	human erythromyeloblastoid leukemia
KATO-III	gastric carcinoma
KB	human epithelial carcinoma
KB-VIN	P-gp-overexpressing MDR subline of KB
KYSE-450	human esophageal cancer
L5178Y	mouse lymphoma
L6	rat myoblast
LoVo	human colon cancer
LUPF003	non-small-cell lung
LUPF045	nonsmall-cell lung
MCF-7	breast cancer
MDA-MB-231	human breast carcinoma (triple-negative breast cancer)
MDA-MB-435	melanoma
MDA-MB-453	breast cancer
MG-63	human osteosarcoma
MM-231	breast cancer
MM96L	melanoma
MM-CSCs	human multiple myeloma cancer stem
MOLT-4	human lymphoblastic leukemia
MSTO-211H	human lung cancer
MT-1	human contaminated breast cancer
MV4-11	lymphoblast from human biphenotypic B myelomonocytic leukemia
Namalwa	Burkitt lymphoma
NB4	human acute promyelocytic leukemia cell
NCI-H1229	lung large cell carcinoma
NCI-H1650	human lung adenocarcinoma bronchioalveolar carcinoma
NCI-H1975	human lung adenocarcinoma cells
NCI-H460	non-small cell lung carcinoma
OVPF008	ovarian cancer
OVPF038	ovarian cancer
p388D1	mouse leukemia
PANC-1	human pancreatic
PC3	human prostate cancer
PC-9	lung cancer
RKO	human colon cancer
Saos-2	human osteosarcoma
SGC7901	human gastric cancer
SHG-44	human glioblastoma
SK-BR-3	human breast cancer
SK-Hep-1	liver cancer cell lines
SKLU-1	human lung adenocarcinoma
SK-Mel2	human melanoma
SKOV3	human ovarian cancer
SMMC-7721	hepatoma cancer
SNU638	stomach cancer cell lines
SW1990	human pancreatic cancer
SW480	human colon cancer
SW620	human colon cancer
T24	human urinary bladder cancer
U2OS	human osteosarcoma
U87	glioblastoma
U87-TxR	resistant glioblastoma
U87-MG	glioblastoma
U-251	human glioblastoma
U937	human monoblastic leukemia
ZR-75-1	epithelial from the mammary gland
XWLC-05	lung adenocarcinoma
